# RStrucFam: a web server to associate structure and cognate RNA for RNA-binding proteins from sequence information

**DOI:** 10.1186/s12859-016-1289-x

**Published:** 2016-10-07

**Authors:** Pritha Ghosh, Oommen K. Mathew, Ramanathan Sowdhamini

**Affiliations:** 1 National Centre for Biological Sciences, Tata Institute of Fundamental Research, Bellary Road, Bangalore, Karnataka 560 065 India; 2 SASTRA University, Tirumalaisamudram, Thanjavur, 613401 Tamil Nadu India

## Abstract

**Background:**

RNA-binding proteins (RBPs) interact with their cognate RNA(s) to form large biomolecular assemblies. They are versatile in their functionality and are involved in a myriad of processes inside the cell. RBPs with similar structural features and common biological functions are grouped together into families and superfamilies. It will be useful to obtain an early understanding and association of RNA-binding property of sequences of gene products. Here, we report a web server, RStrucFam, to predict the structure, type of cognate RNA(s) and function(s) of proteins, where possible, from mere sequence information.

**Results:**

The web server employs Hidden Markov Model scan (hmmscan) to enable association to a back-end database of structural and sequence families. The database (HMMRBP) comprises of 437 HMMs of RBP families of known structure that have been generated using structure-based sequence alignments and 746 sequence-centric RBP family HMMs. The input protein sequence is associated with structural or sequence domain families, if structure or sequence signatures exist. In case of association of the protein with a family of known structures, output features like, multiple structure-based sequence alignment (MSSA) of the query with all others members of that family is provided. Further, cognate RNA partner(s) for that protein, Gene Ontology (GO) annotations, if any and a homology model of the protein can be obtained. The users can also browse through the database for details pertaining to each family, protein or RNA and their related information based on keyword search or RNA motif search.

**Conclusions:**

RStrucFam is a web server that exploits structurally conserved features of RBPs, derived from known family members and imprinted in mathematical profiles, to predict putative RBPs from sequence information. Proteins that fail to associate with such structure-centric families are further queried against the sequence-centric RBP family HMMs in the HMMRBP database. Further, all other essential information pertaining to an RBP, like overall function annotations, are provided. The web server can be accessed at the following link: http://caps.ncbs.res.in/rstrucfam.

**Electronic supplementary material:**

The online version of this article (doi:10.1186/s12859-016-1289-x) contains supplementary material, which is available to authorized users.

## Background

RNA-binding proteins (RBPs) interact with their cognate RNAs to form biomolecular assemblies called as ribonucleoprotein (RNP) complexes which may be transient (such as the exon junction complex) or stable (such as the ribosome). The biological functions of proteins can be better understood by grouping them into domain families based on the analysis of their structural features [1, 2]. The realisation of connections to structural domains of known function can help to predict the mechanism(s) of RNA binding in RBPs and also the type of cognate RNA. The number of members in a structural domain family reflects the diversity and evolutionary ability of that family to adapt to biological contexts [3]. This, however, cannot be generalised since certain protein structures are more difficult to solve as compared to others.

A comprehensive analysis of RNA-protein interactions at the atomic and residue levels was performed by Jones and coworkers in 2001, with a dataset of 32 RNA-protein complexes (solved by either X-ray crystallography or Nuclear Magnetic Resonance (NMR) spectroscopy) that were available in the Nucleic Acid Database (NDB) [4] in December 1999. This led to a classification of RBPs into 14 structural families [5]. In 2004, Han and coworkers had trained a Support Vector Machine (SVM) system to recognise RBPs directly from their primary sequence on the basis of knowledge of known RBPs and non-RBPs [6].

The BindN web tool, introduced in 2006, employed SVM models to predict potential DNA-binding and RNA-binding residues from amino acid sequence [7]. In 2008, Shazman and coworkers classified RBPs on the basis of their three-dimensional structures by using a SVM approach [8]. Their dataset comprised of 76 RNA-protein complexes (solved by either X-ray crystallography or NMR) that were then available in the PDB. The method had achieved 88 % accuracy in classifying RBPs, but could not distinguish them from DNA-binding proteins (DBPs) and was based on the characterization of the unique properties of electrostatic patches in these proteins. Shazman and coworkers had trained the multi-class SVM classifier on transfer RNA (tRNA)-, ribosomal RNA (rRNA)- and messenger RNA (mRNA)-binding proteins only.

In 2010, Kazan and coworkers introduced a motif-finding algorithm named RNAcontext, that was designed to elucidate RBP-specific sequence and structural preferences with a high accuracy [9]. Two years later, Jahandideh and coworkers used the Gene Ontology Annotated (GOA) database (available at http://www.ebi.ac.uk/GOA) and the Structural Classification of Proteins (SCOP) database [10], to design a machine learning approach for classifying structurally solved RNA-binding domains (RBDs) in different subclasses [11].

The catRAPID omics web server introduced in 2013, performed calculation of ribonucleoprotein associations like analysis of nucleic acid-binding regions in proteins and identification of RNA motifs involved in protein recognition in different model organisms [12]. It included binding residues and evolutionary information for prediction of RBPs. In 2014, Fukunaga and coworkers proposed the CapR algorithm for studying RNA-protein interactions using CLIP-seq data [13]. The authors had shown that several RBPs bind RNA based on specific structural contexts. RBPmap, the newest of the above-mentioned methods, was used for prediction and mapping of RBP-binding sites on RNA [14].

In 2011, a collection of RNA-binding sites on the basis of RBDs were made available in a database named RBPDB (RNA-binding protein database) [15]. Two of the recent repositories, RAID (RNA-associated interaction database) [16] and ViRBase (virus–host ncRNA-associated interaction database) [17], described RNA-associated (RNA-RNA/RNA-protein) interactions and virus-host ncRNA-associated interactions respectively. The NPIDB (Nucleic acid-Protein interaction database) [18] and BIPA (Biological interaction database for protein-nucleic acid) [19] are also well-known databases on the structural front. However, these repositories can offer information about those for which structural data are available.

Since an increasing number of protein structures are being solved every day, there arises a need to design an automated protocol for classifying the new structures into families that, will in turn, provide an insight into the putative functions of these newer proteins. Most of the previous studies had employed machine learning algorithms to predict or classify RBPs [6–8, 11, 20, 21]. Electrostatic properties of the solvent accessible surface were used as one of the primary features in such machine learning algorithms. This property was very different even among proteins with very similar structures and functions [22].

Here, we report a web server, RStrucFam, which to the best of our knowledge is the first of its kind that exploits structurally conserved features, derived from family members with known structures and imprinted in mathematical profiles, to predict the structure, the type of cognate RNA(s) (not only tRNA, rRNA or mRNA but also to the other kinds of RNA that are currently known) and function(s) of proteins from mere sequence information. The user input protein sequence will be searched against the Hidden Markov Models of RBP families (HMMRBP) database comprising of 437 HMMs of RBP structural families that have been generated using structure-based sequence alignments of RBPs with known structures. Proteins that fail to associate with such structure-centric families will be further queried against the 746 sequence-centric RBP family HMMs in the HMMRBP database. The search protocol has been previously employed in the lab for prediction of RBPs in humans on a genome-wide scale [23]. The users can browse through the HMMRBP database for details pertaining to each family, protein or RNA and their related information, based on keyword search or RNA motif search. RStrucFam web server is distinct from searches possible within the PDB, Structural Classification of Proteins (SCOP) [10], SCOP extended (SCOPe) [24] and the Protein Alignments organised as Structural Superfamilies 2 (PASS2) [25] resources, in being able to identify or classify RBPs even *without a known structure*, as well as prediction of cognate RNA(s) and function(s) of the protein from *mere sequence information*. RStrucFam can be accessed at http://caps.ncbs.res.in/rstrucfam/.

## Implementation

### HMMRBP database

1285 RNA-protein and 14 DNA/RNA hybrid-protein complexes were retrieved from the PDB (May 2015 version). The scheme for the classification of the RBP chains from these complexes and the method for generating the HMMs have been described in our previous study [23]. Level 1 of the HMMRBP database consists of 437 structure-centric family HMMs. All X-ray crystal structures (without any resolution cut-off) and the first models of the NMR ensembles were considered for our analysis, but PDBs that have been split to sub-PDBs and indexed were not included in the dataset. The HMMs were built and converted to a binary format using the hmmbuild and hmmpress modules of HMMER3.1b1 suite [26]. Level 2 of the database consists of 746 sequence-centric RBP family HMMs retrieved from the Pfam 28 [27] database based on a keyword search followed by manual curation.

### Annotations

The annotations available for proteins present in this database have been described below.

### Structural alignment and phylogeny

Multiple structure-based sequence alignments (MSSA) and superposed structures of members belonging to each structural family were obtained using the in-house structure-based sequence alignment tool named COMPARER [28] and implemented in our PASS2 database [25]. Structural phylogeny of members belonging to each structural family were obtained using Matt [29]. The MSSA and hence the HMMs for each family are dependent on the PDB structures. In cases where there are incomplete residues in the PDB structures, initial equivalences cannot be derived by the JOY program [30] and hence such residues are removed for smooth completion of the COMPARER alignment protocol. This leads to generation of HMMs that are smaller than the actual size of the protein and encodes lesser information. Such shortened HMMs might, in turn, fail to identify proteins that are true homologues of that particular family.

### RNA-binding regions (RBRs)

The protein residues that are within 5 Å distance from an RNA chain in a RNA-protein complex and hence capable of interacting with the RNA, form the RNA-binding region (RBR) of the protein. Such residues have now been provided as a list for each protein chain. RBRs are a subset of functionally important residues (FIRs) for a particular protein family. The approach involves the calculation of all-against-all atomic distances among protein and RNA atoms in a RNA-protein complex and residues within a 5 Å cut-off distance from any atom in the RNA chain are designated as ‘RNA-binding’. Such calculations are very computationally intensive and hence can be technically challenging for larger RNA-protein complexes like the ribosome.

### Absolutely conserved residues (ACRs)

Residues that are conserved across all the members of a family have been highlighted in yellow in the MSSAs of the structural families, wherever applicable and defined as absolutely conserved residues (ACRs). ACRs can provide hints at important regions from the perspective of a protein family and may constitute a subset of the FIRs. The families which have seven or more members have been considered for ACR mapping.

### Gene Ontology (GO) mapping

Each member of a family has been assigned with GO term(s) [31] which were retrieved dynamically from www.rcsb.org using the RestFul API clients written in Python and signify the putative function(s) of the protein.

### RNA-protein interactions

The amino acids involved in binding RNA and the kind of interactions that occur within the protein residues and the bases and/or sugar-phosphate backbone of the RNA have been examined using the HBPLUS [32] and NUCPLOT programs [33]. NUCPLOT automatically identifies such interactions from a PDB file of the RNA-protein complex, and plots a schematic representation of the same.

### Search protocol

The hmmscan module from the HMMER3.1b1 package [26] has been used in RStrucFam for comparison of the user input protein sequence with the HMMRBP database. Structural or sequence family or families are assigned to the protein if the connections happen within permitted E-values. The default E-value for the search protocol is 10^−3^, but the users can also modify the search by changing the E-value threshold as per necessity.

### Validations

As mentioned earlier, the protocol has been previously used to successfully predict the entire repertoire of RBPs in the human proteome [23]. The search method has been validated with a negative test set of 100 proteins, comprising of a few DBPs and other non-nucleic acid-binding proteins. Resubstitution test has been performed using a randomly selected subset of 100 proteins of the initial dataset. Both the searches were carried out at a sequence E-value cut-off of 10^−3^. The raw output files for the searches with details on domain i-Evalue and scores are available at http://caps.ncbs.res.in/download/rstrucfam.

## Results

**Fig. 1:**
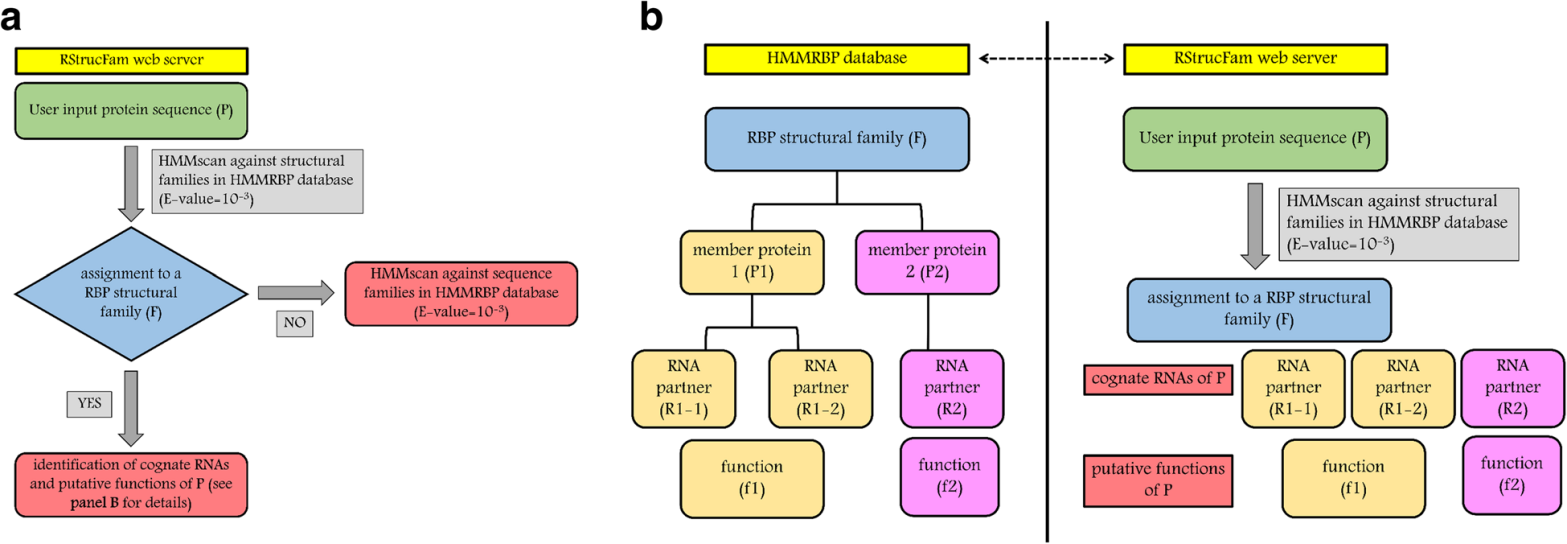
Schematic representation of RStrucFam protocol. **a** The user input protein sequence (P) will be searched against the structure-based RBP family HMMs in HMMRBP using the hmmscan module of the HMMER package at a default E-value of 10^−3^. If the protein fails to associate with any such family, then it is further queried against the HMMs of sequence domain families in HMMRBP. Proteins that identify hits in the structural family space, are assigned with cognate RNAs and putative functions as described in detail in panel (**b**). **b** RStrucFam provides a list of the possible RBP structural families identified in the search, from which the user can select the best hit based on domain E-value, score and alignment with all other members of the family. This family has been designated as F. Search in the HMMRBP database shows that F has two members - proteins P1 and P2. P1 binds to cognate RNAs R1-1 and R1-2, and performs a function f1, whereas P2 binds to the RNA R2 and performs a function f2. It is been observed that R1-1, R1-2 and R2 are similar kinds of RNAs (see text and Additional file 1), and f1 and f2 are similar kinds of functions. Based on this observation, from mere sequence information, it can be inferred that protein P (assigned to the family F) is also capable of binding the RNAs R1-1, R1-2, R2 and perform the functions f1 and f2.

A schematic representation of the RStrucFam protocol is shown in Fig. 1. All the essential information pertaining to RBPs (like structures, cognate RNAs and putative functions that can be directly retrieved for proteins with structures solved in complex with RNA, or predicted for proteins without known structures or those solved in RNA-free form) can be obtained.

### HMMRBP database

The database component of RStrucFam, called HMMRBP, holds all the information pertaining to the families that define the search space for the web server. The users can browse through the details for each of these 444 structural families, 746 sequence families and proteins comprising them, based on keyword search or for RNAs associated with the proteins based on keyword or sequence motif search.

**Fig. 2:**
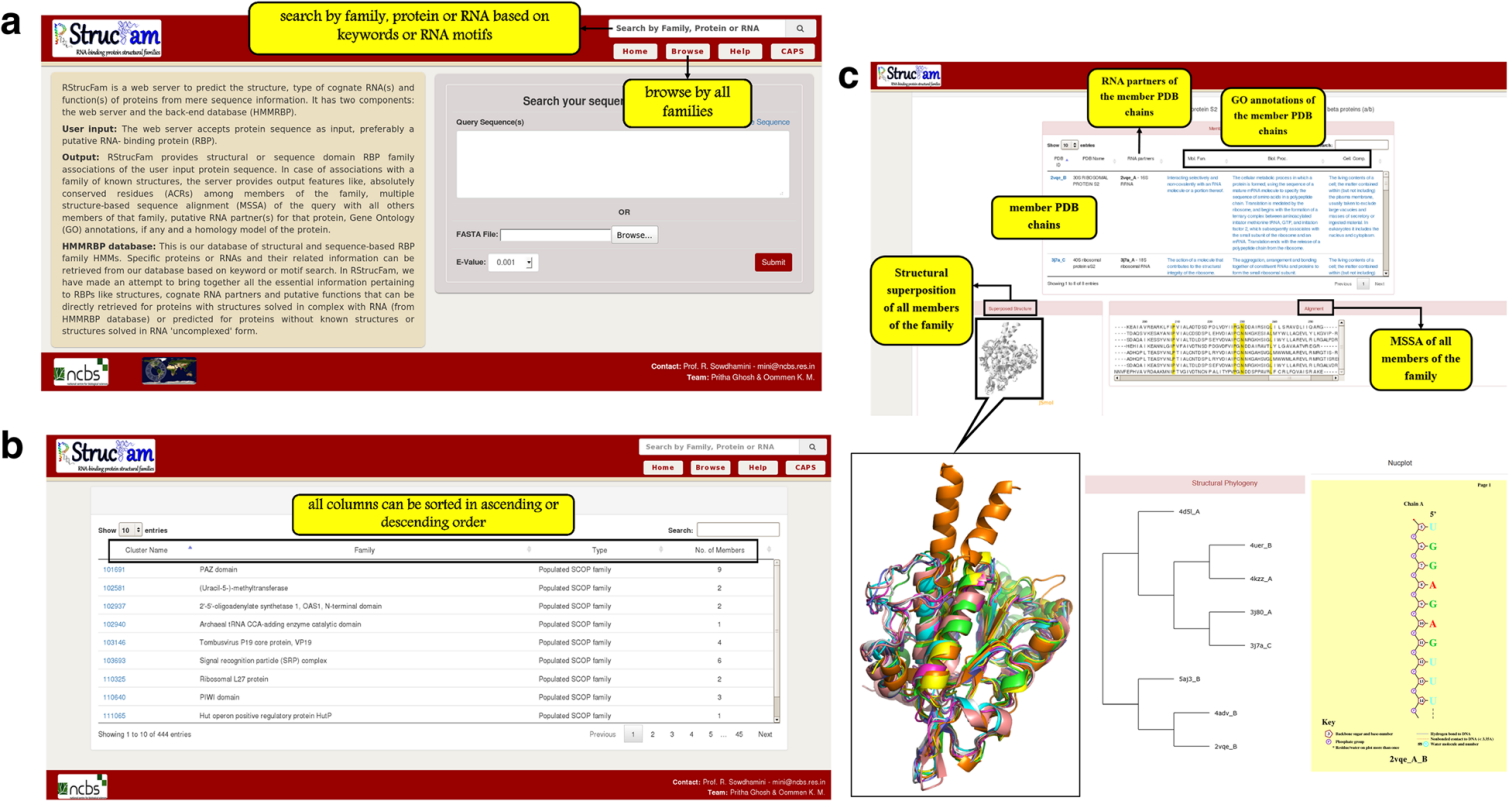
Snapshots from the HMMRBP database. Different features of the database have been shown here. **a** Database browser. The users can browse through the HMMRBP database for details pertaining to each family, protein or RNA and their related information, based on keyword search or RNA motif search in the ‘search’ tool box. The database can also be browsed through a list of families from the ‘browse’ button. **b** List of families in the database. A list of all the 444 structural families and 746 Pfam families that are present in this database, along with their associated details have been provided. This list can be sorted in ascending or descending order based on the family id, name, type and the number of members. **c** Details of each family. Features pertaining to each family (hierarchy of the family, cognate RNAs, GO functions, superposed structures and structural phylogeny of all the members, MSSA, RBRs and NUCPLOT for each member) can be visualised in each family-specific page. Residues that are 100 % conserved among all the member PDB chains in the family (ACRs) are highlighted in yellow in the alignment

HMMRBP holds information for all the families that constitute the database. Information on the structural families includes hierarchy of the family (wherever applicable), PDB chain ids and names of the proteins that comprise that particular family and those for their cognate RNAs, GO annotations (molecular function, biological process and cellular component), MSSA, RBRs, ACRs, NUCPLOT, superposed structure and structural phylogeny of the member proteins. The structural phylogeny provides an overall picture of the structural conservation within the members of a family and is highly dependent on the nature of the available structures. Where a part of the protein chain cannot be determined due to experimental conditions and/or local conformational flexibility, the structural phylogeny could be affected. Schematic representation of the RNA-protein interactions also has been made available for the family members and these representations are generated using the NUCPLOT. Figure 2 shows screenshots from the database.

### RStrucFam web server

**Fig. 3:**
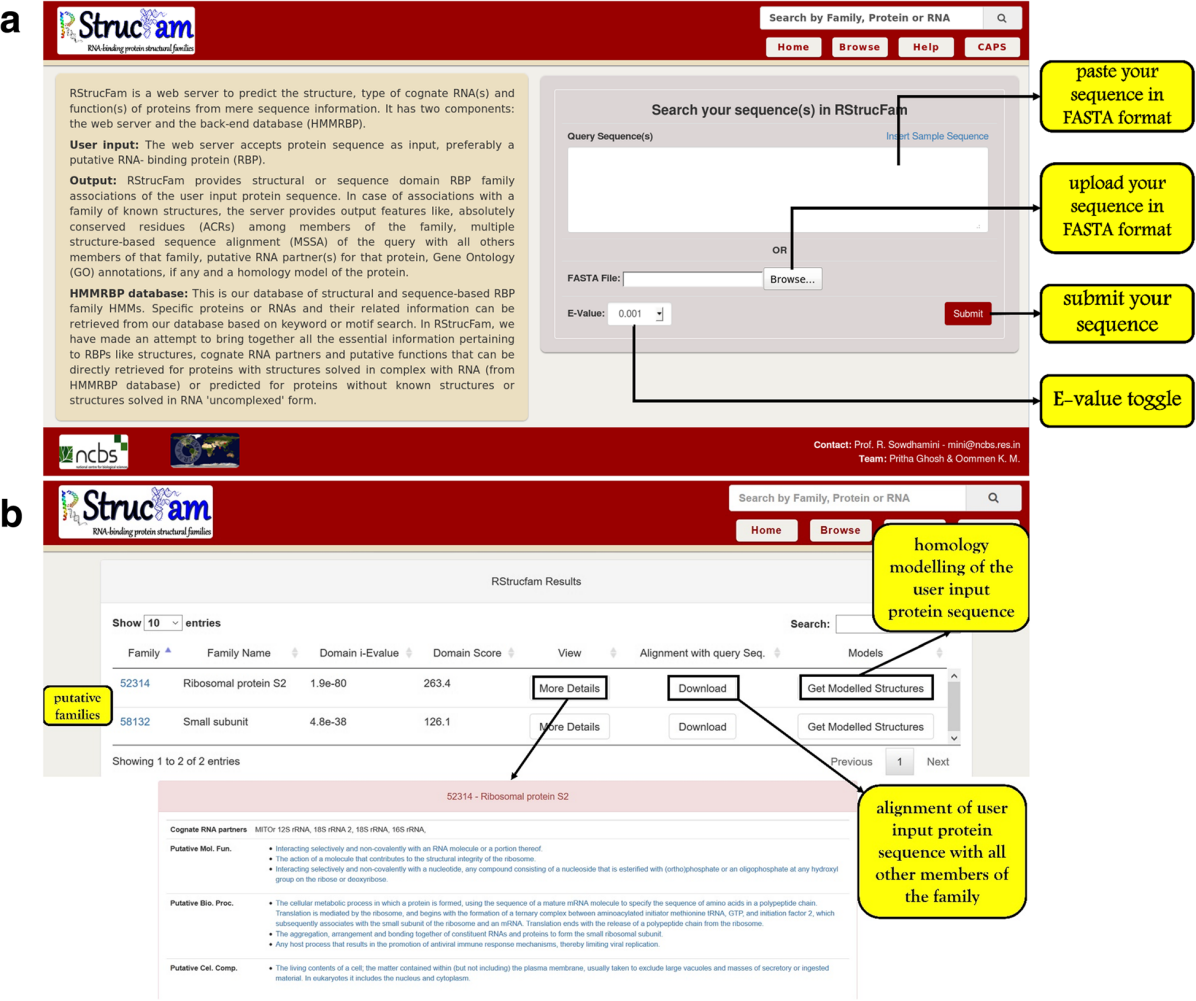
Snapshots from the RStrucFam web server for an example run. **a** Sequence input. Users may provide their input sequence either by pasting the sequence in FASTA format in the ‘query sequence’ box or by uploading a file containing the sequence in the same format. The E-value for the search can be modified by the user. **b** Search results page. A snapshot of the search output page shows that the sequence can be putative member of either of the two families listed. The best possible family for the protein can be selected on the basis of E-value, score and alignment with all other members of the family. The structure of the user input protein sequence may also be modelled based on the structures of the other members of the family. The output page also lists the putative cognate RNAs suggesting fine-tuned function of the protein of interest

The RStrucFam web server assigns families to RBPs from mere sequence information. The approach works at two successive levels. Firstly, it accepts protein sequence as input, and searches against our database of structural family HMMs. Secondly, user input proteins that fail to associate with such structure-centric families are further queried against the sequence-centric HMMs in the HMMRBP database. Associations to a structural family provides output features like MSSA of the query with all others members of that family, putative cognate RNAs for that protein, GO annotations, if any and a homology model of the protein. The assignment of a protein to an existing structural family helps to predict the putative RNA partner(s) and functions of the protein, based on the observation that members of the same structural family bind to similar RNAs (Additional file 1) and perform similar functions. Hence, this method can guide the user to predict the structure, function(s) and RNA partner(s) of a protein with considerable level of confidence. On the other hand, if a RNA-binding function(s) is not known for the query, RNA-binding could be inferred through homology with any of the known RBPs, as identified by RStrucFam. Figure 3 shows a screenshot of the web server.

### Validations

The sequence search tools and protocol within RStrucFam web server have been validated with a negative test set of 100 proteins (not known to bind to RNA) out of which 42 proteins were known to bind DNA. RStrucFam could be employed to successfully discard such DBPs as false positives (please see Additional file 2 for details). Further, a randomly selected subset of proteins from our initial dataset were queried against the HMM libraries of structural families. Such resubstitution tests showed that 93 % of the proteins could be associated with their parent families (Additional file 3). The lack of association of 7 % of the proteins to their parent families may be attributed to a large sequence identity spread among its members of those families. Such a high sequence identity spread may arise due pure sequence dispersion or occasionally due to the presence of unknown (UNK) residues in the PDBs constituting a family.

## Conclusions

The understanding of nucleic acid-protein interactions has been a coveted knowledge in the field of biology. The number of RNA-protein complex structures available in the PDB is much less as compared to DNA-protein complexes, which poses a hurdle in understanding RNA-protein interactions. In this paper, we report the availability of a web server to identify the RNA-binding mechanism(s) of a protein from mere sequence information based on a standardised protocol and a specialised database of RBPs. Where possible, such proteins are also assigned a structure and putative function(s). The HMMRBP database also permits users to visualise features of proteins and RNAs in existing RNA-protein complexes.

It is possible to use the web server to identify RNA-binding properties of a putative RBP from sequence information, even when structural information is unavailable. Hence, it is different from the other existing methods, like Basic Local Alignment Search Tool (BLAST) against the PDB and sequence-versus-Pfam HMM searches. In RStrucFam, the users can query their protein sequences against profiles generated from families of related structures, unlike performing BLAST against the PDB, where an user can query their sequence(s) against only one structure at a time. Hence our tool has the advantage of providing a greater sampling space by using mathematical profiles generated from structural or sequence information available from multiple proteins, as opposed to the use of single target proteins by the other related resources. Even though a similar concept of profiles exists in Pfam, the method of generation of the profiles is conceptually different between Pfam and RStrucFam. Pfam HMMs are generated based on sequence alignment, whereas the HMMs in RStrucFam encode structure-based sequence alignment information. Therefore, unlike in our method, the user will not be able to obtain information related to the structure or cognate RNA partners of the proteins by searching against the Pfam database. Thus, our tool has an advantage over the others in being able to combine both the use of mathematical profiles as well as structural information.

The HMMRBP database provides detailed information regarding RBRs in known RBPs and the interactions made by residues in such regions with RNA. Such information will also help the users to deduce the probable RBRs and interactions in their proteins of interest by comparing with members of the related structural families. To the best of our knowledge, no existing tool provides information regarding cognate RNA partners for putative RBPs in the absence of structural data. However, it is not possible to identify novel RNA-binding proteins using this web server i.e., if there are no structures with bound RNA, similar to the protein of interest in the PDB or if similar sequences were not previously reported to bind RNA, and hence such a sequence domain family is absent from the Pfam database. We believe that RStrucFam will be helpful to the biological community to overcome the shortcomings arising out of the limited availability of RNA-protein complex structures.

## Additional files

Additional file 1: Family-specific list of RNA that bind to protein chains belonging to the family. (DOC 1776 kb)
Additional file 2: Details of proteins used as the negative test set. (DOC 150 kb)
Additional file 3: List of proteins used in the resubstitution test. (DOC 102 kb)

## Abbreviations

ACR: Absolutely conserved residue
BLAST: Basic Local Alignment Search Tool
DBP: DNA-binding protein
FIR: Functionally important residue
GO: Gene Ontology
HMM: Hidden Markov Model
HMMRBP: Hidden Markov Models of RNA-binding protein families
mRNA: Messenger RNA
MSSA: Multiple structure-based sequence alignment
PASS2: Protein Alignments organised as Structural Superfamilies 2
PDB: Protein Data Bank
Pfam: Protein Families database
RBP: RNA-binding protein
RBR: RNA-binding region
RNP: Ribonucleoprotein
rRNA: Ribosomal RNA
SCOP: Structural Classification of Proteins
SCOPe: Structural Classification of Proteins extended
SVM: Support Vector Machine
tRNA: Transfer RNA
