# Supplementary material for: RStrucFam: a web server to associate structure and cognate RNA for RNA-binding proteins from sequence information
Source: BMC Bioinformatics. 2016 Oct 7;17:411. doi: 10.1186/s12859-016-1289-x (PMC5054549; doi:10.1186/s12859-016-1289-x)
Supplement: Additional file 1: — Family-specific list of RNA that bind to protein chains belonging to the family. (DOC 1776 kb) [file 12859_2016_1289_MOESM1_ESM.doc]

**Additional File 1:** Family-specific list of RNA that bind to protein chains belonging to the family.

| **Family id** | **Family name** | **Names of RNA chains** |
| --- | --- | --- |
|  | | |
| 254308 | automated matches | 3nvi_E: RNA (5'- R(*CP*UP*CP*UP*GP*AP*CP*CP*GP*AP*AP*AP*GP*GP*CP*GP*UP*GP*AP*UP*GP*AP*GP*C)-3') |
|  |  | 3nvk_L: RNA (5'- R(*GP*CP*CP*GP*UP*UP*GP*AP*AP*GP*CP*UP*CP*UP*GP*AP*CP*CP*GP*AP*AP*AP*GP*GP*CP*GP*UP*GP*AP*UP*GP*AP*GP*C)-3') |
|  |  | 3pla_G: C/D guide RNA |
|  |  | 3pla_H: C/D guide RNA |
|  |  | 3pla_J: RNA (5'-R(*CP*CP*AP*UP*GP*AP*GP*UP*GP*U)-3') |
|  |  | 3pla_N: C/D guide RNA |
|  |  | 3pla_O: RNA (5'-R(*CP*CP*AP*UP*GP*AP*GP*UP*GP*U)-3') |
|  | | |
| 52167 | Ribosomal protein L4 | 2rdo_B: 23S RIBOSOMAL RNA |
|  |  | 1ffk_0: 23S RRNA |
|  |  | 1ffk_9: 5S RRNA |
|  |  | 2zjp_X: RIBOSOMAL 23S RNA |
|  |  | 1jj2_0: 23S RRNA |
|  |  | 1jj2_9: 5S RRNA |
|  |  | 3cpw_0: 23S RIBOSOMAL RNA |
|  |  | 3cpw_9: 5S RIBOSOMAL RNA |
|  |  | 4wwe_1: 23S RRNA (2899-MER) |
|  | | |
| 47974 | Ribosomal protein S7 | 1fjg_A: 16S RIBOSOMAL RNA |
|  |  | 1fjg_X: FRAGMENT OF MESSENGER RNA |
|  |  | 2noq_A: CrPV IRES |
|  |  | 2noq_B: 18S ribosomal RNA |
|  |  | 2noq_C: 18S ribosomal RNA |
|  |  | 4adv_A: 16S RIBOSOMAL RNA |
|  |  | 5aj3_A: MITORIBOSOMAL 12S RRNA |
|  | | |
| 64490 | RNA-polymerase beta-prime | 2b63_R: 31-MER |
|  | | |
| 102940 | Archaeal tRNA CCA-adding enzyme catalytic domain | 4x4n_B: G70A tRNA minihelix |
|  |  | 4x4n_G: G70A tRNA minihelix |
|  | | |
| 50152 | BglG-like antiterminator proteins | 1l1c_C: licT mRNA antiterminator hairpin |
|  | | |
| 81268 | Tandem AAA-ATPase domain | 2hyi_F: 5'-R(*UP*UP*UP*UP*UP*U)-3' |
|  |  | 4d25_D: 5'-R(*UP*GP*AP*CP*AP*UP)-3' |
|  |  | 2db3_E: 5'-R(*UP*UP*UP*UP*UP*UP*UP*UP*UP*U)-3' |
|  |  | 2db3_G: 5'-R(*UP*UP*UP*UP*UP*UP*UP*UP*UP*U)-3' |
|  |  | 4tyw_B: RNA (5'-R(P*AP*AP*AP*AP*AP*AP*A)-3') |
|  |  | 3i61_B: 5'-R(*UP*UP*UP*UP*UP*UP*UP*UP*UP*U)-3' |
|  |  | 2xgj_C: RNA (5'-(*AP*AP*AP*AP*A)-3') |
|  |  | 2xgj_D: RNA (5'-(*AP*AP*AP*AP*A)-3') |
|  |  | 3sqw_B: RNA (5'-R(P*UP*UP*UP*UP*UP*UP*UP*UP*UP*U)-3') |
|  | | |
| 58327 | HIV-1 REV fragments | 1i9f_A: REV RESPONSE ELEMENT RNA |
|  |  | 1etf_A: REV RESPONSE ELEMENT RNA |
|  | | |
| 191451 | automated matches | 3ftf_C: 5'-R(P*AP*AP*CP*CP*GP*UP*AP*GP*GP*GP*GP*AP*AP*CP*CP*UP*GP*CP*GP*GP*UP*U)-3' |
|  |  | 3ftf_D: 5'-R(P*AP*AP*CP*CP*GP*UP*AP*GP*GP*GP*GP*AP*AP*CP*CP*UP*GP*CP*GP*GP*UP*U)-3' |
|  |  | 4adv_A: 16S RIBOSOMAL RNA |
|  | | |
| 57668 | Classic zinc finger, C2H2 | 1un6_F: 5S RIBOSOMAL RNA |
|  | | |
| 56694 | RNA-dependent RNA-polymerase | 2e9r_A: 5'-R(*CP*AP*UP*GP*GP*GP*CP*CP*C)-3' |
|  |  | 2e9r_B: 5'-R(*CP*CP*C*GP*GP*GP*CP*CP*C)-3' |
|  |  | 4k50_B: RNA (33-MER) |
|  |  | 4k50_C: RNA (5'-R(P*GP*CP*CP*CP*GP*GP*AP*CP*GP*AP*GP*AP*GP*A)-3') |
|  |  | 4k50_G: RNA (5'-R(P*GP*CP*CP*CP*GP*GP*AP*CP*GP*AP*GP*AP*GP*A)-3') |
|  |  | 4k50_J: RNA (33-MER) |
|  |  | 4k50_K: RNA (5'-R(P*GP*CP*CP*CP*GP*GP*AP*CP*GP*AP*GP*AP*GP*A)-3') |
|  |  | 4k4z_B: RNA (5'- R(*AP*AP*GP*UP*CP*UP*CP*CP*AP*GP*GP*UP*CP*UP*CP*UP*CP*GP*UP*CP*GP*AP*AP*A)-3') |
|  |  | 4k4z_C: RNA (5'-R(*UP*GP*UP*UP*CP*GP*AP*CP*GP*AP*GP*AP*GP*A)-3') |
|  |  | 4k4z_D: RNA (5'-R(P*GP*GP*GP*AP*GP*AP*UP*GP*A)-3') |
|  |  | 4k4z_J: RNA (5'- R(*AP*AP*GP*UP*CP*UP*CP*CP*AP*GP*GP*UP*CP*UP*CP*UP*CP*GP*UP*CP*GP*AP*AP*A)-3') |
|  |  | 4k4z_K: RNA (5'-R(*UP*GP*UP*UP*CP*GP*AP*CP*GP*AP*GP*AP*GP*A)-3') |
|  |  | 3h5x_P: 5'-R(*UP*GP*CP*CP*CP*GP*GP*G)-3' |
|  |  | 3h5x_T: 5'-R(P*UP*GP*CP*CP*CP*GP*GP*GP*C)-3' |
|  |  | 3ol6_B: RNA (5'- R(*AP*AP*GP*UP*CP*UP*CP*CP*AP*GP*GP*UP*CP*UP*CP*UP*CP*GP*UP*CP*CP*GP*GP*AP*AP*A)-3') |
|  |  | 3ol6_C: RNA (5'-R(*GP*CP*CP*CP*GP*GP*AP*CP*GP*AP*GP*AP*GP*A)-3') |
|  |  | 3ol6_D: RNA (5'-R(*GP*GP*GP*AP*GP*AP*UP*GP*A)-3') |
|  |  | 3ol6_J: RNA (5'- R(*AP*AP*GP*UP*CP*UP*CP*CP*AP*GP*GP*UP*CP*UP*CP*UP*CP*GP*UP*CP*CP*GP*GP*AP*AP*A)-3') |
|  |  | 4k4w_B: RNA (5'- R(P*GP*GP*GP*AP*GP*AP*UP*GP*AP*AP*AP*GP*UP*CP*UP*CP*CP*AP*GP*GP*UP*CP*UP*CP*UP*CP*UP*CP*GP*UP*CP*GP*AP*AP*A)-3') |
|  |  | 4k4w_C: RNA (5'- R(*UP*GP*UP*UP*CP*GP*AP*CP*GP*AP*GP*AP*GP*AP*GP*AP*CP*C)-3') |
|  |  | 4k4u_B: RNA (5'- R(*AP*AP*GP*UP*CP*UP*CP*CP*AP*GP*GP*UP*CP*UP*CP*UP*CP*UP*CP*GP*UP*CP*GP*AP*AP*A)-3') |
|  |  | 4k4u_C: RNA (5'-R(*UP*GP*UP*UP*CP*GP*AP*CP*GP*AP*GP*AP*GP*AP*GP*A)-3') |
|  |  | 4k4u_D: RNA (5'-R(P*GP*GP*GP*GP*GP*AP*GP*AP*UP*GP*A)-3') |
|  |  | 3nma_B: 5'-R(*GP*GP*C)-3' |
|  |  | 3nma_C: 5'-R(P*CP*C)-3' |
|  |  | 3nl0_B: 5'-R(P*UP*GP*GP*GP*CP*CP*C)-3' |
|  |  | 3nl0_C: 5'-R(*G*GP*GP*CP*CP*C)-3' |
|  |  | 3klv_B: RNA (5'-R(*AP*UP*GP*GP*GP*CP*C)-3') |
|  |  | 3klv_C: RNA (5'-R(*GP*GP*CP*CP*C)-3') |
|  |  | 3kms_B: RNA (5'-R(*AP*UP*GP*GP*GP*CP*C)-3') |
|  |  | 3kms_C: RNA (5'-R(*GP*GP*CP*CP*C)-3') |
|  |  | 3koa_B: RNA (5'-R(P*AP*UP*GP*GP*GP*C)-3') |
|  |  | 3koa_C: RNA (5'-R(P*CP*CP*CP*G)-3') |
|  |  | 4e78_P: 5'-R(*U*AP*CP*CP*GP*(GDO))-3' |
|  |  | 4e78_T: 5'-R(*U*AP*CP*CP*GP*(GDO))-3' |
|  |  | 1nb7_E: 5'-R(*UP*UP*UP*U)-3' |
|  |  | 1nb7_F: 5'-R(*UP*UP*UP*U)-3' |
|  |  | 1n1h_B: 5'-R(*AP*UP*UP*AP*GP*C)-3' |
|  |  | 3a6p_J: pre-microRNA |
|  |  | 4wtd_P: RNA PRIMER TEMPLATE AUAAAUUU |
|  |  | 4wtd_T: RNA PRIMER TEMPLATE AUAAAUUU |
|  |  | 4wzq_B: RNA template |
|  |  | 4wzq_C: RNAprimer |
|  |  | 4wtk_P: RNA PRIMER GG |
|  |  | 4wtk_T: RNA TEMPLATE AGCC |
|  |  | 4x2b_B: RNA template |
|  |  | 4x2b_C: RNA primer |
|  |  | 4iqx_B: RNA (5'-R(*AP*UP*GP*GP*GP*CP*C)-3') |
|  |  | 4iqx_C: RNA (5'-R(*GP*GP*CP*CP*C)-3') |
|  |  | 2bbv_N: RNA (5'-R(*UP*CP*UP*UP*AP*UP*AP*UP*CP*U)-3') |
|  |  | 4lq3_R: 5'-R(P*GP*G)-3' |
|  |  | 4wzm_B: RNA template |
|  |  | 4wzm_C: RNA primer |
|  | | |
| 74732 | Ribosomal protein L20 | 1xbp_0: 23S RIBOSOMAL RNA |
|  |  | 2rdo_B: 23S RIBOSOMAL RNA |
|  | | |
| 56809 | Ribosomal protein L1 | 2om7_I: Fragment of23S rRNA (H42-44) |
|  |  | 2om7_J: Fragment of23S rRNA (H76) |
|  |  | 2om7_M: p/E-tRNA |
|  |  | 1u63_B: 49 NT FRAGMENT OF MRNA FOR L1 |
|  |  | 1u63_D: 49 NT FRAGMENT OF MRNA FOR L1 |
|  |  | 1mzp_B: fragment of 23S rRNA |
|  |  | 2hw8_B: 36-MER |
|  |  | 2vpl_B: FRAGMENT OF MRNA FOR L1-OPERON CONTAINING REG |
|  |  | 2vpl_D: FRAGMENT OF MRNA FOR L1-OPERON CONTAINING REG |
|  |  | 3umy_B: RNA (80-MER) |
|  |  | 3u4m_B: RNA (80-MER) |
|  |  | 4qvi_B: fragment of 23S rRNA |
|  |  | 4wwe_1: 23S RRNA (2899-MER) |
|  |  | 4wwe_3: A SITE TNA, E SITE TNA |
|  |  | 4qg3_B: fragment of 23S rRNA |
|  |  | 3u56_B: RNA (80-MER) |
|  |  | 2noq_A: CrPV IRES |
|  |  | 2noq_E: 25S ribosomal RNA |
|  |  | 4csu_B: 23S RRNA |
|  |  | 4wwt_1: 23S RRNA (2899-MER) |
|  |  | 4wwt_3: TRNA (76-MER) |
|  |  | 4wwt_V: DNA (55-MER) |
|  | | |
| 52162 | Ribosomal protein L13 | 1ffk_0: 23S RRNA |
|  |  | 1ffk_9: 5S RRNA |
|  |  | 1xbp_0: 23S RIBOSOMAL RNA |
|  |  | 3bbx_B: 23S ribosomal RNA |
|  |  | 3j3w_A: ribosome RNA 23S |
|  | | |
| 54929 | Canonical RBD | 1m5o_B: RNA HAIRPIN RIBOZYME |
|  |  | 1a9n_R: RNA (5'- R(*CP*CP*UP*GP*GP*UP*AP*UP*UP*GP*CP*AP*GP*UP*AP*CP*CP*UP*CP *CP*AP*GP* GP*U)-3') |
|  |  | 1aud_B: RNA 3UTR |
|  |  | 1fxl_B: 5'-R(P*UP*UP*UP*UP*AP*UP*UP*UP*U)-3' |
|  |  | 1b7f_P: RNA (5'-R(P*GP*UP*UP*GP*UP*UP*UP*UP*UP*UP*UP*U)-3') |
|  |  | 1b7f_Q: RNA (5'-R(P*GP*UP*UP*GP*UP*UP*UP*UP*UP*UP*UP*U)-3') |
|  |  | 2err_B: UGCAUGU |
|  |  | 2vop_B: 5'-R(*AP*UP*UP*UP*UP)-3' |
|  |  | 1zh5_C: 5'-R(*UP*GP*CP*UP*GP*UP*UP*UP*U)-3' |
|  |  | 1zh5_D: 5'-R(*UP*GP*CP*UP*GP*UP*UP*UP*U)-3' |
|  |  | 2kxn_A: 5'-R(*AP*AP*GP*AP*AP*C)-3' |
|  |  | 4pr6_B: HDV RIBOZYME SELF-CLEAVED |
|  |  | 2ad9_B: 5'-R(*CP*UP*CP*UP*CP*U)-3' |
|  |  | 2mgz_C: RNA (5'-R(*UP*GP*CP*AP*UP*GP*GP*UP*GP*UP*GP*C)-3') |
|  |  | 2adb_B: 5'-R(*CP*UP*CP*UP*CP*U)-3' |
|  |  | 1fnx_R: AU-RICH RNA ELEMENT |
|  |  | 1fje_A: SNRE RNA |
|  |  | 2adc_B: 5'-R(*CP*UP*CP*UP*CP*U)-3' |
|  |  | 2adc_C: 5'-R(*CP*UP*CP*UP*CP*U)-3' |
|  |  | 2fy1_B: S1A stem-loop RNA |
|  |  | 4qqb_P: msl2 mRNA |
|  |  | 2xnr_C: 5'-R(*UP*UP*CP*UP*UP*AP*UP*UP*CP*UP*UP*A)-3' |
|  |  | 4qu7_U: RNA 5'-(*AP*GP*GP*GP*AP*UP)-3' |
|  |  | 4qu7_X: RNA 5'-(*AP*GP*GP*GP*AP*UP)-3' |
|  |  | 2mxy_B: 5'-R(*AP*UP*UP*UP*UP*UP*C)-3' |
|  |  | 3moj_A: RNA (69-MER) |
|  |  | 2m8d_A: RNA (5'-R(*UP*GP*AP*AP*GP*GP*AP*C)-3') |
|  |  | 4c4w_H: TSKT-23 |
|  |  | 4yb1_R: RNA (91-MER) |
|  |  | 4pkd_V: U1 snRNA stem-loops 1 and 2 (55-MER) |
|  |  | 4wkr_C: 7SK GGHP4 (300-332) |
|  |  | 2xb2_R: RNA POLY-U-RIBONUCLEOTIDE |
|  |  | 4f02_B: RNA (5'-R(*AP*AP*AP*AP*AP*AP*AP*AP*AP*AP*A)-3') |
|  |  | 4f02_E: RNA (5'-R(*AP*AP*AP*AP*AP*AP*AP*AP*AP*AP*A)-3') |
|  | | |
| 141092 | Ribosomal protein L21p | 1xbp_0: 23S RIBOSOMAL RNA |
|  |  | 2j28_B: 23S RIBOSOMAL RNA |
|  | | |
| 58200 | Computational models partly based on experimental data | 1oln_C: RNA |
|  |  | 3j7z_A: 23S rRNA |
|  | | |
| 58132 | Small subunit | 1dv4_A: 16S RIBOSOMAL RNA |
|  |  | 1fka_A: 16S RIBOSOMAL RNA |
|  |  | 1qd7_A: CENTRAL FRAGMENT OF 16 S RNA |
|  |  | 2ykr_A: 16S RRNA |
|  |  | 3j0o_b: 40S ribosomal RNA fragment |
|  |  | 3j0o_d: 40S ribosomal RNA fragment |
|  |  | 3j0o_e: 40S ribosomal RNA fragment |
|  |  | 3j0o_E: 40S ribosomal RNA fragment |
|  |  | 3j0o_G: 40S ribosomal RNA fragment |
|  |  | 3j0o_w: mRNA fragment |
|  |  | 3j0o_W: tRNA |
|  |  | 3j6v_A: 28S ribosomal RNA, mitochondial |
|  |  | 3j7a_7: tRNA |
|  |  | 3j7a_A: 18S ribosomal RNA |
|  |  | 3j80_2: 18S rRNA |
|  |  | 4kzz_i: 18S Ribosomal RNA |
|  |  | 4kzz_j: initiator Met-RNA-i |
|  |  | 4kzz_k: mRNA |
|  |  | 4uer_A: 18S RRNA |
|  |  | 5aj3_A: MITORIBOSOMAL 12S RRNA |
|  | | |
| 46907 | Ribosomal protein L11, C-terminal domain | 1hc8_C: 58 NUCLEOTIDE RIBOSOMAL 23S RNA DOMAIN |
|  |  | 1hc8_D: 58 NUCLEOTIDE RIBOSOMAL 23S RNA DOMAIN |
|  |  | 3cma_0: 23S RIBOSOMAL RNA |
|  |  | 3cma_9: 5S RIBOSOMAL RNA |
|  |  | 1mms_C: 23S RIBOSOMAL RNA |
|  |  | 1mms_D: 23S RIBOSOMAL RNA |
|  |  | 1y39_D: 58 Nucleotide Ribosomal 23S RNA Domain |
|  |  | 4ioa_X: 23S ribosomal RNA |
|  |  | 3j8g_A: 5S rRNA |
|  |  | 3j8g_B: 23S rRNA |
|  |  | 3j3w_A: ribosome RNA 23S |
|  | | |
| 117888 | Ribosomal protein L16p | 2j28_A: 5S RIBOSOMAL RNA |
|  |  | 2j28_B: 23S RIBOSOMAL RNA |
|  |  | 2zjp_X: RIBOSOMAL 23S RNA |
|  |  | 2zjp_Z: RIBOSOMAL 5S RNA |
|  |  | 4wwt_1: 23S RRNA (2899-MER) |
|  |  | 4wwt_2: TRNA (5'- D(*AP*UP*CP*CP*CP*CP*GP*UP*GP*UP*CP*CP*UP*UP*GP*GP*UP*UP*CP*G)-3') |
|  |  | 4wwt_4: TRNA (76-MER) |
|  |  | 4wwt_s: THERMUS THERMOPHILUS HB8 GENOMIC DNA, COMPLETE GENOME |
|  |  | 4wwt_V: DNA (55-MER) |
|  | | |
| 48663 | Ribosomal protein L39e | 1ffk_0: 23S RRNA |
|  |  | 1ffk_9: 5S RRNA |
|  |  | 1jj2_0: 23S RRNA |
|  |  | 1jj2_9: 5S RRNA |
|  |  | 1s72_0: 23S ribosomal RNA |
|  |  | 1s72_9: 5S ribosomal RNA |
|  | | |
| 54763 | Signal recognition particle alu RNA binding heterodimer, SRP9/14 | 1e8s_C: 7SL RNA, 88-MER |
|  |  | 1e8o_E: 7SL RNA |
|  |  | 4uyj_S: SRP RNA |
|  | | |
| 143916 | RNase Z-like | 2fk6_R: TRNA(THR) |
|  |  | 4gcw_B: TRNA(THR) |
|  | | |
| 63611 | Pumilio repeat | 3q0o_C: 5'-R(UP*GP*UP*AP*CP*AP*UP*C)-3' |
|  |  | 3q0o_D: 5'-R(UP*GP*UP*AP*CP*AP*UP*C)-3' |
|  |  | 2yjy_C: 5'-R(*AP*UP*UP*GP*CP*AP*UP*AP*UP*AP)-3' |
|  |  | 2yjy_D: 5'-R(*AP*UP*UP*GP*CP*AP*UP*AP*UP*AP)-3' |
|  |  | 3q0q_B: 5'-R(UP*GP*UP*AP*AP*AP*UP*A)-3' |
|  |  | 3a6p_D: pre-microRNA |
|  |  | 3a6p_E: pre-microRNA |
|  |  | 3a6p_I: pre-microRNA |
|  |  | 3a6p_J: pre-microRNA |
|  |  | 3icq_E: RNA (62-MER) |
|  | | |
| 57836 | Ribosomal protein L44e | 1ffk_0: 23S RRNA |
|  |  | 1ffk_9: 5S RRNA |
|  |  | 3j0o_2: 60S ribosomal RNA fragment |
|  |  | 3j0o_5: 60S ribosomal RNA fragment |
|  |  | 3j0o_V: tRNA |
|  |  | 3j0o_W: tRNA |
|  |  | 3j0o_Y: tRNA |
|  | | |
| 191323 | automated matches | 3fht_C: RNA (5'-R(*UP*UP*UP*UP*UP*UP*UP*UP*UP*U)-3') |
|  |  | 3fht_D: RNA (5'-R(*UP*UP*UP*UP*UP*UP*UP*UP*UP*U)-3') |
|  |  | 3g0h_E: 5'-R(P*UP*UP*UP*UP*UP*UP*U)-3' |
|  |  | 3pew_B: RNA (5'-R(P*UP*UP*UP*UP*UP*U)-3') |
|  |  | 3pey_B: RNA (5'-R(P*UP*UP*UP*UP*UP*U)-3') |
|  |  | 2jly_C: 5'-R(*AP*GP*AP*CP*UP*AP*AP*CP*AP*AP*CP*U)-3' |
|  |  | 2jly_D: 5'-R(*AP*GP*AP*CP*UP*AP*AP*CP*AP*AP*CP*U)-3' |
|  |  | 2jlx_C: 5'-R(*AP*GP*AP*CP*UP*AP*AP*CP*AP*AP*CP*U)-3' |
|  |  | 2jlx_D: 5'-R(*AP*GP*AP*CP*UP*AP*AP*CP*AP*AP*CP*U)-3' |
|  |  | 4a36_R: 5'-R(*GP*CP*AP*UP*GP*CP*GP*AP*CP*CP*UP*CP*UP*GP *UP*UP*UP*GP*A)-3' |
|  |  | 4a36_S: 5'-R(*UP*CP*AP*AP*AP*CP*AP*GP*AP*GP*GP*UP*CP*GP *CP*AP*UP*GP*C)-3' |
|  |  | 4a36_T: 5'-R(*GP*CP*AP*UP*GP*CP*GP*AP*CP*CP*UP*CP*UP*GP *UP*UP*UP*GP*A)-3' |
|  |  | 4a36_U: 5'-R(*UP*CP*AP*AP*AP*CP*AP*GP*AP*GP*GP*UP*CP*GP *CP*AP*UP*GP*C)-3' |
|  |  | 2xgj_C: RNA (5'-(*AP*AP*AP*AP*A)-3') |
|  |  | 3o8r_C: RNA (5'-R(P*UP*(5BU)P*UP*UP*UP*U)-3') |
|  | | |
| 58323 | 30S ribosomal protein THX | 1xmo_A: 16S ribosomal RNA |
|  | | |
| 54815 | Prokaryotic type KH domain (KH-domain type II) | 1x1l_A: RNA (130-MER) |
|  |  | 2atw_B: ribosomal RNA (5'- AGAACUCAAUAG -3') |
|  |  | 2atw_D: ribosomal RNA (5'- AGAACUCAAUAG -3') |
|  | | |
| 118397 | Ilarvirus coat protein N-terminal fragment | 1xok_A: alfalfa mosaic virus RNA 3' UTR |
|  |  | 1xok_B: alfalfa mosaic virus RNA 3' UTR |
|  | | |
| 103693 | Signal recognition particle (SRP) complex | 1ry1_A: ALU DOMAIN (SRP9, SRP14 + RNA) |
|  |  | 1ry1_E: ALU DOMAIN (RNA FRAGMENTS) |
|  |  | 1ry1_M: fragment of 7S RNA |
|  |  | 1ry1_N: RNA (31-MER) |
|  |  | 4uyj_R: SRP RNA |
|  |  | 4uyj_S: SRP RNA |
|  | | |
| 57749 | Ribosomal protein L24e | 4adx_0: 23S RRNA |
|  | | |
| 54193 | L15e | 1ffk_0: 23S RRNA |
|  |  | 1ffk_9: 5S RRNA |
|  |  | 1yi2_0: 23S Ribosomal RNA |
|  |  | 1yi2_9: 5S Ribosomal RNA |
|  |  | 2qa4_0: 23S RIBOSOMAL RNA |
|  |  | 2qa4_9: 5S RIBOSOMAL RNA |
|  |  | 1jj2_0: 23S RRNA |
|  |  | 1jj2_9: 5S RRNA |
|  |  | 3ccl_0: 23S RIBOSOMAL RNA |
|  |  | 3ccl_9: 5S RIBOSOMAL RNA |
|  | | |
| 143035 | Ribosomal protein L35p | 2zjp_X: RIBOSOMAL 23S RNA |
|  |  | 2zjp_Z: RIBOSOMAL 5S RNA |
|  |  | 2rdo_B: 23S RIBOSOMAL RNA |
|  | | |
| 53960 | C-type lysozyme | 4m4o_B: RNA (59-MER) |
|  | | |
| 50461 | Ribosomal protein L3 | 1xbp_0: 23S RIBOSOMAL RNA |
|  |  | 1xbp_9: 5S RIBOSOMAL RNA |
|  |  | 2rdo_B: 23S RIBOSOMAL RNA |
|  |  | 1ffk_0: 23S RRNA |
|  |  | 1ffk_9: 5S RRNA |
|  |  | 3cma_0: 23S RIBOSOMAL RNA |
|  |  | 3cma_9: 5S RIBOSOMAL RNA |
|  |  | 4wwt_1: 23S RRNA (2899-MER) |
|  |  | 1ml5_a: 50S 23S RIBOSOMAL RNA |
|  |  | 3j3w_A: ribosome RNA 23S |
|  | | |
| 110640 | PIWI domain | 2bgg_P: 5'-R(*UP*UP*CP*GP*AP*CP*GP*CP)-3' |
|  |  | 2bgg_R: 5'-R(*UP*UP*CP*GP*AP*CP*GP*CP)-3' |
|  |  | 2bgg_S: 5'-R(*GP*UP*CP*GP*AP*AP*UP*UP)-3' |
|  |  | 2f8s_C: 5'-R(P*AP*GP*AP*CP*AP*GP*CP*AP*UP*AP*UP*AP*UP*GP*CP*UP*GP*UP*CP*UP*UP*U)-3' |
|  |  | 2f8s_D: 5'-R(P*AP*GP*AP*CP*AP*GP*CP*AP*UP*AP*UP*AP*UP*GP*CP*UP*GP*UP*CP*UP*UP*U)-3' |
|  |  | 4f1n_F: RNA 5'-R(P*UP*AP*AP*AP*AP*AP*AP*AP*A)-3' |
|  | | |
| 50013 | EV matrix protein | 1h2d_R: 5'-R(*UP*GP*AP)-3' |
|  | | |
| 88636 | Comoviridae-like VP | 1bmv_M: RNA (5'-R(*GP*GP*UP*CP*AP*AP*AP*AP*UP*GP*C)-3 |
|  | | |
| 46853 | Z-DNA binding domain | 2gxb_E: 5'-R(P*(DU)P*CP*GP*CP*GP*CP*G)-3' |
|  |  | 4uer_A: 18S RRNA |
|  |  | 3j81_2: 18S rRNA |
|  |  | 3j81_3: mRNA |
|  |  | 4kzz_i: 18S Ribosomal RNA |
|  |  | 4kzz_j: initiator Met-RNA-i |
|  |  | 4kzz_k: mRNA |
|  |  | 4ejt_G: 5'-R(*AP*A)-3' |
|  |  | 3j7a_A: 18S ribosomal RNA |
|  |  | 4d61_1: 18S RRNA |
|  |  | 4d61_j: CRICKET PARALYSIS VIRUS IRES RNA |
|  | | |
| 110325 | Ribosomal L27 protein | 2rdo_B: 23S RIBOSOMAL RNA |
|  |  | 1xbp_0: 23S RIBOSOMAL RNA |
|  |  | 1xbp_9: 5S RIBOSOMAL RNA |
|  | | |
| 89818 | Mago nashi protein | 2j0q_E: 5'-R(*UP*UP*UP*UP*UP*UP*UP*UP*UP*UP*UP*UP*UP*UP*U)-3' |
|  |  | 2j0q_H: 5'-R(*UP*UP*UP*UP*UP*UP*UP*UP*UP*UP*UP*UP*UP*UP*U)-3' |
|  | | |
| 117131 | CsrA-like | 2jpp_C: RNA (5'- R(*GP*GP*GP*CP*UP*UP*CP*AP*CP*GP*GP*AP*UP*GP*AP*AP*GP*CP*CP *C)-3') |
|  |  | 2jpp_D: RNA (5'- R(*GP*GP*GP*CP*UP*UP*CP*AP*CP*GP*GP*AP*UP*GP*AP*AP*GP*CP*CP *C)-3') |
|  | | |
| 55191 | Arginyl-tRNA synthetase (ArgRS), N-terminal 'additional' domain | 1f7u_B: TRNA(ARG) |
|  |  | 2zue_B: tRNA-Arg |
|  | | |
| 58177 | Reovirus components | 1uon_B: 5'-R(*GP*GP*GP*GP*GP*)-3' |
|  |  | 1uon_C: 5'-R(*UP*AP*GP*CP*CP*CP*CP*CP*)-3' |
|  |  | 2r7r_X: RNA (5'-R(*UP*GP*UP*GP*AP*CP*C)-3') |
|  | | |
| 101691 | PAZ domain | 3mj0_B: RNA (5'-R(*CP*GP*UP*UP*AP*CP*GP*CP*(OMU))-3') |
|  |  | 1si3_B: 5'-R(*CP*GP*UP*GP*AP*CP*UP*CP*U)-3' |
|  |  | 1t2r_B: 5'-R(*CP*UP*CP*AP*C)-3' |
|  |  | 4kxt_B: RNA |
|  |  | 3o7v_A: RNA (5'-R(*GP*CP*GP*AP*AP*UP*AP*UP*UP*CP*GP*CP*UP*(OMU))- 3') |
|  |  | 4w5r_B: RNA (5'-R(P*UP*UP*CP*AP*CP*AP*UP*UP*GP*UP*U)-3') |
|  |  | 4w5r_D: RNA (5'-R(P*AP*AP*UP*GP*UP*GP*AP*AP*A)-3') |
|  |  | 4f3t_R: RNA (5'- R(P*UP*AP*AP*AP*GP*UP*GP*CP*UP*UP*AP*UP*AP*GP*UP*G*CP*AP*GP*G)-3') |
|  |  | 4krf_R: RNA (5'- R(P*UP*GP*AP*GP*GP*UP*AP*GP*UP*AP*GP*GP*UP*UP*GP*UP*AP*UP*AP*GP*UP*U)-3') |
|  |  | 3o6e_A: RNA (5'-R(*GP*CP*GP*AP*AP*UP*AP*UP*UP*CP*GP*CP*UP*(OMU))- 3') |
|  | | |
| 64660 | Ribosomal protein L10 | 1jj2_0: 23S RRNA |
|  |  | 1jj2_9: 5S RRNA |
|  |  | 3cpw_0: 23S RIBOSOMAL RNA |
|  |  | 3cpw_9: 5S RIBOSOMAL RNA |
|  | | |
| 46993 | Ribosomal protein S20 | 1fjg_A: 16S RIBOSOMAL RNA |
|  |  | 1xmo_A: 16S ribosomal RNA |
|  |  | 4adv_A: 16S RIBOSOMAL RNA |
|  | | |
| 144200 | Ribosomal protein L32p | 2zjp_X: RIBOSOMAL 23S RNA |
|  |  | 3j7z_A: 23S rRNA |
|  |  | 4wwt_1: 23S RRNA (2899-MER) |
|  | | |
| 160958 | Poxvirus poly(A) polymerase catalytic subunit-like | 3er8_F: RNA/DNA chimera 5'-R(P*UP*UP*)D(C)-3' |
|  |  | 3er8_H: RNA/DNA chimera 5'-D(P*CP*)R(UP*U)-3' |
|  | | |
| 191538 | automated matches | 3ahu_X: 5'-R(*AP*GP*AP*GP*AP*G)-3' |
|  |  | 4nl3_R: 5'-R(*UP*UP*UP*UP*UP*U)-3' |
|  |  | 4nl3_Z: 5'-R(*UP*UP*UP*UP*UP*U)-3' |
|  | | |
| 102937 | 2'-5'-oligoadenylate synthetase 1, OAS1, N-terminal domain | 4ig8_B: RNA (5'- R(*GP*GP*CP*UP*UP*UP*UP*GP*AP*CP*CP*UP*UP*UP*AP*UP*GP*C)-3') |
|  |  | 4ig8_C: RNA (5'- R(*GP*CP*AP*UP*AP*AP*AP*GP*GP*UP*CP*AP*AP*AP*AP*GP*CP*C)-3') |
|  |  | 4s3n_B: RNA (5'- R(*GP*GP*CP*UP*UP*UP*UP*GP*AP*CP*CP*UP*UP*UP*AP*UP*GP*AP*A)-3') |
|  |  | 4s3n_C: RNA (5'- R(*GP*GP*CP*UP*UP*UP*UP*GP*AP*CP*CP*UP*UP*UP*AP*UP*GP*AP*A)-3') |
|  | | |
| 55667 | Ribonuclease PH domain 2-like | 2jea_C: RNA |
|  |  | 3m7n_Y: 5'-R(*C*UP*CP*CP*CP*C)-3' |
|  |  | 3m7n_Z: 5'-R(*C*UP*CP*CP*CP*C)-3' |
|  |  | 3m85_Y: 5'-R(*CP*UP*CP*CP*CP*C)-3' |
|  |  | 3m85_Z: 5'-R(*CP*UP*CP*CP*CP*C)-3' |
|  |  | 4am3_E: RNA, 5'-R(*UP*AP*AP*CP*UP*UP*UP*GP*GP)-3' |
|  |  | 4am3_H: RNA, 5'-R(*UP*AP*AP*CP*UP*UP*UP*GP*GP)-3' |
|  |  | 4am3_I: RNA, 5'-R(*UP*AP*AP*CP*UP*UP*UP*GP*GP)-3' |
|  |  | 4ifd_R: RNA (45-MER) |
|  | | |
| 47196 | TMV-like viral coat proteins | 3j06_R: 5'-R(P*AP*UP*G)-3' |
|  |  | 1cgm_I: RNA (5'-R(P*GP*AP*A)-3') |
|  |  | 1vtm_R: RNA (5'-R(P*GP*AP*A)-3') |
|  |  | 1rmv_B: RIBGRASS MOSAIC VIRUS RNA |
|  |  | 3pdm_R: RNA (5'-R(P*GP*AP*A)-3') |
|  | | |
| 48141 | Ribosomal protein L19 (L19e) | 1ffk_0: 23S RRNA |
|  |  | 1ffk_9: 5S RRNA |
|  |  | 3cma_0: 23S RIBOSOMAL RNA |
|  |  | 3cma_6: RNA (5'-R(*CP*CP*(8AN))-3') |
|  |  | 3cma_9: 5S RIBOSOMAL RNA |
|  |  | 2wwb_F: 25S RRNA |
|  |  | 2wwb_G: 25S RRNA |
|  | | |
| 88651 | Satellite viruses | 4nia_6: RNA (5'-R(P*UP*U)-3') |
|  |  | 4nia_b: RNA (5'-R(P*AP*AP*AP*AP*AP*AP*AP*AP*AP*A)-3') |
|  |  | 4nia_c: RNA (5'-R(P*AP*AP*AP*AP*AP*AP*AP*AP*AP*A)-3') |
|  |  | 4nia_q: RNA (5'-R(P*UP*UP*UP*UP*UP*UP*UP*UP*UP*U)-3') |
|  | | |
| 55000 | Ribosomal protein S10 | 1fjg_A: 16S RIBOSOMAL RNA |
|  |  | 1fjg_X: FRAGMENT OF MESSENGER RNA |
|  |  | 5aj3_A: MITORIBOSOMAL 12S RRNA |
|  |  | 3r2d_R: 5'-R(*GP*GP*CP*UP*CP*CP*UP*UP*GP*GP*CP*A)-3' |
|  |  | 3r2d_S: 5'-R(*GP*GP*CP*UP*CP*CP*UP*UP*GP*GP*CP*A)-3' |
|  |  | 4uer_A: 18S RRNA |
|  |  | 4kzz_i: 18S Ribosomal RNA |
|  |  | 4kzz_k: mRNA |
|  |  | 3j7a_A: 18S ribosomal RNA |
|  |  | 3j80_2: 18S rRNA |
|  |  | 4adv_A: 16S RIBOSOMAL RNA |
|  | | |
| 53138 | Ribosomal protein L18 and S11 | 2rdo_A: 5S RIBOSOMAL RNA |
|  |  | 2zjp_X: RIBOSOMAL 23S RNA |
|  |  | 2zjp_Z: RIBOSOMAL 5S RNA |
|  |  | 1fjg_A: 16S RIBOSOMAL RNA |
|  |  | 1fjg_X: FRAGMENT OF MESSENGER RNA |
|  |  | 1s72_0: 23S ribosomal RNA |
|  |  | 1s72_9: 5S ribosomal RNA |
|  |  | 3j80_2: 18S rRNA |
|  |  | 3j0l_1: 60S ribosomal RNA fragment |
|  |  | 3j0l_2: 60S ribosomal RNA fragment |
|  |  | 3j0l_b: 40S ribosomal RNA fragment |
|  |  | 3j0l_c: 40S ribosomal RNA fragment |
|  |  | 3j0l_h: 40S ribosomal RNA fragment |
|  |  | 3j0l_V: tRNA |
|  |  | 3j0l_W: tRNA |
|  |  | 3j0l_Y: tRNA |
|  |  | 5aj3_A: MITORIBOSOMAL 12S RRNA |
|  |  | 4wwt_1: 23S RRNA (2899-MER) |
|  |  | 4wwt_s: THERMUS THERMOPHILUS HB8 GENOMIC DNA, COMPLETE GENOME |
|  |  | 4kzy_i: 18S Ribosomal RNA |
|  |  | 4uer_A: 18S RRNA |
|  |  | 4adv_A: 16S RIBOSOMAL RNA |
|  | | |
| 55283 | Ribosomal protein L5 | 1xbp_0: 23S RIBOSOMAL RNA |
|  |  | 1xbp_9: 5S RIBOSOMAL RNA |
|  |  | 1s72_0: 23S ribosomal RNA |
|  |  | 1s72_9: 5S ribosomal RNA |
|  |  | 1mji_C: 5S rRNA fragment |
|  |  | 1mji_D: 5S rRNA fragment |
|  |  | 3j8g_A: 5S rRNA |
|  |  | 3j8g_B: 23S rRNA |
|  |  | 3j3v_A: ribosome RNA 23S |
|  |  | 3j3v_B: ribosome RNA 5S |
|  |  | 2noq_A: CrPV IRES |
|  |  | 2noq_E: 25S ribosomal RNA |
|  | | |
| 52724 | RNA helicase | 2jlw_C: 5'-R(*UP*AP*GP*AP*CP*UP*AP*AP*CP*AP*AP*CP*U)-3' |
|  |  | 2jlw_D: 5'-R(*UP*AP*GP*AP*CP*UP*AP*AP*CP*AP*AP*CP*U)-3' |
|  |  | 3o8r_C: RNA (5'-R(P*UP*(5BU)P*UP*UP*UP*U)-3') |
|  | | |
| 46562 | Ribosomal protein L29 (L29p) | 2rdo_B: 23S RIBOSOMAL RNA |
|  |  | 1xbp_0: 23S RIBOSOMAL RNA |
|  |  | 1s72_0: 23S ribosomal RNA |
|  |  | 1s72_9: 5S ribosomal RNA |
|  |  | 4wwt_1: 23S RRNA (2899-MER) |
|  |  | 2go5_A: SRP RNA |
|  | | |
| 191470 | automated matches | 4q9r_R: Spinach RNA aptamer |
|  |  | 4kzd_R: RNA (84-MER) |
|  |  | 4q9q_R: Spinach RNA aptamer |
|  |  | 2r8s_R: P4-P6 RNA RIBOZYME DOMAIN |
|  | | |
| 74939 | Pleiotropic translational regulator Hfq | 3qsu_P: RNA (5'-R(P*AP*AP*AP*AP*AP*AP*A)-3') |
|  |  | 4v2s_Q: RYDC |
|  |  | 4m7d_P: U6 snRNA |
|  | | |
| 52043 | Ribosomal protein L32e | 1ffk_0: 23S RRNA |
|  |  | 1s72_0: 23S ribosomal RNA |
|  |  | 1s72_9: 5S ribosomal RNA |
|  | | |
| 111065 | Hut operon positive regulatory protein HutP | 1wmq_C: 5'-R(P*UP*UP*UP*AP*GP*UP*U)-3' |
|  | | |
| 81310 | Ribotoxin | 1jbr_C: 5'- R(*GP*CP*GP*CP*UP*CP*CP*UP*CP*AP*GP*UP*AP*CP*GP*AP*GP*(A23) )-3' |
|  |  | 1jbr_F: 5'-R(*GP*GP*AP*AP*CP*CP*GP*GP*AP*GP*CP*GP*C)-3' |
|  | | |
| 88803 | Archaeosine tRNA-guanine transglycosylase, C2 domain | 1j2b_C: tRNA(Val) |
|  |  | 1j2b_D: tRNA(Val) |
|  | | |
| 46925 | RNA polymerase subunit RPB10 | 2b63_R: 31-MER |
|  | | |
| 47447 | Signal peptide-binding domain | 1dul_B: 4.5 S RNA DOMAIN IV |
|  |  | 1mfq_A: 7S RNA of human SRP |
|  |  | 3ndb_M: SRP RNA |
|  |  | 5aka_7: 4.5 S RNA |
|  |  | 5aka_B: 23S RNA |
|  |  | 2v3c_N: 7S RNA |
|  |  | 2xxa_F: 4.5S RNA |
|  |  | 2xxa_G: 4.5S RNA |
|  |  | 3zn8_G: 4.5 S RNA |
|  | | |
| 143804 | Ribosomal protein L31p | 2j28_A: 5S RIBOSOMAL RNA |
|  |  | 2j28_B: 23S RIBOSOMAL RNA |
|  |  | 3j7z_A: 23S rRNA |
|  |  | 3j7z_B: 5S rRNA |
|  | | |
| 69746 | Pseudouridine synthase II TruB | 1ze2_C: 5'-R(*GP*GP*CP*CP*AP*CP*GP*GP*UP*(FHU) P*CP*GP*AP*AP*UP*CP*CP*GP*UP*GP*GP*C)-3' |
|  |  | 1ze2_D: 5'-R(*GP*GP*CP*CP*AP*CP*GP*GP*UP*(FHU) P*CP*GP*AP*AP*UP*CP*CP*GP*UP*GP*GP*C)-3' |
|  |  | 1zl3_B: 5'-R(*GP*GP*CP*AP*AP*CP*GP*GP*UP*(FLO) UP*CP*GP*AP*UP*CP*CP*CP*GP*UP*UP*GP*C)-3' |
|  |  | 1k8w_B: 5'-R(*GP*GP*CP*AP*AP*CP*GP*GP*UP*(FHU) P*CP*GP*AP*UP*CP*CP*CP*GP*UP*UP*GP*C)-3' |
|  |  | 2ab4_B: 5'-R(*CP*CP*AP*CP*GP*GP*UP*(FHU) P*CP*GP*AP*AP*UP*CP*CP*GP*UP*GP*GP*C)-3' |
|  |  | 1r3e_C: 5'-R(*CP*UP*GP*UP*GP*UP*(FHU) P*CP*GP*AP*UP*CP*CP*AP*CP*AP*G)-3' |
|  |  | 1r3e_D: 5'-R(*CP*UP*GP*UP*GP*UP*UP*CP*GP*AP*UP*CP*CP*AP*CP*AP*G)- 3' |
|  |  | 1r3e_E: 5'-R(*CP*UP*GP*UP*GP*UP*UP*CP*GP*AP*UP*CP*CP*AP*CP*AP*G)- 3' |
|  |  | 2hvy_E: H/ACA RNA |
|  |  | 2rfk_D: guide RNA 1 |
|  |  | 2rfk_E: guide RNA 2 |
|  |  | 2rfk_F: target RNA |
|  | | |
| 54822 | Ribosomal protein S3 C-terminal domain | 1fjg_A: 16S RIBOSOMAL RNA |
|  |  | 1fjg_X: FRAGMENT OF MESSENGER RNA |
|  |  | 5aj3_A: MITORIBOSOMAL 12S RRNA |
|  |  | 4uer_A: 18S RRNA |
|  | | |
| 54792 | Eukaryotic type KH-domain (KH-domain type I) | 2py9_E: 12-mer C-rich strand of human telomeric RNA |
|  |  | 1ec6_C: 20-MER RNA HAIRPIN |
|  |  | 1ec6_D: 20-MER RNA HAIRPIN |
|  |  | 1k1g_B: 5'-R(*UP*AP*UP*AP*CP*UP*AP*AP*CP*AP*A)-3' |
|  |  | 4jvy_D: RNA (5'-R(P*CP*UP*AP*AP*CP*AP*A)-3') |
|  |  | 4jvy_F: RNA (5'-R(P*CP*UP*AP*AP*CP*AP*A)-3') |
|  |  | 4wan_B: RNA (5'-R(P*UP*AP*CP*UP*AP*AP*CP*A)-3') |
|  |  | 4wan_D: RNA (5'-R(P*UP*AP*CP*UP*AP*AP*CP*A)-3') |
|  |  | 4wan_F: RNA (5'-R(P*UP*AP*CP*UP*AP*AP*CP*A)-3') |
|  |  | 4wan_H: RNA (5'-R(P*UP*AP*CP*UP*AP*AP*CP*A)-3') |
|  |  | 4fsj_R: Random cellular RNA fragments |
|  |  | 4wal_B: RNA (5'-R(P*UP*AP*CP*UP*AP*AP*CP*A)-3') |
|  |  | 4jvh_D: RNA (5'-R(*UP*UP*CP*AP*CP*UP*AP*AP*CP*AP*A)-3 |
|  |  | 4b8t_B: 5'-R(*AP*GP*GP*GP*UP)-3' |
|  | | |
| 55195 | Ribosome recycling factor, RRF | 1zn0_C: 16S RIBOSOMAL RNA |
|  |  | 4pmi_A: Rev-Response-Element RNA |
|  |  | 1y69_0: 23S ribosomal RNA |
|  |  | 1y69_9: 5S ribosomal RNA |
|  | | |
| 143801 | Ribosomal protein L28 | 2zjp_X: RIBOSOMAL 23S RNA |
|  |  | 2zjp_Z: RIBOSOMAL 5S RNA |
|  | | |
| 58182 | RNA polymerase | 2r93_R: RNA (5'- R(*UP*GP*AP*UP*UP*CP*UP*CP*UP*AP*UP*CP*GP*GP*AP*AP*UP*C)- 3') |
|  |  | 2r92_T: RNA (5'- R(*CP*UP*UP*GP*AP*CP*GP*CP*CP*UP*GP*GP*UP*CP*AP*AP*A)-3') |
|  |  | 2r92_P: RNA (5'- R(*UP*GP*CP*AP*UP*AP*AP*AP*GP*AP*CP*CP*AP*GP*GP*C)-3') |
|  | | |
| 47064 | Ribosomal protein S15 | 1f7y_B: 16S RIBOSOMAL RNA FRAGMENT |
|  |  | 1kuq_B: 16S RIBOSOMAL RNA FRAGMENT |
|  |  | 1fjg_A: 16S RIBOSOMAL RNA |
|  |  | 1g1x_D: 16S RIBOSOMAL RNA |
|  |  | 1g1x_E: 16S RIBOSOMAL RNA |
|  |  | 5aj3_A: MITORIBOSOMAL 12S RRNA |
|  | | |
| 64480 | dsRNA phage RNA-dependent RNA-polymerase | 1uvi_D: 5'-R(*UP*UP*UP*UP*CP*CP)-3' |
|  | | |
| 231597 | automated matches | 2y9h_J: 5'-R(*UP*CP*CP*CP*CP*AP*CP*GP*CP*GP*UP*GP*UP*GP *GP*GP*DGP*AP*UP)-3' |
|  |  | 2y9h_L: 5'-R(*UP*CP*CP*CP*CP*AP*CP*GP*CP*GP*UP*GP*UP*GP *GP*GP*DGP*AP*UP)-3' |
|  |  | 2y9h_N: 5'-R(*UP*CP*CP*CP*CP*AP*CP*GP*CP*GP*UP*GP*UP*GP *GP*GP*DGP*AP*UP)-3' |
|  |  | 2y9h_P: 5'-R(*UP*CP*CP*CP*CP*AP*CP*GP*CP*GP*UP*GP*UP*GP *GP*GP*DGP*AP*UP)-3' |
|  |  | 4u7u_L: crRNA |
|  |  | 3qrp_B: RNA (5'-R(*GP*UP*CP*CP*CP*CP*AP*CP*(PGP))-3') |
|  |  | 3qrp_C: RNA (5'-R(P*(U5P)P*GP*UP*GP*GP*GP*G)-3') |
|  | | |
| 141245 | Ribosomal protein L19 | 1xbp_0: 23S RIBOSOMAL RNA |
|  |  | 1xbp_9: 5S RIBOSOMAL RNA |
|  |  | 3j8g_A: 5S rRNA |
|  |  | 3j8g_B: 23S rRNA |
|  | | |
| 55130 | Ribosomal protein L30p/L7e | 1xbp_0: 23S RIBOSOMAL RNA |
|  |  | 1xbp_9: 5S RIBOSOMAL RNA |
|  |  | 2rdo_B: 23S RIBOSOMAL RNA |
|  |  | 1ffk_0: 23S RRNA |
|  |  | 1ffk_9: 5S RRNA |
|  |  | 3j3v_A: ribosome RNA 23S |
|  |  | 3j3v_B: ribosome RNA 5S |
|  | | |
| 64485 | RNA-polymerase beta | 2b63_R: 31-MER |
|  | | |
| 140507 | FHV B2 protein-like | 2az0_C: 5'-R(*GP*CP*AP*(5BU)P*GP*GP*AP*CP*GP*CP*GP*(5BU) P*CP*CP*AP*(5BU)P*GP*C)-3' |
|  |  | 2az0_D: 5'-R(*GP*CP*AP*(5BU)P*GP*GP*AP*CP*GP*CP*GP*(5BU) P*CP*CP*AP*(5BU)P*GP*C)-3' |
|  | | |
| 46947 | Ribosomal protein S13 | 1n32_A: 16S RIBOSOMAL RNA |
|  |  | 4adv_A: 16S RIBOSOMAL RNA |
|  | | |
| 54190 | L23p | 2aar_0: 23S ribosomal RNA |
|  |  | 3cma_0: 23S RIBOSOMAL RNA |
|  |  | 3cma_9: 5S RIBOSOMAL RNA |
|  |  | 3bbx_B: 23S ribosomal RNA |
|  |  | 2go5_A: SRP RNA |
|  |  | 4wwt_1: 23S RRNA (2899-MER) |
|  |  | 3j3w_A: ribosome RNA 23S |
|  |  | 2wwb_D: 5.8S RRNA |
|  |  | 2wwb_E: 25S RRNA |
|  |  | 2wwb_F: 25S RRNA |
|  | | |
| 51220 | Trp RNA-binding attenuation protein (TRAP) | 1c9s_W: SINGLE STRANDED RNA (55-MER) |
|  | | |
| 57757 | Retrovirus zinc finger-like domains | 1f6u_B: HIV-1 STEM-LOOP SL2 FROM PSI-RNA PACKAGING |
|  |  | 1u6p_B: 101-MER |
|  | | |
| 144203 | Ribosomal protein L33p | 1xbp_0: 23S RIBOSOMAL RNA |
|  |  | 1xbp_9: 5S RIBOSOMAL RNA |
|  |  | 2j28_B: 23S RIBOSOMAL RNA |
|  | | |
| 46912 | Ribosomal protein S18 | 1g1x_D: 16S RIBOSOMAL RNA |
|  |  | 1g1x_E: 16S RIBOSOMAL RNA |
|  |  | 1g1x_I: 16S RIBOSOMAL RNA |
|  |  | 2uxb_A: 16S RIBOSOMAL RNA |
|  |  | 4adv_A: 16S RIBOSOMAL RNA |
|  | | |
| 47061 | N-terminal, RNA-binding domain of nonstructural protein NS1 | 2zko_C: RNA (5'- R(P*AP*GP*AP*CP*AP*GP*CP*AP*UP*UP*AP*UP*GP*CP*UP*GP*UP*CP*UP*UP*U)- 3') |
|  |  | 2zko_D: RNA (5'- R(P*AP*GP*AP*CP*AP*GP*CP*AP*UP*UP*AP*UP*GP*CP*UP*GP*UP*CP*UP*UP*U)- 3') |
|  | | |
| 227190 | automated matches | 3rtj_D: RNA (5'-R(*AP*G)-3') |
|  | | |
| 54212 | Translational machinery components | 1i95_A: 16S RRNA |
|  |  | 2uua_A: 16S RRNA |
|  |  | 4d61_1: 18S RRNA |
|  |  | 4d61_j: CRICKET PARALYSIS VIRUS IRES RNA |
|  |  | 3j80_2: 18S rRNA |
|  |  | 4adv_A: 16S RIBOSOMAL RNA |
|  |  | 3j7a_A: 18S ribosomal RNA |
|  |  | 4uer_A: 18S RRNA |
|  |  | 5aj3_A: MITORIBOSOMAL 12S RRNA |
|  | | |
| 58340 | Box B RNA-binding N peptide | 1a4t_A: BOXB RNA |
|  |  | 1nyb_B: BoxB RNA |
|  |  | 1qfq_A: 15-mer nutRboxB RNA hairpin |
|  | | |
| 64264 | Prokaryotic ribosomal protein L17 | 1xbp_0: 23S RIBOSOMAL RNA |
|  |  | 2rdo_B: 23S RIBOSOMAL RNA |
|  |  | 4wwe_1: 23S RRNA (2899-MER) |
|  |  | 3j3w_A: ribosome RNA 23S |
|  | | |
| 53342 | Fibrillarin homologue | 3nmu_D: RNA (34-MER) |
|  |  | 3pla_G: C/D guide RNA |
|  |  | 3pla_H: C/D guide RNA |
|  |  | 3pla_I: RNA (5'-R(*CP*CP*AP*UP*GP*AP*GP*UP*GP*U)-3') |
|  | | |
| 69696 | SRP19 | 1l9a_B: signal recognition particle RNA S domain |
|  |  | 1lng_B: 7S.S SRP RNA |
|  |  | 2j37_A: SRP RNA |
|  |  | 1jid_B: HELIX 6 OF HUMAN SRP RNA |
|  |  | 3ktw_C: SRP RNA |
|  |  | 3ktw_D: SRP RNA |
|  | | |
| 75465 | Tyrosyl-tRNA synthetase (TyrRS), C-terminal domain | 1h3e_B: WILD-TYPE TRNATYR(GUA) |
|  | | |
| 54571 | Ribosomal protein S19 | 1hnw_A: 16S RIBOSOMAL RNA |
|  |  | 4uer_A: 18S RRNA |
|  | | |
| 144211 | Nucleolar RNA-binding protein Nop10-like | 3lwq_D: H/ACA RNA |
|  |  | 2hvy_E: H/ACA RNA |
|  | | |
| 54844 | Ribosomal protein L22 | 2rdo_B: 23S RIBOSOMAL RNA |
|  |  | 1xbp_0: 23S RIBOSOMAL RNA |
|  |  | 1yj9_0: 23S Ribosomal RNA |
|  |  | 1yj9_9: 5S Ribosomal RNA |
|  |  | 3cma_0: 23S RIBOSOMAL RNA |
|  |  | 3cma_9: 5S RIBOSOMAL RNA |
|  |  | 2wwb_D: 5.8S RRNA |
|  |  | 2wwb_E: 25S RRNA |
|  |  | 2wwb_F: 25S RRNA |
|  |  | 2wwb_G: 25S RRNA |
|  |  | 3j3v_A: ribosome RNA 23S |
|  |  | 1ml5_a: 50S 23S RIBOSOMAL RNA |
|  |  | 1ml5_A: 30S 16S RIBOSOMAL RNA |
|  | | |
| 267610 | Filoviridae VP35 | 3ks8_E: 5'- R(*AP*GP*AP*AP*GP*GP*AP*GP*GP*GP*AP*GP*GP*GP*AP*GP*GP*A)- 3' |
|  |  | 3ks8_F: 5'- R(*UP*CP*CP*UP*CP*CP*CP*UP*CP*CP*CP*UP*CP*CP*UP*UP*CP*U)- 3' |
|  |  | 4ghl_E: short palindromic RNA AGACAGCAUAUGCUGUCU |
|  |  | 4ghl_F: short palindromic RNA AGACAGCAUAUGCUGUCU |
|  |  | 4lg2_E: dsRNA |
|  |  | 3l25_C: RNA (5'-R(*CP*GP*CP*AP*UP*GP*CP*G)-3') |
|  |  | 3l25_F: RNA (5'-R(*CP*GP*CP*AP*UP*GP*CP*G)-3') |
|  |  | 4gha_I: RNA (5'-R(*CP*UP*AP*GP*AP*CP*GP*UP*CP*UP*AP*G)-3') |
|  |  | 4gha_J: RNA (5'-R(*CP*UP*AP*GP*AP*CP*GP*UP*CP*UP*AP*G)-3') |
|  | | |
| 140810 | Rhabdovirus nucleocapsid protein | 2gtt_W: RNA (99-MER) |
|  |  | 2gtt_X: RNA (99-MER) |
|  |  | 2gic_R: 45-MER |
|  | | |
| 52068 | U2A'-like | 1a9n_Q: RNA (5'- R(*CP*CP*UP*GP*GP*UP*AP*UP*UP*GP*CP*AP*GP*UP*AP*CP*CP*UP*CP *CP*AP*GP* GP*U)-3') |
|  |  | 3ciy_C: 46-MER |
|  |  | 3ciy_D: 46-MER |
|  | | |
| 143043 | Guide RNA binding protein gBP | 2gje_R: guide RNA 40-mer |
|  |  | 2gje_S: RNA tetramer |
|  | | |
| 191377 | automated matches | 2dlc_Y: T-RNA (76-MER) |
|  |  | 2csx_C: RNA (75-MER) |
|  |  | 2csx_D: RNA (75-MER) |
|  |  | 2bte_B: TRNALEU TRANSCRIPT WITH ANTICODON CAG |
|  |  | 2bte_E: TRNALEU TRANSCRIPT WITH ANTICODON CAG |
|  |  | 4cqn_B: ESCHERICHIA COLI TRNA-LEU UAA ISOACCEPTOR |
|  |  | 4cqn_E: ESCHERICHIA COLI TRNA-LEU UAA ISOACCEPTOR |
|  |  | 1wz2_C: tRNA |
|  |  | 1wz2_D: tRNA |
|  | | |
| 88638 | Nodaviridae-like VP | 1f8v_R: RNA |
|  |  | 2bbv_N: RNA (5'-R(*UP*CP*UP*UP*AP*UP*AP*UP*CP*U)-3') |
|  |  | 4ftb_R: Flock House virus genomic RNA |
|  |  | 4fts_R: Random cellular RNAs |
|  |  | 4fte_R: Random cellular RNAs |
|  |  | 3lob_R: RNA (5'-R(*UP*UP*U*AP*UP*CP*UP*(P))-3') |
|  | | |
| 54687 | Ribosomal protein L10e | 1ffk_0: 23S RRNA |
|  |  | 1ffk_9: 5S RRNA |
|  |  | 1jj2_0: 23S RRNA |
|  |  | 1jj2_9: 5S RRNA |
|  |  | 2qex_0: 23S ribosomal RNA |
|  |  | 2qex_9: 5S ribosomal RNA |
|  |  | 3cma_0: 23S RIBOSOMAL RNA |
|  |  | 3cma_5: RNA (5'-R(*CP*CP*A)-3') |
|  |  | 3cma_9: 5S RIBOSOMAL RNA |
|  |  | 1ml5_a: 50S 23S RIBOSOMAL RNA |
|  |  | 1ml5_B: T-RNA(PHE) |
|  |  | 3j0l_1: 60S ribosomal RNA fragment |
|  |  | 3j0l_2: 60S ribosomal RNA fragment |
|  |  | 3j0l_3: 60S ribosomal RNA fragment |
|  |  | 3j0l_4: 60S ribosomal RNA fragment |
|  |  | 3j0l_6: 60S ribosomal RNA fragment |
|  |  | 3j0l_7: 60S ribosomal RNA fragment |
|  |  | 3j0l_a: 40S ribosomal RNA fragment |
|  |  | 3j0l_b: 40S ribosomal RNA fragment |
|  |  | 3j0l_h: 40S ribosomal RNA fragment |
|  |  | 3j0l_V: tRNA |
|  |  | 3j0l_W: tRNA |
|  |  | 3j0l_Y: tRNA |
|  | | |
| 54996 | Ribosomal protein S6 | 1i95_A: 16S RRNA |
|  |  | 4adv_A: 16S RIBOSOMAL RNA |
|  | | |
| 227299 | automated matches | 3lwr_D: H/ACA RNA |
|  |  | 3lwr_E: 5'-R(*GP*AP*GP*CP*GP*(4SU)P*GP*CP*GP*GP*UP*UP*U)-3' |
|  | | |
| 55659 | Ribosomal protein L9 N-domain | 2j28_B: 23S RIBOSOMAL RNA |
|  |  | 4wwt_1: 23S RRNA (2899-MER) |
|  | | |
| 54576 | Ribosomal protein L31e | 1s72_0: 23S ribosomal RNA |
|  |  | 1s72_9: 5S ribosomal RNA |
|  |  | 2j37_Z: RIBOSOMAL RNA |
|  | | |
| 89125 | Nop domain | 2ozb_C: RNA comprising the 5' Stem-Loop RNA of U4snR |
|  |  | 2ozb_F: RNA comprising the 5' Stem-Loop RNA of U4snR |
|  |  | 3siv_C: U4atac snRNA |
|  |  | 3siv_F: U4atac snRNA |
|  |  | 3siv_I: U4atac snRNA |
|  |  | 3siv_L: U4atac snRNA |
|  | | |
| 159704 | SepSecS-like | 3hl2_E: tRNASec |
|  | | |
| 81315 | Rel/Dorsal transcription factors, DNA-binding domain | 1ooa_C: RNA aptamer |
|  |  | 1ooa_D: RNA aptamer |
|  | | |
| 54769 | Double-stranded RNA-binding domain (dsRBD) | 1di2_C: RNA (5'-R(*GP*GP*CP*GP*CP*GP*CP*GP*CP*C)-3') |
|  |  | 1di2_D: RNA (5'-R(*GP*GP*CP*GP*CP*GP*CP*GP*CP*C)-3') |
|  |  | 1di2_E: RNA (5'-R(*GP*GP*CP*GP*CP*GP*CP*GP*CP*C)-3') |
|  |  | 3adl_B: RNA (5'-R(P*CP*GP*CP*GP*CP*GP*CP*GP*CP*G)-3') |
|  |  | 3adl_C: RNA (5'-R(P*CP*GP*CP*GP*CP*GP*CP*GP*CP*G)-3') |
|  |  | 1ekz_B: STAUFEN DOUBLE-STRANDED RNA BINDING DOMAIN |
|  |  | 1t4l_A: 5' terminal hairpin of snR47 precursor |
|  |  | 2nug_C: 5'-R(P*AP*AP*GP*GP*UP*CP*AP*UP*UP*CP*G)-3' |
|  |  | 2nug_D: 5'-R(P*AP*AP*GP*GP*UP*CP*AP*UP*UP*CP*G)-3' |
|  |  | 2nug_E: 5'-R(P*AP*GP*UP*GP*GP*CP*CP*UP*UP*GP*C)-3' |
|  |  | 2nug_F: 5'-R(P*AP*GP*UP*GP*GP*CP*CP*UP*UP*GP*C)-3' |
|  |  | 1yz9_C: 5'-R(*CP*GP*AP*AP*CP*UP*UP*CP*GP*CP*G)-3' |
|  |  | 1yz9_E: 5'-R(*CP*GP*AP*AP*CP*UP*UP*CP*GP*CP*G)-3' |
|  |  | 1yz9_F: 5'-R(*CP*GP*AP*AP*CP*UP*UP*CP*GP*CP*G)-3' |
|  |  | 2ez6_C: 28-MER |
|  |  | 2ez6_D: 28-MER |
|  |  | 1yyo_E: 5'-R(*CP*GP*CP*GP*AP*AP*UP*UP*CP*GP*CP*G)-3' |
|  |  | 1yyo_F: 5'-R(*CP*GP*CP*GP*AP*AP*UP*UP*CP*GP*CP*G)-3' |
|  |  | 3adi_D: RNA (5'-R(P*CP*UP*CP*GP*AP*UP*AP*AP*CP*C)-3') |
|  |  | 3adi_E: RNA (5'-R(*GP*GP*UP*UP*AP*UP*CP*GP*AP*G)-3') |
|  |  | 3htx_B: 5'- R(*GP*AP*UP*UP*UP*CP*UP*CP*UP*CP*UP*GP*CP*AP*AP*GP*CP*GP*AP *AP*AP*G)-3' |
|  |  | 3htx_C: 5'- R(P*UP*UP*CP*GP*CP*UP*UP*GP*CP*AP*GP*AP*GP*AP*GP*AP*AP*AP*U P*CP*AP*C)-3' |
|  |  | 3htx_E: 5'- R(*GP*AP*UP*UP*UP*CP*UP*CP*UP*CP*UP*GP*CP*AP*AP*GP*CP*GP*AP *AP*AP*G)-3' |
|  |  | 3htx_F: 5'- R(P*UP*UP*CP*GP*CP*UP*UP*GP*CP*AP*GP*AP*GP*AP*GP*AP*AP*AP*U P*CP*AP*C)-3' |
|  |  | 3vyy_C: RNA (5'-R(*GP*CP*GP*CP*GP*CP*GP*CP*GP*C)-3') |
|  |  | 3vyy_D: RNA (5'-R(*GP*CP*GP*CP*GP*CP*GP*CP*GP*C)-3') |
|  |  | 3vyy_E: RNA (5'-R(*GP*CP*GP*CP*GP*CP*GP*CP*GP*C)-3') |
|  |  | 3vyy_F: RNA (5'-R(*GP*CP*GP*CP*GP*CP*GP*CP*GP*C)-3') |
|  |  | 3j7y_A: 16S rRNA |
|  |  | 3vyx_B: RNA (5'-R(P*GP*CP*GP*CP*GP*CP*GP*CP*GP*C)-3') |
|  |  | 3vyx_C: RNA (5'-R(P*GP*CP*GP*CP*GP*CP*GP*CP*GP*C)-3') |
|  |  | 4oog_D: 34-mer RNA |
|  |  | 4ce4_A: 16S RRNA |
|  |  | 2l3j_B: RNA (71-MER) |
|  | | |
| 56686 | Reverse transcriptase | 1hvu_C: RNA (33 NUCLEOTIDE RNA PSEUDOKNOT) |
|  | | |
| 143927 | beta-CASP RNA-metabolising hydrolases | 3ie1_E: RNA (5'-R(P*UP*UP*UP*U)-3') |
|  |  | 3t3o_B: O2'methyl-RNA |
|  | | |
| 57784 | Transcriptional factor domain | 2b63_R: 31-MER |
|  | | |
| 52081 | Ribosomal proteins L15p and L18e | 1xbp_0: 23S RIBOSOMAL RNA |
|  |  | 2rdo_B: 23S RIBOSOMAL RNA |
|  |  | 1s72_0: 23S ribosomal RNA |
|  |  | 1s72_9: 5S ribosomal RNA |
|  | | |
| 50282 | Cold shock DNA-binding domain-like | 1i95_A: 16S RRNA |
|  |  | 1ffk_0: 23S RRNA |
|  |  | 1xbp_0: 23S RIBOSOMAL RNA |
|  |  | 1xbp_9: 5S RIBOSOMAL RNA |
|  |  | 3pf4_R: hexaribonucleotide (rGUCUUUA) |
|  |  | 1hr0_A: 16S RIBOSOMAL RNA |
|  |  | 1fjg_A: 16S RIBOSOMAL RNA |
|  |  | 2uu9_A: 16S RRNA |
|  |  | 2a8v_D: 5'-R(P*CP*CP*C)-3' |
|  |  | 3deg_A: A/L-tRNA |
|  |  | 1hnw_A: 16S RIBOSOMAL RNA |
|  |  | 2f4v_A: 16S ribosomal RNA |
|  |  | 1s72_0: 23S ribosomal RNA |
|  |  | 1s72_9: 5S ribosomal RNA |
|  |  | 3bbx_B: 23S ribosomal RNA |
|  |  | 4dr4_A: 16S rRNA |
|  |  | 4dr4_V: 5'-R(*UP*UP*U)-3' |
|  |  | 3trz_W: RNA (5'- R(*GP*GP*GP*CP*AP*GP*GP*GP*AP*UP*UP*UP*UP*GP*CP*CP*CP*GP*GP*AP*G)- 3') |
|  |  | 3trz_X: RNA (5'- R(*GP*GP*GP*CP*AP*GP*GP*GP*AP*UP*UP*UP*UP*GP*CP*CP*CP*GP*GP*AP*G)- 3') |
|  |  | 3trz_Y: RNA (5'- R(*GP*GP*GP*CP*AP*GP*GP*GP*AP*UP*UP*UP*UP*GP*CP*CP*CP*GP*GP*AP*G)- 3') |
|  |  | 3trz_Z: RNA (5'- R(*GP*GP*GP*CP*AP*GP*GP*GP*AP*UP*UP*UP*UP*GP*CP*CP*CP*GP*GP*AP*G)- 3') |
|  |  | 3j81_1: Met-tRNAi |
|  |  | 3j81_2: 18S rRNA |
|  |  | 3j81_3: mRNA |
|  |  | 3j7a_7: tRNA |
|  |  | 3j7a_A: 18S ribosomal RNA |
|  |  | 4qqb_C: msl2 mRNA |
|  |  | 4kzy_i: 18S Ribosomal RNA |
|  |  | 4oo1_S: POLY A RNA |
|  |  | 4adv_A: 16S RIBOSOMAL RNA |
|  |  | 4kzz_i: 18S Ribosomal RNA |
|  |  | 4kzz_j: initiator Met-RNA-i |
|  |  | 4kzz_k: mRNA |
|  |  | 4alp_E: HEXA URIDINE |
|  |  | 3iy9_A: LEISHMANIA TARENTOLAE MITOCHONDRIAL LARGE RIBOSOMAL SUBUNIT |
|  |  | 3j80_2: 18S rRNA |
|  |  | 3j3w_A: ribosome RNA 23S |
|  |  | 3j7a_7: tRNA |
|  |  | 3j7a_A: 18S ribosomal RNA |
|  |  | 4ifd_R: RNA (45-MER) |
|  |  | 5aj3_A: MITORIBOSOMAL 12S RRNA |
|  |  | 4wwt_1: 23S RRNA (2899-MER) |
|  |  | 4uer_A: 18S RRNA |
|  | | |
| 57752 | Ribosomal protein S14 | 1fjg_A: 16S RIBOSOMAL RNA |
|  |  | 1fjg_X: FRAGMENT OF MESSENGER RNA |
|  |  | 4a2i_A: 16S RIBOSOMAL RNA |
|  |  | 5aj3_A: MITORIBOSOMAL 12S RRNA |
|  | | |
| 144322 | Ribosomal protein L34p | 1xbp_0: 23S RIBOSOMAL RNA |
|  |  | 2j28_B: 23S RIBOSOMAL RNA |
|  |  | 2ftc_R: Mitochondrial 16S ribosomal RNA |
|  | | |
| 58331 | RSG-1.2 peptide | 1g70_A: HIV-1 RRE-IIB 32 NUCLEOTIDE RNA |
|  | | |
| 56054 | Ribosomal protein L6 | 1xbp_0: 23S RIBOSOMAL RNA |
|  |  | 1xbp_9: 5S RIBOSOMAL RNA |
|  |  | 1s72_0: 23S ribosomal RNA |
|  |  | 1s72_9: 5S ribosomal RNA |
|  |  | 3j7z_A: 23S rRNA |
|  |  | 3j16_J: 28S ribosomal RNA |
|  |  | 3j16_K: 18S ribosomal RNA |
|  |  | 3j16_L: P-site tRNA |
|  | | |
| 55268 | tRNA-intron endonuclease N-terminal domain-like | 2gjw_E: 5'-R(*GP*CP*GP*AP*CP*CP*GP*AP*CP*CP*AP*(DU) P*AP*GP*CP*UP*GP*CP*A)-3' |
|  |  | 2gjw_F: 5'-R(*UP*GP*CP*AP*GP*CP*GP*GP*UP*CP*AP*(A23))-3' |
|  |  | 2gjw_H: 5'-R(*AP*GP*GP*UP*CP*GP*C)-3' |
|  | | |
| 141341 | Gar1-like SnoRNP | 2hvy_E: H/ACA RNA |
|  | | |
| 58335 | TAT peptides | 1biv_A: BOVINE IMMUNODEFICIENCY VIRUS TAR RNA |
|  | | |
| 143812 | GatB/GatE catalytic domain-like | 2d6f_E: tRNA |
|  |  | 2d6f_F: tRNA |
|  | | |
| 103146 | Tombusvirus P19 core protein, VP19 | 1r9f_B: 5'- R(*CP*GP*UP*AP*CP*GP*CP*GP*GP*AP*AP*UP*AP*CP*UP*UP*CP*GP*AP *UP*U)-3' |
|  |  | 1r9f_C: 5'- R(*UP*CP*GP*AP*AP*GP*UP*AP*UP*UP*CP*CP*GP*CP*GP*UP*AP*CP*GP *UP*U)-3' |
|  |  | 4jk0_B: 5'-R(P*UP*UP*UP*GP*CP*UP*GP*CP*UP*GP*CP*UP*GP*CP*UP*GP*CP*UP*GP*CP*U)-3' |
|  |  | 4ktg_B: 5'-R(P*GP*CP*GP*GP*CP*GP*GP*CP*GP*GP*CP*GP*GP*CP*GP*GP*CP*GP*C)-3' |
|  |  | 4ktg_E: 5'-R(P*GP*CP*GP*GP*CP*GP*GP*CP*GP*GP*CP*GP*GP*CP*GP*GP*CP*GP*C)-3' |
|  |  | 1rpu_C: 5'- R(P*CP*GP*UP*AP*CP*GP*CP*GP*UP*CP*AP*CP*GP*CP*GP*UP*AP*CP*G P*UP*U)-3' |
|  |  | 1rpu_D: 5'- R(P*CP*GP*UP*AP*CP*GP*CP*GP*UP*CP*AP*CP*GP*CP*GP*UP*AP*CP*G P*UP*U)-3' |
|  | | |
| 52375 | Class I aminoacyl-tRNA synthetases (RS), catalytic domain | 1j1u_B: tRNA(Tyr) |
|  |  | 2ake_B: transfer RNA-Trp |
|  |  | 2azx_C: 72-MER |
|  |  | 1u0b_A: Cysteinyl-tRNA synthetase |
|  |  | 1g59_B: TRNA(GLU) |
|  |  | 1g59_D: TRNA(GLU) |
|  |  | 1n77_C: tRNA(Glu) |
|  |  | 1o0b_B: Glutaminyl tRNA |
|  |  | 2rd2_B: Glutamine tRNA |
|  |  | 1qrt_B: TRNAGLN2 |
|  |  | 1qru_B: TRNAGLN2 |
|  |  | 1qrs_B: TRNAGLN2 |
|  |  | 1gax_C: TRNA(VAL) |
|  |  | 1qu3_T: ISOLEUCYL-TRNA |
|  |  | 2rkj_C: RNA (238-MER) |
|  |  | 2rkj_G: RNA (238-MER) |
|  |  | 3al0_E: tRNAGln |
|  | | |
| 81311 | Fungal ribonucleases | 1b2m_C: 5'-R(*GP*(U34))-3' |
|  |  | 1b2m_D: 5'-R(*GP*(U34))-3' |
|  | | |
| 55406 | RNA bacteriophage capsid protein | 2qux_O: RNA (25-MER) |
|  |  | 2qux_R: RNA (25-MER) |
|  |  | 2b2d_R: 5'-R(*AP*UP*GP*CP*AP*UP*GP*UP*CP*UP*AP*AP*GP*AP*CP*AP*GP*CP*AP*U)-3' |
|  |  | 2bny_R: 5'-R(*AP*CP*AP*UP*GP*AP*GP*GP*AP*UP *UP*AP*CP*CP*CP*AP*UP*GP*U)-3' |
|  |  | 2bs1_R: 5'-R(*AP*CP*AP*UP*GP*AP*GP*GP*AP*UP *UP*AP*CP*CP*CP*AP*UP*GP*U)-3' |
|  |  | 1aq4_R: RNA (5'- R(*AP*CP*AP*UP*GP*AP*GP*GP*AP*UP*UP*AP*CP*CP*CP*AP*U P*GP*U)-3') |
|  |  | 1aq3_R: RNA (5'- R(*AP*CP*AP*UP*GP*AP*GP*GP*AP*UP*UP*AP*CP*CP*CP*AP*U P*GP*U)-3') |
|  |  | 1u1y_R: 5'-R(*CP*CP*GP*GP*(2PR) P*GP*GP*AP*UP*CP*AP*CP*CP*AP*CP*GP*G)-3' |
|  |  | 4l8h_R: RNA operator hairpin |
|  |  | 4ang_R: 5'-R(*CP*CP*AP*UP*AP*AP*GP*GP*AP*GP*CP*UP*AP*CP *CP*UP*AP*UP*GP*GP)-3' |
|  |  | 4ang_S: 5'-R(*CP*CP*AP*UP*AP*AP*GP*GP*AP*GP*CP*UP*AP*CP *CP*UP*AP*UP*GP*GP)-3' |
|  | | |
| 58124 | Large subunit | 1c04_E: 23S RRNA FRAGMENT |
|  |  | 1c04_F: 23S RRNA FRAGMENT |
|  |  | 1j5a_A: 23S RRNA |
|  |  | 1ml5_A: 30S 16S RIBOSOMAL RNA |
|  |  | 1ml5_a: 50S 23S RIBOSOMAL RNA |
|  |  | 1nkw_0: 23S ribosomal RNA |
|  |  | 1nwx_0: 23S RIBOSOMAL RNA |
|  |  | 1nwx_9: 5S RIBOSOMAL RNA |
|  |  | 1sm1_0: 23S RIBOSOMAL RNA |
|  |  | 1w2b_0: 23S RRNA |
|  |  | 1w2b_9: 5S RRNA |
|  |  | 2ftc_R: Mitochondrial 16S ribosomal RNA |
|  |  | 2qa4_0: 23S RIBOSOMAL RNA |
|  |  | 2qa4_9: 5S RIBOSOMAL RNA |
|  |  | 2ww9_D: 25S RRNA |
|  |  | 2ww9_E: 25S RRNA |
|  |  | 2ww9_F: 25S RRNA |
|  |  | 2ww9_G: 25S RRNA |
|  |  | 2wwb_D: 5.8S RRNA |
|  |  | 2wwb_E: 25S RRNA |
|  |  | 2wwb_F: 25S RRNA |
|  |  | 2wwb_G: 25S RRNA |
|  |  | 3cc2_0: 23S RIBOSOMAL RNA |
|  |  | 3cc2_9: 5S RIBOSOMAL RNA |
|  |  | 3ccr_0: 23S RIBOSOMAL RNA |
|  |  | 3ccr_9: 5S RIBOSOMAL RNA |
|  |  | 3cma_0: 23S RIBOSOMAL RNA |
|  |  | 3cma_5: RNA (5'-R(*CP*CP*A)-3') |
|  |  | 3cma_6: RNA (5'-R(*CP*CP*(8AN))-3') |
|  |  | 3cma_9: 5S RIBOSOMAL RNA |
|  |  | 3j0q_3: 60S ribosomal RNA fragment |
|  |  | 3j0q_9: 60S ribosomal RNA fragment |
|  |  | 3j0q_a: 40S ribosomal RNA fragment |
|  |  | 3j0q_w: mRNA fragment |
|  |  | 3j0q_W: tRNA |
|  |  | 3j0q_Y: tRNA |
|  |  | 3j16_J: 28S ribosomal RNA |
|  |  | 3j16_K: 18S ribosomal RNA |
|  |  | 3j16_L: P-site tRNA |
|  |  | 3j3v_A: ribosome RNA 23S |
|  |  | 3j3w_A: ribosome RNA 23S |
|  |  | 3j46_1: 23S ribosomal RNA |
|  |  | 3j46_2: 23S ribosomal RNA |
|  |  | 3j46_4: 23S ribosomal RNA |
|  |  | 3j5l_A: 23S ribsomal RNA |
|  |  | 3j7y_A: 16S rRNA |
|  |  | 3j7y_B: mt-tRNAVal |
|  |  | 3j7z_A: 23S rRNA |
|  |  | 3j7z_B: 5S rRNA |
|  |  | 3j8g_A: 5S rRNA |
|  |  | 3j8g_B: 23S rRNA |
|  |  | 3zc0_N: 5'-R(*UP*UP*CP*GP*AP*CP*GP*CP*GP*UP*CP*GP*AP* |
|  |  | 3zc0_O: 5'-R(*UP*UP*CP*GP*AP*CP*GP*CP*GP*UP*CP*GP*AP* |
|  |  | 4ce4_A: 16S RRNA |
|  |  | 4csu_A: 5S RRNA |
|  |  | 4csu_B: 23S RRNA |
|  |  | 4uy8_A: RRNA-23S RIBOSOMAL RNA |
|  |  | 4uy8_B: RRNA-5S RIBOSOMAL RNA |
|  |  | 4uy8_V: RNA |
|  |  | 4v19_A: MITORIBOSOMAL 16S RRNA |
|  |  | 4v19_C: TRNA |
|  |  | 4v19_Z: TRNA |
|  |  | 4wwe_1: 23S RRNA (2899-MER) |
|  |  | 4wwe_3: A SITE TNA, E SITE TNA |
|  |  | 4wwe_4: P SITE TRNA |
|  |  | 4wwe_s: 5S RRNA (119-MER) |
|  |  | 4wwt_1: 23S RRNA (2899-MER) |
|  |  | 4wwt_3: TRNA (76-MER) |
|  |  | 4wwt_s: THERMUS THERMOPHILUS HB8 GENOMIC DNA, COMPLETE GENOME |
|  |  | 4wwt_V: DNA (55-MER) |
|  |  | 5aka_B: 23S RNA |
|  | | |
| 90230 | CCCH zinc finger | 1rgo_D: RNA (5'-R(*UP*UP*AP*UP*UP*UP*AP*UP*U)-3') |
|  | | |
| 227287 | automated matches | 4x4s_B: G70A tRNA minihelix ending in CCACC |
|  |  | 4x4s_D: G70A tRNA minihelix ending in CCACC |
|  |  | 3ovb_C: RNA (35-MER) |
|  |  | 3ovb_D: RNA (35-MER) |
|  |  | 3ovs_C: RNA (34-MER) |
|  |  | 3ovs_D: RNA (34-MER) |
|  |  | 3ov7_C: RNA (34-MER) |
|  | | |
| 191529 | automated matches | 3p6y_W: 5'-R(*UP*GP*UP*AP*A)-3' |
|  |  | 2x1a_B: 5'-R(*GP*UP*UP*GP*UP)-3' |
|  |  | 2x1f_B: 5'-R(*GP*UP*UP*GP*UP)-3' |
|  |  | 4ed5_C: 5'-R(*A*UP*UP*UP*UP*UP*AP*UP*UP*UP*U)-3' |
|  |  | 2km8_A: 5'-R(P*UP*AP*UP*AP*UP*AP*UP*AP*AP*UP*AP*AP*U) |
|  |  | 2kg0_B: 5'-R(*AP*GP*GP*GP*AP*U)-3' |
|  |  | 2mb0_A: RNA_(5'-R(*AP*UP*CP*AP*AP*A)-3') |
|  |  | 2mgz_C: RNA (5'-R(*UP*GP*CP*AP*UP*GP*GP*UP*GP*UP*GP*C)-3') |
|  |  | 2rra_B: 5'-R(*GP*AP*AP*GP*AP*A)-3' |
|  |  | 2kfy_B: 5'-R(*AP*GP*GP*GP*AP*U)-3' |
|  |  | 2leb_B: RNA (5'-R(*UP*CP*CP*AP*GP*U)-3') |
|  |  | 2km8_A: 5'-R(P*UP*AP*UP*AP*UP*AP*UP*AP*AP*UP*AP*AP*U) |
|  |  | 4qu7_V: RNA 5'-(*AP*GP*GP*GP*AP*UP)-3' |
|  |  | 4qu6_B: RNA 5'-(*AP*GP*GP*GP*UP*GP)-3' |
|  |  | 3q2t_F: RNA |
|  |  | 3nnc_B: RNA (5'-R(*UP*GP*UP*GP*UP*GP*UP*UP*GP*UP*GP*UP*G)-3') |
|  |  | 4lmz_B: RNA (5'-R(*GP*CP*UP*GP*CP*GP*UP*AP*UP*UP*GP*UP*UP*UP*G)- 3') |
|  |  | 2rqc_B: 5'-R(*UP*GP*UP*GP*UP*G)-3' |
|  |  | 2yh1_B: 5'-R(*UP*UP*UP*UP*UP*UP*UP*UP*UP)-3' |
|  |  | 4bs2_B: 5'-R(*GP*UP*GP*UP*GP*AP*AP*UP*GP*AP*AP*UP)-3' |
|  |  | 4uer_A: 18S RRNA |
|  |  | 4n0t_B: U6 snRNA |
|  |  | 2xs2_B: 5'-R(*UP*UP*GP*UP*UP*CP*UP*U)-3' |
|  |  | 2kh9_B: 5'-R(*AP*GP*AP*GP*AP*U)-3' |
|  |  | 2rs2_B: RNA (5'-R(*GP*UP*AP*GP*U)-3') |
|  |  | 2i2y_B: (5'-R(*CP*AP*UP*C)-3') |
|  |  | 2g4b_B: 5'-R(P*UP*UP*UP*UP*UP*UP*U)-3' |
|  |  | 4cio_B: 5'-R(*GP*GP*UP*GP*UP*GP*CP)-3' |
|  |  | 2mki_B: RNA (5'-R(*CP*UP*UP*UP*A)-3') |
|  | | |
| 159112 | RNB domain-like | 2ix1_B: 5'-D(*AP*AP*AP*AP*AP*AP*AP*AP*AP*AP *AP*AP*A) |
|  |  | 4pmw_C: U-U-U-U-U-U-U-U-U-U-U-U-U-U |
|  |  | 4pmw_D: U-U-U-U-U-U-U-U-U-U-U-U-U-U |
|  |  | 4ifd_R: RNA (45-MER) |
|  | | |
| 227159 | automated matches | 2ann_B: 5'-R(*CP*GP*CP*GP*CP*GP*GP*AP*UP*CP*AP*GP*UP* |
|  |  | 3aev_C: RNA (5'-R(*GP*GP*AP*UP*CP*AP*CP*CP*UP*CP*C)-3 |
|  | | |
| 82661 | Poly A polymerase head domain-like | 4wc7_B: RNA (75-MER) |
|  |  | 3wfr_A: RNA (74-MER) |
|  |  | 3wfr_C: RNA (75-MER) |
|  |  | 3wfs_A: RNA (74-MER) |
|  |  | 3wfs_B: RNA (74-MER) |
|  | | |
| 54748 | Ribosomal L11/L12e N-terminal domain | 2rdo_B: 23S RIBOSOMAL RNA |
|  |  | 1xbp_0: 23S RIBOSOMAL RNA |
|  |  | 1mms_C: 23S RIBOSOMAL RNA |
|  |  | 1mms_D: 23S RIBOSOMAL RNA |
|  |  | 3j3v_A: ribosome RNA 23S |
|  |  | 3j3v_B: ribosome RNA 5S |
|  | | |
| 56048 | Ribosomal protein S8 | 1i95_A: 16S RRNA |
|  |  | 1s03_B: 47-MER |
|  |  | 1i6u_C: 16S RRNA FRAGMENT |
|  |  | 1i6u_D: 16S RRNA FRAGMENT |
|  |  | 1fjg_A: 16S RIBOSOMAL RNA |
|  |  | 4d61_1: 18S RRNA |
|  |  | 4d61_j: CRICKET PARALYSIS VIRUS IRES RNA |
|  |  | 3j80_2: 18S rRNA |
|  |  | 3j7a_7: tRNA |
|  |  | 3j7a_A: 18S ribosomal RNA |
|  |  | 4uer_A: 18S RRNA |
|  | | |
| 102581 | (Uracil-5-)-methyltransferase | 2bh2_D: 23S RIBOSOMAL RNA 1932-1968 |
|  |  | 3bt7_D: RNA (5'-D(P*GP*CP*UP*GP*UP*GP*(5MU) P*UP*CP*GP*AP*UP*CP*CP*AP*CP*AP*GP*C)-3') |
|  | | |
| 88641 | Tymoviridae-like VP | 1ddl_D: RNA (5'-R(P*UP*UP*UP*UP*UP*UP*U)-3') |
|  |  | 1ddl_E: RNA (5'-R(P*UP*U)-3') |
|  |  | 2fz2_D: 5'-R(*CP*CP*C)-3' |
|  |  | 2xpj_D: 5'-R(CP*CP*CP)-3' |
|  | | |
| 58345 | HTLV-1 peptide | 1exy_A: RNA APTAMER, 33-MER |
|  | | |
| 52652 | Nitrogenase iron protein-like | 2iy3_B: 4.5S RNA |
|  |  | 1qzw_B: 7S RNA |
|  |  | 1qzw_H: 7S RNA |
|  |  | 2j28_8: 4.5S SIGNAL RECOGNITION PARTICLE RNA |
|  |  | 2j28_B: 23S RIBOSOMAL RNA |
|  |  | 4c7o_E: SRP RNA |
|  |  | 2xxa_F: 4.5S RNA |
|  |  | 2xxa_G: 4.5S RNA |
|  |  | 2j37_A: SRP RNA |
|  |  | 2j37_Z: RIBOSOMAL RNA |
|  |  | 2xkv_B: 4.5S RNA |
|  | | |
| 88639 | Bromoviridae-like VP | 1cwp_D: RNA (5'-R(*AP*UP*AP*U)-3') |
|  |  | 1laj_R: 5'-R(*AP*AP*A)-3' |
|  | | |
| 50194 | Ribosomal protein L14 | 1ffk_0: 23S RRNA |
|  |  | 1ffk_9: 5S RRNA |
|  |  | 1xbp_0: 23S RIBOSOMAL RNA |
|  |  | 1xbp_9: 5S RIBOSOMAL RNA |
|  |  | 3bbx_B: 23S ribosomal RNA |
|  |  | 1s72_0: 23S ribosomal RNA |
|  |  | 1s72_9: 5S ribosomal RNA |
|  | | |
| 52592 | G proteins | 1zc8_A: TLD 16S ribosomal RNA |
|  |  | 3a6p_D: pre-microRNA |
|  |  | 3a6p_E: pre-microRNA |
|  |  | 1ob2_B: TRANSFER-RNA, PHE |
|  |  | 3v11_D: Initiator tRNA |
|  |  | 3iev_D: 5'-R(P*AP*UP*CP*AP*CP*CP*UP*CP*CP*UP*UP*A)-3' |
|  |  | 4cxg_a: 18S RRNA - H5-H14 |
|  |  | 4cxg_c: 28S RRNA - H95 |
|  |  | 4cxg_x: MESSENGER RNA |
|  |  | 4cxg_Y: TRANSFER RNA |
|  |  | 3icq_D: RNA (62-MER) |
|  |  | 3j81_1: Met-tRNAi |
|  |  | 3j8g_B: 23S rRNA |
|  |  | 3deg_A: A/L-tRNA |
|  |  | 3deg_B: P-tRNA |
|  |  | 3deg_G: 50S RNA helix 42-44 |
|  |  | 3deg_I: 50S RNA helix 95 |
|  |  | 3deg_J: 50S RNA helix 71 |
|  |  | 3avy_G: RNA (5'-R(*GP*GP*GP*UP*CP*CP*AP*UP*AP*AP*AP*AP*U)-3') |
|  |  | 3avy_T: RNA (5'- R(*AP*AP*CP*GP*AP*UP*UP*UP*UP*AP*UP*GP*GP*AP*CP*CP*CP*A)-3') |
|  |  | 4d61_1: 18S RRNA |
|  |  | 4d61_j: CRICKET PARALYSIS VIRUS IRES RNA |
|  | | |
| 50716 | Ribosomal protein L25-like | 2rdo_A: 5S RIBOSOMAL RNA |
|  |  | 1xbp_0: 23S RIBOSOMAL RNA |
|  |  | 1xbp_9: 5S RIBOSOMAL RNA |
|  |  | 1feu_E: 19 NT FRAGMENT OF 5S RRNA |
|  |  | 1feu_F: 21 NT FRAGMENT OF 5S RRNA |
|  |  | 4wwe_1: 23S RRNA (2899-MER) |
|  |  | 4wwe_s: 5S RRNA (119-MER) |
|  | | |
| 50183 | Sm motif of small nuclear ribonucleoproteins, SNRNP | 1i5l_U: 5'-R(*UP*UP*U)-3' |
|  |  | 1m8v_P: 5'-R(P*UP*UP*UP*UP*UP*UP*U)-3' |
|  |  | 1m8v_T: 5'-R(P*UP*UP*UP*UP*UP*UP*U)-3' |
|  |  | 4m7a_P: U6 snRNA |
|  |  | 4pjo_1: U1 RNA variant (48-MER) with 4-helix junction |
|  |  | 4pjo_2: U1 RNA variant (48-MER) with 4-helix junction |
|  |  | 4m7d_P: U6 snRNA |
|  |  | 4m7d_O: U6 snRNA |
|  |  | 4m7d_P: U6 snRNA |
|  |  | 4m7a_O: U6 snRNA |
|  |  | 4m7a_P: U6 snRNA |
|  |  | 4m7a_P: U6 snRNA |
|  |  | 4pjo_1: U1 RNA variant (48-MER) with 4-helix junction |
|  |  | 4pjo_2: U1 RNA variant (48-MER) with 4-helix junction |
|  |  | 4pjo_X: RNA (5'-R(*AP*GP*GP*UP*AP*AP*GP*UP*CP*C)-3') |
|  |  | 4m7a_P: U6 snRNA |
|  |  | 4m7a_P: U6 snRNA |
|  |  | 4pjo_1: U1 RNA variant (48-MER) with 4-helix junction |
|  |  | 4pjo_2: U1 RNA variant (48-MER) with 4-helix junction |
|  |  | 4pjo_3: U1 RNA variant (48-MER) with 4-helix junction |
|  |  | 4pjo_4: U1 RNA variant (48-MER) with 4-helix junction |
|  |  | 4pjo_y: RNA (5'-R(*AP*GP*GP*UP*AP*AP*GP*UP*CP*C)-3') |
|  |  | 4pjo_1: U1 RNA variant (48-MER) with 4-helix junction |
|  |  | 4pjo_2: U1 RNA variant (48-MER) with 4-helix junction |
|  |  | 4pjo_X: RNA (5'-R(*AP*GP*GP*UP*AP*AP*GP*UP*CP*C)-3') |
|  |  | 4pjo_3: U1 RNA variant (48-MER) with 4-helix junction |
|  |  | 4pjo_4: U1 RNA variant (48-MER) with 4-helix junction |
|  |  | 4pjo_Y: RNA (5'-R(*AP*GP*GP*UP*AP*AP*GP*UP*CP*C)-3') |
|  |  | 3cw1_V: U1 snRNA |
|  |  | 3cw1_V: U1 snRNA |
|  | | |
| 82075 | Kid/PemK | 2c06_C: 5'-R(*AP*UP*AP*CP*AP)-3' |
|  |  | 4mdx_C: RNA, mRNA |
|  | | |
| 53733 | Aconitase iron-sulfur domain | 3snp_C: ferritin H IRE RNA |
|  |  | 3snp_D: ferritin H IRE RNA |
|  | | |
| 254188 | RIG-I C-terminal domain-like | 3og8_C: RNA (5'-R(*GP*GP*CP*GP*CP*GP*CP*GP*CP*GP*CP*GP*CP*C)-3') |
|  |  | 3og8_D: RNA (5'-R(*GP*GP*CP*GP*CP*GP*CP*GP*CP*GP*CP*GP*CP*C)-3') |
|  |  | 2ykg_C: 5'-R(*GP*CP*GP*CP*GP*CP*GP*CP*GP*CP)-3' |
|  |  | 2ykg_D: 5'-R(*GP*CP*GP*CP*GP*CP*GP*CP*GP*CP)-3' |
|  |  | 4a2x_L: 5'-R(*GP*GP*GP*AP*GP*AP*AP*CP*AP*AP*CP*GP*CP*GP)-3' |
|  |  | 4a2x_M: 5'-R(*CP*GP*CP*GP*UP*UP*GP*UP*UP*CP*UP*CP*CP*CP)-3' |
|  |  | 3eqt_C: 5'-R(*GP*CP*GP*CP*GP*CP*GP*C)-3' |
|  |  | 3eqt_D: 5'-R(*GP*CP*GP*CP*GP*CP*GP*C)-3' |
|  |  | 4gl2_C: RNA (5'-R(*AP*UP*CP*CP*GP*CP*GP*GP*CP*CP*CP*U)-3') |
|  |  | 4gl2_D: RNA (5'-R(P*AP*GP*GP*GP*CP*CP*GP*CP*GP*GP*AP*U)-3') |
|  |  | 4gl2_E: RNA (5'-R(*AP*UP*CP*CP*GP*CP*GP*GP*CP*CP*CP*U)-3') |
|  |  | 4gl2_F: RNA (5'-R(P*AP*GP*GP*GP*CP*CP*GP*CP*GP*GP*AP*U)-3') |
|  |  | 3tmi_B: 5'-R(*CP*GP*AP*CP*GP*CP*UP*AP*GP*CP*GP*UP*CP*G)-3' |
|  |  | 3tmi_C: 5'-R(*CP*GP*AP*CP*GP*CP*UP*AP*GP*CP*GP*UP*CP*G)-3' |
|  |  | 4ay2_C: 5'-R-PPP(GP*GP*CP*GP*CP*GP*GP*CP*UP*UP*CP*GP*GP*CP *CP*GP*CP*GP*CP*C)-3' |
|  |  | 3zd7_C: RNA DUPLEX |
|  |  | 3zd7_D: RNA DUPLEX |
|  |  | 4bpb_C: 5'-R(*GP*CP*GP*CP*GP*CP*GP*CP*GP*CP)-3' |
|  |  | 4bpb_D: 5'-R(*GP*CP*GP*CP*GP*CP*GP*CP*GP*CP)-3' |
|  |  | 3zd6_C: RNA DUPLEX |
|  |  | 3zd6_D: RNA DUPLEX |
|  | | |
| 52670 | RecA protein-like (ATPase-domain) | 3ice_G: 5'-R(P*UP*UP*UP*UP*UP*UP*UP*UP*UP*UP*UP*U)-3' |
|  |  | 2ht1_J: 5'-R(*UP*C)-3' |
|  |  | 2ht1_M: 5'-R(*UP*CP*UP*CP*U)-3' |
|  | | |
| 74683 | C-terminal fragment of elongation factor SelB | 1wsu_G: 5'- R(*GP*GP*CP*GP*UP*UP*GP*CP*CP*GP*GP*UP*CP*U*GP*GP*CP*AP*AP* CP*GP*CP*C)-3' |
|  |  | 2uwm_C: 5'-R(*GP*GP*CP*GP*UP*UP*GP*CP*CP*GP *GP*UP*CP*UP*GP*GP*CP*AP*AP*CP*GP*CP*C)-3' |
|  |  | 2uwm_D: 5'-R(*GP*GP*CP*GP*UP*UP*GP*CP*CP*GP *GP*UP*CP*UP*GP*GP*CP*AP*AP*CP*GP*CP*C)-3' |
|  |  | 2ply_C: RNA (5'- R(*GP*GP*CP*GP*UP*UP*GP*CP*CP*GP*GP*UP*CP*UP*GP*GP*CP*AP*AP *CP*GP*CP*C)-3') |
|  |  | 2ply_E: RNA (5'- R(*GP*GP*CP*GP*UP*UP*GP*CP*CP*GP*GP*UP*CP*UP*GP*GP*CP*AP*AP *CP*GP*CP*C)-3') |
|  |  | 2pjp_B: SECIS RNA |
|  | | |
| 55201 | Translation initiation factor IF3, C-terminal domain | 1i96_A: 16S RRNA |
|  | | |
| 142565 | RoRNP C-terminal domain-like | 2i91_C: 5'-R(*GP*CP*CP*UP*AP*CP*CP*C)-3' |
|  |  | 2i91_D: 5'- R(*C*GP*GP*UP*AP*GP*GP*CP*UP*UP*UP*UP*CP*AP*A)-3' |
|  |  | 2i91_F: 5'- R(*C*GP*GP*UP*AP*GP*GP*CP*UP*UP*UP*UP*CP*AP*A)-3' |
|  | | |
| 52314 | Ribosomal protein S2 | 2vqe_A: 16S RRNA |
|  |  | 5aj3_A: MITORIBOSOMAL 12S RRNA |
|  |  | 4d5l_1: 18S RRNA 2 |
|  |  | 4adv_A: 16S RIBOSOMAL RNA |
|  |  | 4uer_A: 18S RRNA |
|  |  | 3j80_2: 18S rRNA |
|  |  | 4kzz_i: 18S Ribosomal RNA |
|  |  | 3j7a_A: 18S ribosomal RNA |
|  | | |
| 69904 | NSP3 homodimer | 1knz_W: 5'-R(*UP*GP*AP*CP*C)-3' |
|  | | |
| 55178 | Ribosomal protein S4 (bacteria) | 1fjg_A: 16S RIBOSOMAL RNA |
|  |  | 1hnw_A: 16S RIBOSOMAL RNA |
|  |  | 4a2i_A: 16S RIBOSOMAL RNA |
|  | | |
| 57841 | Ribosomal protein L36 | 1xbp_0: 23S RIBOSOMAL RNA |
|  |  | 1xbp_9: 5S RIBOSOMAL RNA |
|  |  | 2j28_B: 23S RIBOSOMAL RNA |
|  | | |
| 57833 | Ribosomal protein L37e | 2otl_0: 23S ribosomal RNA |
|  |  | 2otl_9: 5S ribosomal RNA |
|  | | |
| 54566 | Ribosomal protein S16 | 1i95_A: 16S RRNA |
|  | | |
| 50105 | Ribosomal proteins L24p and L21e | 2rdo_B: 23S RIBOSOMAL RNA |
|  |  | 1xbp_0: 23S RIBOSOMAL RNA |
|  |  | 1s72_0: 23S ribosomal RNA |
|  |  | 1s72_9: 5S ribosomal RNA |
|  |  | 1s72_0: 23S ribosomal RNA |
|  |  | 1s72_9: 5S ribosomal RNA |
|  |  | 2ww9_D: 25S RRNA |
|  |  | 2ww9_E: 25S RRNA |
|  |  | 2ww9_F: 25S RRNA |
|  |  | 2ww9_G: 25S RRNA |
|  | | |
| 57830 | Ribosomal protein L37ae | 1ffk_0: 23S RRNA |
|  |  | 1jj2_0: 23S RRNA |
|  |  | 3cma_0: 23S RIBOSOMAL RNA |
|  |  | 2qa4_0: 23S RIBOSOMAL RNA |
|  |  | 1ysh_A: RNA (28-MER) |
|  |  | 1ysh_B: RNA (101-MER) |
|  |  | 1ysh_F: RNA (34-MER) |
|  | | |
| 90019 | SRP alpha N-terminal domain-like | 2go5_A: SRP RNA |
|  | | |
| 58120 | Ribosome complexes | 1ibk_A: 16S RIBOSOMAL RNA |
|  |  | 1jgo_1: MESSENGER RNA MK27 |
|  |  | 1jgo_A: 30S 16S ribosomal RNA |
|  |  | 1jgo_C: tRNA(Phe) |
|  |  | 1jgo_D: tRNA(Phe) |
|  |  | 1ls2_B: Phenylalanine transfer RNA |
|  |  | 1mj1_D: Phe-tRNA |
|  |  | 1mj1_Q: sarcin-ricin loop of 23SrRNA |
|  |  | 1ml5_A: 30S 16S RIBOSOMAL RNA |
|  |  | 1ml5_a: 50S 23S RIBOSOMAL RNA |
|  |  | 1ml5_B: T-RNA(PHE) |
|  |  | 1ml5_C: A- AND P-SITE MESSENGER RNA CODONS |
|  |  | 1r2x_C: 58nts of 23S rRNA |
|  |  | 2wwa_D: 25S RRNA |
|  |  | 2ykr_A: 16S RRNA |
|  |  | 3bbx_A: 5S ribosomal RNA |
|  |  | 3bbx_B: 23S ribosomal RNA |
|  |  | 3cc2_0: 23S RIBOSOMAL RNA |
|  |  | 3j0l_6: 60S ribosomal RNA fragment |
|  |  | 3j0l_a: 40S ribosomal RNA fragment |
|  |  | 3j0l_b: 40S ribosomal RNA fragment |
|  |  | 3j0l_c: 40S ribosomal RNA fragment |
|  |  | 3j0l_e: 40S ribosomal RNA fragment |
|  |  | 3j0l_E: 40S ribosomal RNA fragment |
|  |  | 3j0l_g: 40S ribosomal RNA fragment |
|  |  | 3j0l_G: 40S ribosomal RNA fragment |
|  |  | 3j0l_h: 40S ribosomal RNA fragment |
|  |  | 3j0l_V: tRNA |
|  |  | 3j0l_W: tRNA |
|  |  | 3j0l_y: mRNA fragment |
|  |  | 3j0l_Y: tRNA |
|  |  | 3j7a_7: tRNA |
|  |  | 3j7a_A: 18S ribosomal RNA |
|  |  | 3j7y_A: 16S rRNA |
|  |  | 3j80_2: 18S rRNA |
|  |  | 3j81_2: 18S rRNA |
|  |  | 3j81_3: mRNA |
|  |  | 3ow2_0: 23S RIBOSOMAL RNA |
|  |  | 3ow2_9: 5S RIBOSOMAL RNA |
|  |  | 3pio_X: RIBOSOMAL 23S RNA |
|  |  | 3t1y_A: 16S rRNA |
|  |  | 4adv_A: 16S RIBOSOMAL RNA |
|  |  | 4d5l_1: 18S RRNA 2 |
|  |  | 4d61_1: 18S RRNA |
|  |  | 4d61_j: CRICKET PARALYSIS VIRUS IRES RNA |
|  |  | 4dr1_A: 16S rRNA |
|  |  | 4dv4_A: 16S rRNA |
|  |  | 4fsj_R: Random cellular RNA fragments |
|  |  | 4ioa_X: 23S ribosomal RNA |
|  |  | 4ioa_Y: 5S ribosomal RNA |
|  |  | 4ji1_A: 16S rRNA |
|  |  | 4ji3_A: 16S rRNA |
|  |  | 4kvb_A: 16S rRNA |
|  |  | 4kzx_i: 18S ribosomal RNA |
|  |  | 4kzz_i: 18S Ribosomal RNA |
|  |  | 4kzz_j: initiator Met-RNA-i |
|  |  | 4lf4_A: 16S rRNA |
|  |  | 4lf9_A: 16S rRNA |
|  |  | 4ox9_A: 16S rRNA |
|  |  | 4uer_A: 18S RRNA |
|  |  | 4uy8_A: RRNA-23S RIBOSOMAL RNA |
|  |  | 4uy8_V: RNA |
|  |  | 4wwe_1: 23S RRNA (2899-MER) |
|  |  | 5aj3_A: MITORIBOSOMAL 12S RRNA |
|  | | |
| 51714 | tRNA-guanine transglycosylase | 1q2r_E: RNA (5'-R(*AP*GP*CP*AP*CP*GP*GP*CP*UP*(N) P*UP*AP*AP*AP*CP*CP*GP*UP*GP*C)-3') |
|  | | |
| 55316 | L30e/L7ae ribosomal proteins | 1ffk_0: 23S RRNA |
|  |  | 1t0k_C: 5'-R(*GP*AP*CP*CP*GP*GP*AP*GP*UP*GP*UP*CP*C)-3' |
|  |  | 1t0k_D: 5'- R(*G*GP*AP*CP*GP*CP*AP*GP*AP*GP*AP*UP*GP*GP*UP*C)-3' |
|  |  | 1sds_D: box H/ACA sRNA |
|  |  | 1sds_E: box H/ACA sRNA |
|  |  | 1sds_F: box H/ACA sRNA |
|  |  | 1rlg_C: 25-MER |
|  |  | 3cma_0: 23S RIBOSOMAL RNA |
|  |  | 3cma_9: 5S RIBOSOMAL RNA |
|  |  | 2hvy_E: H/ACA RNA |
|  |  | 2ozb_F: RNA comprising the 5' Stem-Loop RNA of U4snR |
|  |  | 4bw0_A: HMKT-7 |
|  |  | 3v7e_C: SAM-I riboswitch aptamer with an engineered helix P3 |
|  |  | 3v7e_D: SAM-I riboswitch aptamer with an engineered helix P3 |
|  |  | 3pla_G: C/D guide RNA |
|  |  | 3pla_H: C/D guide RNA |
|  |  | 3pla_J: RNA (5'-R(*CP*CP*AP*UP*GP*AP*GP*UP*GP*U)-3') |
|  |  | 4d61_1: 18S RRNA |
|  |  | 4uer_A: 18S RRNA |
|  |  | 3nmu_E: RNA (34-MER) |
|  |  | 3nmu_I: RNA (5'-R(*GP*AP*GP*CP*UP*UP*CP*AP*AP*CP*GP*GP*C)-3') |
|  |  | 3nmu_K: RNA (5'-R(*GP*AP*GP*CP*UP*UP*CP*AP*AP*CP*GP*GP*C)-3') |
|  |  | 3lwq_D: H/ACA RNA |
|  |  | 3j80_2: 18S rRNA |
|  |  | 3j7a_A: 18S ribosomal RNA |
|  |  | 4adx_0: 23S RRNA |
|  |  | 1ysh_B: RNA (101-MER) |
|  |  | 1ysh_F: RNA (34-MER) |
|  | | |
| 82705 | DNA-binding protein AlbA | 3wbm_X: RNA (25-MER) |
|  |  | 3wbm_Y: RNA (25-MER) |
|  |  | 3iab_R: P3 domain of the RNA component of RNase MRP |
|  |  | 3iab_R: P3 domain of the RNA component of RNase MRP |
|  | | |
| 89800 | Deoxycytidylate deaminase-like | 2b3j_E: ANTICODON STEM-LOOP OF T-RNA-ARG2 (NUCLEOTIDES 27-42) |
|  |  | 2b3j_F: ANTICODON STEM-LOOP OF T-RNA-ARG2 (NUCLEOTIDES 27-42) |
|  |  | 2b3j_H: ANTICODON STEM-LOOP OF T-RNA-ARG2 (NUCLEOTIDES 27-42) |
|  | | |
| 74983 | Small protein B (SmpB) | 2ob7_A: transfer-messenger RNA |
|  |  | 2czj_B: tmRNA (63-MER) |
|  |  | 2czj_D: tmRNA (63-MER) |
|  |  | 3iyq_A: tmRNA |
|  | | |
| 48942 | C1 set domains (antibody constant domain-like) | 3agv_S: 5'-R(*GP*GP*AP*GP*GP*(UFT)P*GP*(CFZ)P*(UFT)P*(CFZ)P*(CFZ) P*GP*AP*AP*A*GP*GP*AP*AP*(CFZ)P*(UFT)P*(CFZ)P*(CFZ)P*A)-3' |
|  | | |
| 55121 | Pseudouridine synthase I TruA | 2nre_F: leucyl tRNA |
|  | | |
| 227218 | automated matches | 2po1_C: 10-mer poly(A) |
|  |  | 2po1_C: 10-mer poly(A) |
|  |  | 4ifd_R: RNA (45-MER) |
|  |  | 4ifd_R: RNA (45-MER) |
|  |  | 4ifd_R: RNA (45-MER) |
|  | | |
| 56372 | Plant cytotoxins | 3rtj_D: RNA (5'-R(*AP*G)-3') |
|  | | |
| 56554 | Insert subdomain of RNA polymerase alpha subunit | 2b63_R: 31-MER |
|  | | |
| 81589 | Poly(A) polymerase, PAP, N-terminal domain | 2q66_X: 5'-R(P*AP*AP*AP*AP*A)-3' |
|  |  | 4nku_D: 5'-R(*AP*U)-3' |
|  |  | 4nku_H: 5'-R(*AP*U)-3' |
|  | | |
| 53329 | Formyltransferase | 2fmt_C: FORMYL-METHIONYL-TRNAFMET2 |
|  |  | 2fmt_D: FORMYL-METHIONYL-TRNAFMET2 |
|  | | |
| 55682 | Class II aminoacyl-tRNA synthetase (aaRS)-like, catalytic domain | 2iy5_T: TRNAPHE |
|  |  | 1ser_T: TRNASER |
|  |  | 1h4q_T: TRNAPRO(CGG) |
|  |  | 1asy_R: T-RNA (75-MER) |
|  |  | 1asy_S: T-RNA (75-MER) |
|  |  | 1il2_C: ASPARTYL TRANSFER RNA |
|  |  | 1il2_D: ASPARTYL TRANSFER RNA |
|  |  | 1efw_C: ASPARTYL-TRNA |
|  |  | 1efw_D: ASPARTYL-TRNA |
|  |  | 1qf6_B: THREONINE TRNA |
|  |  | 2iy5_T: TRNAPHE |
|  |  | 1eiy_C: TRNA(PHE) |
|  |  | 3kfu_N: tRNA-Asn |
|  |  | 4wj3_Q: 76mer-tRNA |
|  |  | 4wj3_R: 76mer-tRNA |
|  |  | 4wj3_S: 76mer-tRNA |
|  |  | 4wj3_T: 76mer-tRNA |
|  |  | 4kr2_C: Gly-tRNA-CCC |
|  |  | 4kr3_C: Gly-tRNA-CCC |
|  |  | 3j7y_A: 16S rRNA |
|  |  | 3w3s_B: selenocysteine tRNA |
|  |  | 4ce4_A: 16S RRNA |
|  | | |
| 191340 | automated matches | 3bx3_C: COX17 RNA target sequence |
|  |  | 3bx3_D: COX17 RNA target sequence |
|  |  | 3k49_B: RNA (5'-R(*CP*CP*UP*GP*UP*AP*AP*AP*UP*A)-3') |
|  |  | 3k49_F: RNA (5'-R(*CP*CP*UP*GP*UP*AP*AP*AP*UP*A)-3') |
|  |  | 3v74_B: RNA (5'-R(*UP*CP*AP*UP*GP*UP*GP*CP*CP*AP*UP*AP*C)-3') |
|  |  | 3v71_B: RNA (5'-R(*CP*UP*CP*UP*GP*UP*AP*UP*CP*UP*UP*GP*U)-3') |
|  |  | 4dzs_C: 5'-R(*UP*GP*UP*AP*UP*CP*AP*UP*A)-3' |
|  |  | 3qgb_B: 5'-R(*UP*GP*UP*GP*CP*CP*AP*UP*A)-3' |
|  |  | 3icq_D: RNA (62-MER) |
|  | | |
| 88785 | mRNA cap methylase | 2xbm_E: 5'-(*G3AP*GP*AP*AP*CP*CP*UP*GP*A)-3' |
|  |  | 2xbm_F: 5'-(*G3AP*GP*AP*AP*CP*CP*UP*GP*A)-3' |
|  |  | 3er8_G: RNA/DNA chimera 5'-D(P*CP*)R(UP*U)-3' |
|  |  | 1av6_B: RNA (5'-R(*GP*AP*AP*AP*AP*A)-3') |
|  |  | 4n48_D: capped RNA |
|  |  | 3a6p_E: pre-microRNA |
|  | | |
| 54778 | Ribosomal S5 protein, N-terminal domain | 1fjg_A: 16S RIBOSOMAL RNA |
|  |  | 4adv_A: 16S RIBOSOMAL RNA |
|  |  | 5aj3_A: MITORIBOSOMAL 12S RRNA |
|  | | |
| Cluster26 | NA | 4u7u_L: crRNA |
|  | | |
| Cluster110 | NA | 4puq_C: DNA/RNA hybrid |
|  |  | 4puq_D: DNA/RNA hybrid |
|  | | |
| Cluster35 | NA | 3iy9_A: Leishmania Tarentolae Mitochondrial Large Ribosomal subunit |
|  | | |
| Cluster5 | NA | 4kzy_i: 18S Ribosomal RNA |
|  |  | 3j80_2: 18S rRNA |
|  |  | 3j7a_7: tRNA |
|  |  | 3j7a_A: 18S ribosomal RNA |
|  |  | 4uer_A: 18S RRNA |
|  | | |
| Cluster78 | NA | 4wj3_T: 76mer-tRNA |
|  |  | 3kfu_K: tRNA-Asn |
|  |  | 3kfu_M: tRNA-Asn |
|  |  | 3kfu_N: tRNA-Asn |
|  | | |
| Cluster48 | NA | 4wj3_R: 76mer-tRNA |
|  |  | 4wj3_T: 76mer-tRNA |
|  |  | 3kfu_L: tRNA-Asn |
|  |  | 3kfu_M: tRNA-Asn |
|  |  | 3kfu_N: tRNA-Asn |
|  |  | 3al0_E: tRNAGln |
|  | | |
| Cluster20 | NA | 4nxm_A: 16S rRNA |
|  | | |
| Cluster82 | NA | 3cf5_X: RRNA-23S RIBOSOMAL RNA |
|  | | |
| Cluster88 | NA | 4oo1_S: POLY A RNA |
|  |  | 4ifd_R: RNA (45-MER) |
|  | | |
| Cluster62 | NA | 4o26_E: Telomerase TR |
|  |  | 4o26_F: Telomerase TR |
|  | | |
| Cluster103 | NA | 3a2k_C: bacterial tRNA |
|  |  | 3a2k_D: bacterial tRNA |
|  | | |
| Cluster89 | NA | 4wwt_1: 23S RRNA (2899-MER) |
|  |  | 4wwt_3: TRNA (76-MER) |
|  |  | 4wwt_4: TRNA (76-MER) |
|  |  | 4wwt_V: DNA (55-MER) |
|  | | |
| Cluster49 | NA | 4d5l_1: 18S RRNA 2 |
|  |  | 3j0p_a: 40S ribosomal RNA fragment |
|  |  | 3j0p_d: 40S ribosomal RNA fragment |
|  |  | 3j0p_f: 40S ribosomal RNA fragment |
|  |  | 3j0p_g: 40S ribosomal RNA fragment |
|  |  | 3j0p_G: 40S ribosomal RNA fragment |
|  |  | 3j0p_w: mRNA fragment |
|  |  | 3j0p_W: tRNA |
|  |  | 4kzz_i: 18S Ribosomal RNA |
|  |  | 3j16_J: 28S ribosomal RNA |
|  |  | 3j7a_7: tRNA |
|  |  | 3j7a_A: 18S ribosomal RNA |
|  |  | 3j81_1: Met-tRNAi |
|  |  | 3j81_2: 18S rRNA |
|  |  | 3j81_3: mRNA |
|  | | |
| Cluster67 | NA | 3d2s_F: 5'-R(*CP*GP*CP*UP*GP*U)-3' |
|  | | |
| Cluster1 | NA | 4oav_A: RNA (5'-R(P*(PO4)P*(PO4)P*AP*AP*AP*AP*(PO4))-2') |
|  |  | 4oav_C: RNA (5'-R(P*(PO4)P*(PO4)P*AP*AP*AP*AP*(PO4))-2') |
|  | | |
| Cluster2 | NA | 3zla_J: RNA |
|  |  | 4j1g_E: RNA (45-MER) |
|  |  | 4jng_L: RNA (42-MER) |
|  |  | 4ijs_E: RNA (5'-R(P*AP*AP*AP*AP*AP*AP*AP*AP*AP*A)-3') |
|  |  | 4ijs_F: RNA (5'-R(P*AP*AP*AP*AP*AP*AP*AP*AP*AP*A)-3') |
|  |  | 4ijs_G: RNA (5'-R(P*AP*AP*AP*AP*AP*AP*AP*AP*AP*A)-3') |
|  |  | 4ijs_H: RNA (5'-R(P*AP*AP*AP*AP*AP*AP*AP*AP*AP*A)-3') |
|  |  | 4bhh_R: POLY-URIDINE 45-MER |
|  | | |
| Cluster125 | NA | 3vjr_B: tRNA CCA-acceptor |
|  |  | 3vjr_D: tRNA CCA-acceptor |
|  | | |
| Cluster85 | NA | 3mdi_C: RNA (5'-R(*UP*GP*UP*AP*AP*A)-3') |
|  |  | 3p6y_R: 5'-R(*UP*GP*UP*AP*A)-3' |
|  |  | 3p6y_S: 5'-R(*UP*GP*UP*AP*A)-3' |
|  | | |
| Cluster98 | NA | 3rw6_F: constitutive transport element(CTE)of Mason-P |
|  |  | 3rw6_H: constitutive transport element(CTE)of Mason-P |
|  | | |
| Cluster127 | NA | 3bo1_G: 23S RIBOSOMAL RNA |
|  | | |
| Cluster128 | NA | 3j3w_A: ribosome RNA 23S |
|  | | |
| Cluster94 | NA | 2xfm_B: 5'-R(*AP*CP*CP*GP*AP*CP*UP*(OMU)P)-3' |
|  |  | 2l5d_B: 5'-R(*UP*GP*AP*CP*A)-3' |
|  | | |
| Cluster92 | NA | 4ce4_A: 16S RRNA |
|  | | |
| Cluster33 | NA | 4kzz_i: 18S Ribosomal RNA |
|  |  | 3j80_2: 18S rRNA |
|  |  | 4uer_A: 18S RRNA |
|  | | |
| Cluster55 | NA | 4ngc_B: 5'-R(*GP*CP*GP*AP*AP*UP*UP*CP*GP*CP*UP*A)-3' |
|  |  | 4nha_B: 5'-R(P*GP*CP*GP*UP*UP*GP*GP*CP*CP*AP*AP*CP*GP*CP*UP*U)-3' |
|  | | |
| Cluster63 | NA | 4m59_C: psaJ RNA |
|  |  | 4m59_D: psaJ RNA |
|  |  | 4oe1_C: psaJ RNA |
|  |  | 4oe1_D: psaJ RNA |
|  | | |
| Cluster117 | NA | 4adv_A: 16S RIBOSOMAL RNA |
|  | | |
| Cluster45 | NA | 4kzy_i: 18S Ribosomal RNA |
|  |  | 3j80_2: 18S rRNA |
|  |  | 4uer_A: 18S RRNA |
|  |  | 3j7a_7: tRNA |
|  |  | 3j7a_A: 18S ribosomal RNA |
|  |  | 4d5l_1: 18S RRNA 2 |
|  | | |
| Cluster53 | NA | 3am1_B: ASL-truncated tRNA |
|  |  | 3adc_C: selenocysteine tRNA |
|  |  | 4qm6_C: RNA |
|  |  | 4qm6_D: RNA |
|  | | |
| Cluster74 | NA | 4kji_D: RsmZ-2 |
|  | | |
| Cluster42 | NA | 2j0q_H: 5'-R(*UP*UP*UP*UP*UP*UP*UP*UP*UP*UP*UP*UP*UP*UP*U)-3' |
|  | | |
| Cluster58 | NA | 4oi0_B: RNA (5'-R(*GP*C)-3') |
|  | | |
| Cluster19 | NA | 2xd0_G: TOXI |
|  |  | 2xd0_H: TOXI |
|  |  | 2xd0_I: TOXI |
|  |  | 2xd0_U: TOXI |
|  |  | 2xd0_V: TOXI |
|  |  | 2xd0_W: TOXI |
|  |  | 4ato_G: TOXI |
|  | | |
| Cluster46 | NA | 4d61_1: 18S RRNA |
|  |  | 3j7a_7: tRNA |
|  |  | 3j7a_A: 18S ribosomal RNA |
|  |  | 4kzx_i: 18S ribosomal RNA |
|  |  | 3j80_2: 18S rRNA |
|  |  | 4uer_A: 18S RRNA |
|  | | |
| Cluster96 | NA | 3j0e_A: ribosomal 23S RNA |
|  |  | 3j0e_e: ribosomal 16S RNA |
|  |  | 2om7_B: Fragment of 16S rRNA (h15) |
|  |  | 2om7_C: Fragment of 16S rRNA (h44) |
|  |  | 2om7_D: 16S ribosomal RNA (H5) |
|  |  | 2om7_F: Fragment of23S rRNA (H95) |
|  |  | 2om7_G: Fragment of23S rRNA (H68) |
|  |  | 2om7_H: Fragment of23S rRNA (H89) |
|  |  | 2om7_I: Fragment of23S rRNA (H42-44) |
|  |  | 2om7_J: Fragment of23S rRNA (H76) |
|  |  | 2om7_M: p/E-tRNA |
|  | | |
| Cluster15 | NA | 3j80_2: 18S rRNA |
|  |  | 3j16_J: 28S ribosomal RNA |
|  |  | 3j16_K: 18S ribosomal RNA |
|  |  | 3j16_L: P-site tRNA |
|  |  | 4kzx_i: 18S ribosomal RNA |
|  |  | 3j7a_7: tRNA |
|  |  | 3j7a_A: 18S ribosomal RNA |
|  |  | 4d61_1: 18S RRNA |
|  |  | 4d61_j: CRICKET PARALYSIS VIRUS IRES RNA |
|  | | |
| Cluster70 | NA | 2rkj_G: RNA (238-MER) |
|  |  | 2rkj_K: RNA (238-MER) |
|  | | |
| Cluster115 | NA | 2c4r_R: SSRNA MOLECULE: 5'-R(*AP*CP*AP*GP*UP*AP*UP*UP*UP*GP)-3' |
|  | | |
| Cluster118 | NA | 4ce4_A: 16S RRNA |
|  |  | 3j7y_A: 16S rRNA |
|  |  | 3j7y_B: mt-tRNAVal |
|  | | |
| Cluster101 | NA | 4wsb_R: Influenza A polymerase vRNA promoter 3' end |
|  |  | 4wsb_V: Influenza A polymerase vRNA promoter 5' end |
|  |  | 4wrt_R: Influenza virus polymerase vRNA promoter 3' end |
|  |  | 4wrt_V: Influenza virus polymerase vRNA promoter 5' end |
|  | | |
| Cluster24 | NA | 4s2x_B: RNA (5'-R(*(APC)*GP*U)-3') |
|  |  | 3j7y_B: mt-tRNAVal |
|  |  | 4jzv_C: RNA (5'-R(*(GCP)P*G)-3') |
|  | | |
| Cluster28 | NA | 3avw_G: RNA (5'-R(*GP*GP*GP*UP*CP*CP*AP*C)-3') |
|  |  | 3avw_T: RNA (5'-R(*AP*UP*CP*GP*UP*GP*GP*AP*CP*CP*CP*A |
|  | | |
| Cluster22 | NA | 3r2d_S: 5'-R(*GP*GP*CP*UP*CP*CP*UP*UP*GP*GP*CP*A)-3' |
|  |  | 4ftb_R: Flock House virus genomic RNA |
|  | | |
| Cluster21 | NA | 2du6_D: tRNA |
|  |  | 2du3_D: tRNA |
|  | | |
| Cluster105 | NA | 2i82_H: 5'- R(*GP*AP*GP*GP*GP*GP*AP*UP*UP*GP*AP*AP*AP*AP*UP*CP*CP*CP*CP *UP*C)-3' |
|  | | |
| Cluster97 | NA | 4afy_C: 5'-R(*GP*GP)-3' |
|  |  | 4afy_D: 5'-R(*GP*GP)-3' |
|  |  | 3u2e_D: RNA (5'-R(P*GP*G)-3') |
|  | | |
| Cluster61 | NA | 2rsk_A: RNA (5'-R(*GP*GP*AP*GP*GP*AP*GP*GP*AP*GP*GP*A)-3') |
|  |  | 2rsk_B: RNA (5'-R(*GP*GP*AP*GP*GP*AP*GP*GP*AP*GP*GP*A)-3') |
|  | | |
| Cluster114 | NA | 4by9_B: SSR26 |
|  |  | 4by9_Y: 5'-R(*UP*CP*GP*CP*CP*CP*AP*UP*CP*AP*CP)-3' |
|  |  | 4by9_Z: 5'-R(*UP*CP*GP*CP*CP*CP*AP*UP*CP*AP*CP)-3' |
|  | | |
| Cluster52 | NA | 4d61_1: 18S RRNA |
|  |  | 4d61_j: CRICKET PARALYSIS VIRUS IRES RNA |
|  |  | 3j81_2: 18S rRNA |
|  |  | 3j7a_A: 18S ribosomal RNA |
|  |  | 4uer_A: 18S RRNA |
|  |  | 4kzy_i: 18S Ribosomal RNA |
|  | | |
| Cluster90 | NA | 4u7u_L: crRNA |
|  | | |
| Cluster34 | NA | 2vrt_G: 5'-R(*UP*UP*GP)-3' |
|  | | |
| Cluster86 | NA | 4fvu_B: RNA (5'-R(*GP*GP*AP*GP*GP*GP*AP*G)-3') |
|  |  | 4fvu_C: RNA (5'-R(*CP*UP*CP*CP*CP*UP*CP*C)-3') |
|  |  | 4gv3_B: RNA (5'-R(*(GTP)P*GP*GP*C)-3') |
|  |  | 4gv3_C: RNA (5'-R(P*CP*GP*CP*CP*C)-3') |
|  | | |
| Cluster106 | NA | 4by9_A: SSR26 |
|  |  | 4by9_B: SSR26 |
|  |  | 4by9_W: 5'-R(*UP*CP*GP*CP*CP*CP*AP*UP*CP*AP*CP)-3' |
|  |  | 4by9_X: 5'-R(*UP*CP*GP*CP*CP*CP*AP*UP*CP*AP*CP)-3' |
|  |  | 4by9_Y: 5'-R(*UP*CP*GP*CP*CP*CP*AP*UP*CP*AP*CP)-3' |
|  |  | 4by9_Z: 5'-R(*UP*CP*GP*CP*CP*CP*AP*UP*CP*AP*CP)-3' |
|  | | |
| Cluster116 | NA | 3v11_D: Initiator tRNA |
|  | | |
| Cluster41 | NA | 3j0q_3: 60S ribosomal RNA fragment |
|  |  | 3j0q_9: 60S ribosomal RNA fragment |
|  |  | 3j0q_a: 40S ribosomal RNA fragment |
|  |  | 3j0q_h: 40S ribosomal RNA fragment |
|  |  | 3j0q_w: mRNA fragment |
|  |  | 3j0q_W: tRNA |
|  |  | 3j0q_y: mRNA fragment |
|  |  | 3j0q_Y: tRNA |
|  | | |
| Cluster80 | NA | 3wqy_C: RNA (75-MER) |
|  | | |
| Cluster72 | NA | 2ese_B: 5'- R(*GP*GP*AP*GP*AP*GP*GP*CP*UP*CP*UP*GP*GP*CP*AP*GP*CP*UP*UP *UP*UP*CP*C)-3' |
|  |  | 2b6g_B: 5'- R(*GP*GP*AP*GP*GP*CP*UP*CP*UP*GP*GP*CP*AP*GP*CP*UP*UP*UP*C) -3' |
|  | | |
| Cluster132 | NA | 4dwa_D: RNA (5'-R(*CP*UP*C)-3') |
|  | | |
| Cluster8 | NA | 3j81_1: Met-tRNAi |
|  |  | 3j81_2: 18S rRNA |
|  |  | 3j81_3: mRNA |
|  |  | 4kzx_i: 18S ribosomal RNA |
|  | | |
| Cluster91 | NA | 4wsa_R: Influenza B vRNA promoter 3' end |
|  |  | 4wsa_V: Influenza B vRNA promoter 5' end |
|  |  | 4wsb_R: Influenza A polymerase vRNA promoter 3' end |
|  |  | 4wsb_V: Influenza A polymerase vRNA promoter 5' end |
|  | | |
| Cluster77 | NA | 4kr7_M: RNA (39-MER) |
|  |  | 4kr7_X: RNA (39-MER) |
|  | | |
| Cluster130 | NA | 4erd_C: 5'-R(P*GP*GP*UP*CP*GP*AP*CP*AP*UP*CP*UP*UP*CP*GP*GP*AP*UP*GP*GP*AP*CP*C)-3' |
|  |  | 4erd_D: 5'-R(P*GP*GP*UP*CP*GP*AP*CP*AP*UP*CP*UP*UP*CP*GP*GP*AP*UP*GP*GP*AP*CP*C)-3' |
|  | | |
| Cluster6 | NA | 3j80_2: 18S rRNA |
|  |  | 3j7a_7: tRNA |
|  |  | 3j7a_A: 18S ribosomal RNA |
|  |  | 4kzx_i: 18S ribosomal RNA |
|  |  | 4uer_A: 18S RRNA |
|  | | |
| Cluster108 | NA | 2zni_C: bacterial tRNA |
|  |  | 2zni_D: bacterial tRNA |
|  | | |
| Cluster69 | NA | 3amu_B: RNA (78-MER) |
|  | | |
| Cluster65 | NA | 4wrt_R: Influenza virus polymerase vRNA promoter 3' end |
|  |  | 4wrt_V: Influenza virus polymerase vRNA promoter 5' end |
|  |  | 3j9b_D: RNA (5'-R(P*AP*AP*AP*AP*AP*A)-3') |
|  |  | 3j9b_E: RNA (5'-R(*UP*UP*UP*UP*UP*A)-3') |
|  |  | 4wsb_R: Influenza A polymerase vRNA promoter 3' end |
|  |  | 4wsb_V: Influenza A polymerase vRNA promoter 5' end |
|  | | |
| Cluster76 | NA | 3j7z_A: 23S rRNA |
|  |  | 4wwe_1: 23S RRNA (2899-MER) |
|  |  | 4wwe_s: 5S RRNA (119-MER) |
|  | | |
| Cluster84 | NA | 3w1k_F: selenocysteine tRNA |
|  |  | 3w1k_G: selenocysteine tRNA |
|  |  | 3w1k_H: selenocysteine tRNA |
|  |  | 3w1k_I: selenocysteine tRNA |
|  |  | 3w1k_J: selenocysteine tRNA |
|  | | |
| Cluster38 | NA | 4d61_1: 18S RRNA |
|  |  | 4d61_j: CRICKET PARALYSIS VIRUS IRES RNA |
|  |  | 3j81_1: Met-tRNAi |
|  |  | 3j81_2: 18S rRNA |
|  |  | 3j7a_7: tRNA |
|  |  | 3j7a_A: 18S ribosomal RNA |
|  |  | 4kzz_i: 18S Ribosomal RNA |
|  |  | 4uer_A: 18S RRNA |
|  | | |
| Cluster134 | NA | 4o8j_D: RNA |
|  |  | 4o8j_E: RNA |
|  | | |
| Cluster16 | NA | 4r3i_B: RNA (5'-R(*GP*GP*(6MZ)P*CP*U)-3') |
|  |  | 2mtv_B: RNA_(5'-R(*UP*GP*(6MZ)P*CP*AP*C)-3') |
|  |  | 4u8t_P: RNA (5'-R(*AP*GP*GP*(6MZ)P*CP*AP*U)-3') |
|  |  | 4u8t_Q: RNA (5'-R(*AP*GP*GP*(6MZ)P*CP*AP*U)-3') |
|  |  | 4u8t_R: RNA (5'-R(*AP*GP*GP*(6MZ)P*CP*AP*U)-3') |
|  |  | 4rcj_B: RNA (5'-R(*GP*GP*(6MZ)P*CP*U)-3') |
|  | | |
| Cluster120 | NA | 2wwa_D: 25S RRNA |
|  |  | 2wwa_E: 25S RRNA |
|  |  | 2wwa_F: 25S RRNA |
|  |  | 2wwa_G: 25S RRNA |
|  | | |
| Cluster79 | NA | 3t5q_D: RNA (5'-R(P*UP*AP*UP*CP*UP*CP*A)-3') |
|  |  | 3t5q_F: RNA (5'-R(P*UP*UP*AP*UP*CP*UP*CP*A)-3') |
|  |  | 3t5q_L: RNA (5'-R(P*UP*AP*UP*CP*UP*C)-3') |
|  |  | 3t5n_C: RNA (5'-R(P*UP*AP*UP*CP*UP*C)-3') |
|  | | |
| Cluster133 | NA | 4j7m_B: RNA (5'-R(P*UP*(U37)P*(U37)P*UP*U)-3') |
|  | | |
| Cluster111 | NA | 4ce4_A: 16S RRNA |
|  |  | 3j7y_A: 16S rRNA |
|  |  | 3j7y_B: mt-tRNAVal |
|  |  | 5aj3_A: MITORIBOSOMAL 12S RRNA |
|  | | |
| Cluster131 | NA | 3j7z_A: 23S rRNA |
|  | | |
| Cluster93 | NA | 4ce4_A: 16S RRNA |
|  |  | 3j7y_A: 16S rRNA |
|  |  | 3j7y_A: 16S rRNA |
|  |  | 5aj3_A: MITORIBOSOMAL 12S RRNA |
|  | | |
| Cluster107 | NA | 4ce4_A: 16S RRNA |
|  |  | 3j7y_A: 16S rRNA |
|  | | |
| Cluster119 | NA | 4d61_1: 18S RRNA |
|  |  | 4d61_j: CRICKET PARALYSIS VIRUS IRES RNA |
|  | | |
| Cluster14 | NA | 3dh3_E: stem loop fragment of E. Coli 23S RNA |
|  |  | 3dh3_F: stem loop fragment of E. Coli 23S RNA |
|  |  | 3dh3_G: stem loop fragment of E. Coli 23S RNA |
|  |  | 4lgt_E: stem-loop of 23S rRNA |
|  |  | 4lgt_F: stem-loop of 23S rRNA |
|  | | |
| Cluster9 | NA | 3wc1_P: 75-mer tRNA |
|  |  | 3wc1_Q: 75-mer tRNA |
|  | | |
| Cluster59 | NA | 3j81_2: 18S rRNA |
|  |  | 3j81_3: mRNA |
|  |  | 4kzx_i: 18S ribosomal RNA |
|  |  | 4uer_A: 18S RRNA |
|  |  | 3j7a_7: tRNA |
|  |  | 3j7a_A: 18S ribosomal RNA |
|  | | |
| Cluster3 | NA | 3j7y_A: 16S rRNA |
|  |  | 3j7y_B: mt-tRNAVal |
|  |  | 4ce4_A: 16S RRNA |
|  |  | 4ce4_B: UNASSIGNED RNA |
|  |  | 3j80_2: 18S rRNA |
|  |  | 3j7a_A: 18S ribosomal RNA |
|  |  | 4kzz_i: 18S Ribosomal RNA |
|  |  | 3j16_J: 28S ribosomal RNA |
|  |  | 3j16_K: 18S ribosomal RNA |
|  |  | 3j7y_B: mt-tRNAVal |
|  |  | 5aj3_A: MITORIBOSOMAL 12S RRNA |
|  | | |
| Cluster124 | NA | 3zc0_N: 5'-R(*UP*UP*CP*GP*AP*CP*GP*CP*GP*UP*CP*GP*AP*AP*UP*U)-3' |
|  |  | 3zc0_O: 5'-R(*UP*UP*CP*GP*AP*CP*GP*CP*GP*UP*CP*GP*AP*AP*UP*U)-3' |
|  | | |
| Cluster68 | NA | 4b3g_G: RNA (5'-(AP*AP*AP*AP*AP*AP*AP*AP*AP)-3') |
|  |  | 4b3g_H: RNA (5'-(AP*AP*AP*AP*AP*AP*AP*AP*AP)-3') |
|  |  | 2xzo_D: 5'-R(*UP*UP*UP*UP*UP*UP*UP)-3' |
|  |  | 2xzl_B: 5- R(*UP*UP*UP*UP*UP*UP*UP*UP*U) -3 |
|  | | |
| Cluster27 | NA | 2a1r_C: 5'-R(*AP*AP*A)-3' |
|  |  | 2a1r_D: 5'-R(*AP*AP*A)-3' |
|  | | |
| Cluster123 | NA | 4pmi_A: Rev-Response-Element RNA |
|  |  | 3j0d_A: ribosomal 23S RNA |
|  |  | 3j0d_B: ribosomal 23S RNA |
|  |  | 3j0d_C: ribosomal 23S RNA |
|  |  | 3j0d_E: ribosomal 23S RNA |
|  |  | 3j0d_F: ribosomal 23S RNA |
|  |  | 3j0d_H: ribosomal 16S RNA |
|  | | |
| Cluster36 | NA | 4wj3_R: 76mer-tRNA |
|  |  | 4wj3_T: 76mer-tRNA |
|  |  | 3kfu_K: tRNA-Asn |
|  |  | 3kfu_M: tRNA-Asn |
|  |  | 3kfu_N: tRNA-Asn |
|  | | |
| Cluster104 | NA | 4wwt_s: THERMUS THERMOPHILUS HB8 GENOMIC DNA, COMPLETE GENOME |
|  | | |
| Cluster50 | NA | 4al5_B: 5'-R(*UP*UP*CP*AP*CP*UP*GP*CP*CP*GP*UP*AP*UP*AP *GP*GP*CP*AP*GP*C)-3' |
|  |  | 2xli_B: 5'-R(*CP*UP*GP*CP*CP*GP*UP*AP*UP*AP*GP*GP*CP*A*DG*C)-3' |
|  |  | 2xlk_C: 5'-R(*CP*UP*GP*CP*CP*GP*UP*AP*UP*AP*GP*GP*CP*A*DG*C)-3' |
|  |  | 2xlk_D: 5'-R(*CP*UP*GP*CP*CP*GP*UP*AP*UP*AP*GP*GP*CP*A*DG*C)-3' |
|  |  | 4al7_B: 5'-R(*CP*UP*GP*CP*CP*GP*UP*AP*UP*AP*GP*GP*CP*AP*G)-3' |
|  |  | 4al6_B: CRRNA, 5'-R(CP*UP*GP*CP*CP*GP*UP*AP*UP*AP*GP*GP* CP*AP*GP*C)-3' |
|  | | |
| Cluster126 | NA | 3j3w_A: ribosome RNA 23S |
|  | | |
| Cluster122 | NA | 3q1r_B: RNase P RNA |
|  |  | 3q1r_D: TRNA 5' LEADER |
|  | | |
| Cluster23 | NA | 4tuw_C: HISTONE MRNA 3' STEM LOOP |
|  |  | 4tuw_D: HISTONE MRNA 3' STEM LOOP |
|  |  | 4l8r_A: HISTONE MRNA STEM-LOOP |
|  |  | 4tv0_B: RNA (26-MER) |
|  |  | 4tux_C: RNA (26-MER) |
|  |  | 4tux_D: RNA (26-MER) |
|  | | |
| Cluster64 | NA | 4kzy_i: 18S Ribosomal RNA |
|  |  | 4uer_A: 18S RRNA |
|  |  | 3j81_1: Met-tRNAi |
|  |  | 3j81_2: 18S rRNA |
|  |  | 3j81_3: mRNA |
|  | | |
| Cluster129 | NA | 4ce4_A: 16S RRNA |
|  |  | 3j7y_A: 16S rRNA |
|  | | |
| Cluster12 | NA | 3cz3_E: RNA (5'- R(P*CP*GP*UP*AP*CP*GP*CP*GP*GP*AP*AP*UP*AP*CP*UP*UP*CP*GP*A )-3') |
|  |  | 3cz3_F: RNA (5'- R(P*UP*CP*GP*AP*AP*GP*UP*AP*UP*UP*CP*CP*GP*CP*GP*UP*AP*CP*G )-3') |
|  |  | 3cz3_G: RNA (5'- R(P*CP*GP*UP*AP*CP*GP*CP*GP*GP*AP*AP*UP*AP*CP*UP*UP*CP*GP*A )-3') |
|  |  | 3cz3_H: RNA (5'- R(P*UP*CP*GP*AP*AP*GP*UP*AP*UP*UP*CP*CP*GP*CP*GP*UP*AP*CP*G )-3') |
|  |  | 2zi0_C: RNA (5'- D(P*AP*GP*AP*CP*AP*GP*CP*AP*UP*UP*AP*UP*GP*CP*UP*GP*UP*CP*UP*UP*U)- 3') |
|  |  | 2zi0_D: RNA (5'- D(P*AP*GP*AP*CP*AP*GP*CP*AP*UP*UP*AP*UP*GP*CP*UP*GP*UP*CP*UP*UP*U)- 3') |
|  | | |
| Cluster44 | NA | 4d61_1: 18S RRNA |
|  |  | 4d61_j: CRICKET PARALYSIS VIRUS IRES RNA |
|  |  | 3j81_1: Met-tRNAi |
|  |  | 3j81_2: 18S rRNA |
|  |  | 3j81_3: mRNA |
|  |  | 3j7a_7: tRNA |
|  |  | 3j7a_A: 18S ribosomal RNA |
|  |  | 4uer_A: 18S RRNA |
|  | | |
| Cluster87 | NA | 4pjo_3: U1 RNA variant (48-MER) with 4-helix junction |
|  |  | 4pjo_4: U1 RNA variant (48-MER) with 4-helix junction |
|  |  | 4pjo_y: RNA (5'-R(*AP*GP*GP*UP*AP*AP*GP*UP*CP*C)-3') |
|  |  | 4pjo_Y: RNA (5'-R(*AP*GP*GP*UP*AP*AP*GP*UP*CP*C)-3') |
|  |  | 3cw1_V: U1 snRNA |
|  |  | 3cw1_w: U1 snRNA |
|  | | |
| Cluster73 | NA | 4d5l_1: 18S RRNA 2 |
|  |  | 3j7a_A: 18S ribosomal RNA |
|  | | |
| Cluster135 | NA | 4oog_D: 34-mer RNA |
|  | | |
| Cluster99 | NA | 4d61_1: 18S RRNA |
|  |  | 4d61_j: CRICKET PARALYSIS VIRUS IRES RNA |
|  |  | 3j81_2: 18S rRNA |
|  |  | 3j7a_A: 18S ribosomal RNA |
|  |  | 4kzy_i: 18S Ribosomal RNA |
|  |  | 4uer_A: 18S RRNA |
|  | | |
| Cluster10 | NA | 4d5l_1: 18S RRNA 2 |
|  |  | 3j80_2: 18S rRNA |
|  |  | 3uzs_C: C13.28 RNA Aptamer |
|  |  | 1trj_C: Helix 40 of 18S rRNA |
|  |  | 4uer_A: 18S RRNA |
|  | | |
| Cluster51 | NA | 4l8r_A: HISTONE MRNA STEM-LOOP |
|  |  | 4l8r_D: HISTONE MRNA STEM-LOOP |
|  |  | 1zbh_E: 5'-R(*CP*CP*GP*GP*CP*UP*CP*UP*UP*UP*UP*CP*AP* |
|  | | |
| Cluster75 | NA | 4g0a_G: RNA (5'-R(P*GP*GP*U)-3') |
|  |  | 4g0a_H: RNA (5'-R(P*GP*GP*U)-3') |
|  | | |
| Cluster83 | NA | 2w2h_S: 5'-R(*GP*CP*UP*CP*AP*GP*AP*UP*CP*UP *GP*CP*GP*GP*UP*CP*UP*GP*AP*GP*C)-3' |
|  | | |
| Cluster31 | NA | 3uzt_B: C13.18 RNA Aptamer |
|  | | |
| Cluster4 | NA | 4tu0_F: 2'-5' OLIGOADENYLATE TRIMER |
|  | | |
| Cluster113 | NA | 5aka_B: 23S RNA |
|  | | |
| Cluster66 | NA | 4kzy_i: 18S Ribosomal RNA |
|  |  | 3j80_2: 18S rRNA |
|  |  | 3j7a_A: 18S ribosomal RNA |
|  |  | 4uer_A: 18S RRNA |
|  | | |
| Cluster37 | NA | 4kzy_i: 18S Ribosomal RNA |
|  |  | 4d5l_1: 18S RRNA 2 |
|  |  | 3j7a_7: tRNA |
|  |  | 3j7a_A: 18S ribosomal RNA |
|  |  | 3j81_1: Met-tRNAi |
|  |  | 3j81_2: 18S rRNA |
|  |  | 3j81_3: mRNA |
|  |  | 4uer_A: 18S RRNA |
|  | | |
| Cluster11 | NA | 3j7a_7: tRNA |
|  |  | 3j7a_A: 18S ribosomal RNA |
|  |  | 1ysh_A: RNA (28-MER) |
|  |  | 1ysh_B: RNA (101-MER) |
|  |  | 3j80_2: 18S rRNA |
|  |  | 4hor_X: RNA (5'-R(*(CTP)P*CP*CP*CP*C)-3') |
|  |  | 4kzy_i: 18S Ribosomal RNA |
|  |  | 4uer_A: 18S RRNA |
|  | | |
| Cluster17 | NA | 4uer_A: 18S RRNA |
|  |  | 4kzy_i: 18S Ribosomal RNA |
|  |  | 3j81_1: Met-tRNAi |
|  |  | 3j81_2: 18S rRNA |
|  |  | 3j81_3: mRNA |
|  | | |
| Cluster47 | NA | 4pjo_3: U1 RNA variant (48-MER) with 4-helix junction |
|  | | |
| Cluster95 | NA | 4kzz_i: 18S Ribosomal RNA |
|  |  | 3j81_2: 18S rRNA |
|  |  | 3j81_3: mRNA |
|  |  | 4uer_A: 18S RRNA |
|  |  | 3j7a_A: 18S ribosomal RNA |
|  | | |
| Cluster29 | NA | 2det_C: tRNA |
|  | | |
| Cluster40 | NA | 4csu_B: 23S RRNA |
|  | | |
| Cluster30 | NA | 4x9e_H: RNA (5'-R(P*CP*CP*C)-3') |
|  | | |
| Cluster32 | NA | 4h5o_L: 35-mer poly(U) RNA |
|  |  | 4csf_g: RNA (5'-R(*UP*GP*UP*GP*UP*UP*UP*CP*UP)-3') |
|  |  | 4csf_i: RNA (5'-R(*UP*GP*UP*GP*UP*UP*UP*CP*UP)-3') |
|  |  | 4csf_k: RNA (5'-R(*UP*GP*UP*GP*UP*UP*UP*CP*UP)-3') |
|  |  | 4csf_s: RNA (5'-R(*UP*GP*UP*GP*UP*UP*UP*CP*UP)-3') |
|  |  | 4csf_u: RNA (5'-R(*UP*GP*UP*GP*UP*UP*UP*CP*UP)-3') |
|  |  | 4csf_w: RNA (5'-R(*UP*GP*UP*GP*UP*UP*UP*CP*UP)-3') |
|  |  | 4csf_a: RNA (5'-R(*UP*GP*UP*GP*UP*UP*UP*CP*UP)-3') |
|  |  | 4csf_c: RNA (5'-R(*UP*GP*UP*GP*UP*UP*UP*CP*UP)-3') |
|  |  | 4csf_m: RNA (5'-R(*UP*GP*UP*GP*UP*UP*UP*CP*UP)-3') |
|  |  | 4csf_o: RNA (5'-R(*UP*GP*UP*GP*UP*UP*UP*CP*UP)-3') |
|  |  | 4csf_a: RNA (5'-R(*UP*GP*UP*GP*UP*UP*UP*CP*UP)-3') |
|  |  | 4csf_c: RNA (5'-R(*UP*GP*UP*GP*UP*UP*UP*CP*UP)-3') |
|  |  | 4csf_e: RNA (5'-R(*UP*GP*UP*GP*UP*UP*UP*CP*UP)-3') |
|  |  | 4csf_m: RNA (5'-R(*UP*GP*UP*GP*UP*UP*UP*CP*UP)-3') |
|  |  | 4csf_o: RNA (5'-R(*UP*GP*UP*GP*UP*UP*UP*CP*UP)-3') |
|  | | |
| Cluster100 | NA | 4lj0_C: polyadenosine RNA |
|  |  | 4lj0_D: polyadenosine RNA |
|  | | |
| Cluster60 | NA | 3oij_C: 5'-R(*GP*GP*GP*CP*UP*UP*CP*AP*AP*CP*GP*CP*CP*C)-3' |
|  |  | 3oij_D: 5'-R(*GP*GP*GP*CP*UP*UP*CP*AP*AP*CP*GP*CP*CP*C)-3' |
|  | | |
| Cluster25 | NA | 2zxu_C: tRNA(Phe) |
|  |  | 2zxu_D: tRNA(Phe) |
|  |  | 3eph_E: tRNA |
|  |  | 3eph_F: tRNA |
|  | | |
| Cluster54 | NA | 4bbl_Y: RNA |
|  |  | 4bbl_Z: RNA |
|  | | |
| Cluster71 | NA | 2zzn_C: RNA (71-MER) |
|  |  | 2zzn_D: RNA (71-MER) |
|  | | |
| Cluster18 | NA | 2wwb_D: 5.8S RRNA |
|  |  | 2wwb_E: 25S RRNA |
|  |  | 2wwb_F: 25S RRNA |
|  |  | 2wwb_G: 25S RRNA |
|  |  | 2wwa_D: 25S RRNA |
|  |  | 2wwa_E: 25S RRNA |
|  |  | 2wwa_F: 25S RRNA |
|  |  | 2wwa_G: 25S RRNA |
|  |  | 3j45_1: 23S ribosomal RNA |
|  |  | 3j45_2: 23S ribosomal RNA |
|  |  | 3j45_5: 23S ribosomal RNA |
|  |  | 3bo1_E: 23S RIBOSOMAL RNA |
|  |  | 3bo1_G: 23S RIBOSOMAL RNA |
|  |  | 3dkn_D: RNA (5'- R(P*CP*GP*UP*GP*CP*CP*AP*AP*GP*CP*UP*GP*CP*GP*AP*UP*AP*AP*G P*C)-3') |
|  |  | 3dkn_E: RNA (5'- R(P*AP*GP*CP*CP*GP*CP*AP*CP*GP*GP*AP*GP*GP*CP*GP*AP*A)-3') |
|  |  | 3dkn_F: RNA (32-MER) |
|  | | |
| Cluster43 | NA | 4db2_E: 5'-R(*GP*GP*GP*CP*GP*GP*GP*CP*CP*CP*GP*CP*CP*C)-3' |
|  |  | 4db2_F: 5'-R(*GP*GP*GP*CP*GP*GP*GP*CP*CP*CP*GP*CP*CP*C)-3' |
|  |  | 4db2_G: 5'-R(*GP*GP*GP*CP*GP*GP*GP*CP*CP*CP*GP*CP*CP*C)-3' |
|  |  | 4db2_I: 5'-R(*GP*GP*GP*CP*GP*GP*GP*CP*CP*CP*GP*CP*CP*C)-3' |
|  |  | 4db2_J: 5'-R(*GP*GP*GP*CP*GP*GP*GP*CP*CP*CP*GP*CP*CP*C)-3' |
|  | | |
| Cluster109 | NA | 4n2s_B: Zm1a-6 RNA |
|  | | |
| Cluster102 | NA | 4cxh_1: 18S RRNA - H44 |
|  |  | 4cxh_a: 18S RRNA - H5-H14 |
|  |  | 4cxh_c: 28S RRNA - H95 |
|  |  | 4cxh_x: MESSENGER RNA |
|  |  | 4cxh_Y: TRANSFER RNA |
|  | | |
| Cluster57 | NA | 4qik_C: 5'-R(*AP*C*AP*UP*GP*UP*UP*UP*UP*CP*UP*GP*UP*GP*AP*AP*AP*AP*CP*GP*GP*AP*G)-3' |
|  |  | 4qik_D: 5'-R(*AP*C*AP*UP*GP*UP*UP*UP*UP*CP*UP*GP*UP*GP*AP*AP*AP*AP*CP*GP*GP*AP*G)-3' |
|  | | |
| Cluster121 | NA | 4u7u_L: crRNA |
|  |  | 4u7u_X: crRNA |
|  | | |
| Cluster81 | NA | 2w2h_S: 5'-R(*GP*CP*UP*CP*AP*GP*AP*UP*CP*UP *GP*CP*GP*GP*UP*CP*UP*GP*AP*GP*C)-3' |
|  | | |
| Cluster7 | NA | 4bkk_A: RNA (161-MER) |
|  |  | 4xjn_N: RNA (78-MER) |
|  |  | 4uft_R: 5'-R(*CP*CP*CP*CP*CP*CP)-3' |
|  | | |
| Cluster112 | NA | 4u7u_L: crRNA |
|  |  | 4u7u_X: crRNA |
|  | | |
| Cluster56 | NA | 4yco_D: tRNAphe |
|  |  | 4yco_E: tRNAphe |
|  |  | 4yco_F: tRNAphe |
|  |  | 3b0u_A: RNA (5'-R(*GP*GP*(H2U)P*A)-3') |
|  |  | 3b0u_B: RNA (5'-R(*GP*GP*(H2U)P*A)-3') |
|  |  | 3b0v_B: tRNA |
|  | | |
| Cluster39 | NA | 4pei_V: RNA (5'-R(*UP*AP*AP*CP*A)-3') |
|  |  | 4pei_Z: RNA (5'-R(*UP*AP*AP*CP*A)-3') |
|  | | |
| Cluster13 | NA | 4c8y_C: R1 REPEAT RNA SUBSTRATE MIMIC |
|  |  | 3pkm_G: 5'-R(*AP*UP*UP*AP*CP*AP*AP*UP*AP*A)-3' |
|  |  | 3pkm_R: 5'-R(P*UP*UP*AP*CP*AP*AP*UP*AP*A)-3' |
|  |  | 4ilm_C: RNA (5'-R(*GP*CP*UP*AP*AP*UP*CP*UP*AP*CP*UP*AP*UP*AP*GP*A)-3') |
|  |  | 4ilm_I: RNA (5'-R(*GP*CP*UP*AP*AP*UP*CP*UP*AP*CP*UP*AP*UP*AP*GP*A)-3') |
|  |  | 4ilm_R: RNA (5'-R(*GP*CP*UP*AP*AP*UP*CP*UP*AP*CP*UP*AP*UP*AP*GP*A)-3') |
|  |  | 4c9d_C: R3 REPEAT RNA CLEAVAGE PRODUCT |
|  |  | 4c9d_D: R3 REPEAT RNA CLEAVAGE PRODUCT |
|  |  | 3qjl_R: RNA (5'-R(*GP*UP*UP*AP*CP*AP*AP*UP*AP*AP*GP*A)-3') |
|  |  | 3qjl_X: RNA (5'-R(*GP*UP*UP*AP*CP*AP*AP*UP*AP*AP*GP*A)-3') |
|  | | |
| Orphan1 | NA | 1f8v_R: RNA |
|  | | |
| Orphan2 | NA | 1f8v_R: RNA |
|  | | |
| Orphan3 | NA | 1zbn_A: BIV mRNA |
|  | | |
| Orphan4 | NA | 1zo1_F: P/I-site tRNA |
|  | | |
| Orphan5 | NA | 2a9x_2: BIV TAR RNA |
|  | | |
| Orphan6 | NA | 2aar_0: 23S ribosomal RNA |
|  | | |
| Orphan7 | NA | 2d3o_0: 23S RIBOSOMAL RNA |
|  | | |
| Orphan8 | NA | 2ftc_R: Mitochondrial 16S ribosomal RNA |
|  | | |
| Orphan9 | NA | 2ftc_R: Mitochondrial 16S ribosomal RNA |
|  | | |
| Orphan10 | NA | 2ftc_R: Mitochondrial 16S ribosomal RNA |
|  | | |
| Orphan11 | NA | 2ihx_B: uPsi RNA |
|  | | |
| Orphan12 | NA | 2jq7_B: RIBOSOMAL RNA |
|  | | |
| Orphan13 | NA | 2kdq_B: HIV-1 TAR RNA |
|  | | |
| Orphan14 | NA | 2kg1_B: 5'-R(*AP*GP*GP*GP*AP*U)-3' |
|  | | |
| Orphan15 | NA | 2kx5_A: HIV TAR RNA |
|  | | |
| Orphan16 | NA | 2l41_B: RNA (5'-R(P*UP*CP*UP*U)-3') |
|  | | |
| Orphan17 | NA | 2la5_A: RNA (36-MER) |
|  | | |
| Orphan18 | NA | 2li8_B: RNA (5'-R(*AP*GP*GP*AP*GP*AP*U)-3') |
|  | | |
| Orphan19 | NA | 2mkk_B: RNA (5'-R(*UP*UP*UP*UP*A)-3') |
|  | | |
| Orphan20 | NA | 2mkn_B: RNA (5'- R(*GP*CP*CP*GP*UP*GP*GP*UP*CP*UP*GP*GP*UP*GP*GP*CP*CP*GP*G)-3') |
|  |  | 2mkn_C: RNA (5'- R(P*CP*CP*GP*GP*CP*CP*AP*CP*CP*AP*GP*AP*CP*CP*AP*CP*GP*GP*C)-3') |
|  | | |
| Orphan21 | NA | 2mqv_B: RNA (68-MER) |
|  | | |
| Orphan22 | NA | 2ms0_B: tRNApro |
|  | | |
| Orphan23 | NA | 2ms0_B: tRNApro |
|  | | |
| Orphan24 | NA | 2ms1_B: tRNApro |
|  | | |
| Orphan25 | NA | 2qqp_R: RNA (5'-R(*UP*UP*UP*U)-3') |
|  | | |
| Orphan26 | NA | 2r8s_R: P4-P6 RNA RIBOZYME DOMAIN |
|  | | |
| Orphan27 | NA | 2ru7_A: RNA_(5'-R(*GP*GP*AP*GP*GP*AP*GP*GP*AP*GP*GP*A)-3') |
|  |  | 2ru7_B: RNA_(5'-R(*GP*GP*AP*GP*GP*AP*GP*GP*AP*GP*GP*A)-3') |
|  | | |
| Orphan28 | NA | 2ru7_A: RNA_(5'-R(*GP*GP*AP*GP*GP*AP*GP*GP*AP*GP*GP*A)-3') |
|  |  | 2ru7_B: RNA_(5'-R(*GP*GP*AP*GP*GP*AP*GP*GP*AP*GP*GP*A)-3') |
|  | | |
| Orphan29 | NA | 2wwa_D: 25S RRNA |
|  |  | 2wwa_E: 25S RRNA |
|  |  | 2wwa_F: 25S RRNA |
|  |  | 2wwa_G: 25S RRNA |
|  | | |
| Orphan30 | NA | 2x7n_A: SARCIN-RICIN LOOP |
|  | | |
| Orphan31 | NA | 2xc7_P: 5'-(AP*UP*CP*GP)-3' |
|  | | |
| Orphan32 | NA | 2ykr_A: 16S RRNA |
|  | | |
| Orphan33 | NA | 2ykr_A: 16S RRNA |
|  | | |
| Orphan34 | NA | 2ykr_A: 16S RRNA |
|  | | |
| Orphan35 | NA | 2ykr_A: 16S RRNA |
|  | | |
| Orphan36 | NA | 2ykr_A: 16S RRNA |
|  | | |
| Orphan37 | NA | 2ykr_A: 16S RRNA |
|  | | |
| Orphan38 | NA | 3a6p_D: pre-microRNA |
|  |  | 3a6p_E: pre-microRNA |
|  |  | 3a6p_I: pre-microRNA |
|  | | |
| Orphan39 | NA | 3ciy_C: 46-MER |
|  |  | 3ciy_D: 46-MER |
|  | | |
| Orphan40 | NA | 3dd2_B: RNA (26-MER) |
|  | | |
| Orphan41 | NA | 3g9y_C: RNA (5'-R(*AP*GP*GP*UP*AP*A)-3') |
|  | | |
| Orphan42 | NA | 3izy_N: tRNA-Phe |
|  | | |
| Orphan43 | NA | 3j0e_A: ribosomal 23S RNA |
|  |  | 3j0e_B: ribosomal 23S RNA |
|  |  | 3j0e_C: ribosomal 23S RNA |
|  |  | 3j0e_D: ribosomal 23S RNA |
|  |  | 3j0e_e: ribosomal 16S RNA |
|  | | |
| Orphan44 | NA | 3j16_J: 28S ribosomal RNA |
|  |  | 3j16_K: 18S ribosomal RNA |
|  |  | 3j16_L: P-site tRNA |
|  | | |
| Orphan45 | NA | 3j16_J: 28S ribosomal RNA |
|  |  | 3j16_K: 18S ribosomal RNA |
|  |  | 3j16_L: P-site tRNA |
|  | | |
| Orphan46 | NA | 3j16_J: 28S ribosomal RNA |
|  |  | 3j16_K: 18S ribosomal RNA |
|  |  | 3j16_L: P-site tRNA |
|  | | |
| Orphan47 | NA | 3j16_J: 28S ribosomal RNA |
|  |  | 3j16_K: 18S ribosomal RNA |
|  |  | 3j16_L: P-site tRNA |
|  | | |
| Orphan48 | NA | 3j3v_A: ribosome RNA 23S |
|  | | |
| Orphan49 | NA | 3j3v_A: ribosome RNA 23S |
|  | | |
| Orphan50 | NA | 3j3v_A: ribosome RNA 23S |
|  |  | 3j3v_B: ribosome RNA 5S |
|  | | |
| Orphan51 | NA | 3j3v_A: ribosome RNA 23S |
|  | | |
| Orphan52 | NA | 3j3v_A: ribosome RNA 23S |
|  | | |
| Orphan53 | NA | 3j3w_A: ribosome RNA 23S |
|  | | |
| Orphan54 | NA | 3j3w_A: ribosome RNA 23S |
|  | | |
| Orphan55 | NA | 3j3w_A: ribosome RNA 23S |
|  | | |
| Orphan56 | NA | 3j46_2: 23S ribosomal RNA |
|  |  | 3j46_4: 23S ribosomal RNA |
|  |  | 3j46_a: A-tRNA |
|  | | |
| Orphan57 | NA | 3j46_1: 23S ribosomal RNA |
|  |  | 3j46_2: 23S ribosomal RNA |
|  | | |
| Orphan58 | NA | 3j46_2: 23S ribosomal RNA |
|  |  | 3j46_4: 23S ribosomal RNA |
|  |  | 3j46_a: A-tRNA |
|  |  | 3j46_p: P-tRNA |
|  | | |
| Orphan59 | NA | 3j46_1: 23S ribosomal RNA |
|  |  | 3j46_2: 23S ribosomal RNA |
|  |  | 3j46_3: 23S ribosomal RNA |
|  |  | 3j46_4: 23S ribosomal RNA |
|  | | |
| Orphan60 | NA | 3j5s_A: 23S ribosomal RNA |
|  |  | 3j5s_B: 16S ribosomal RNA |
|  |  | 3j5s_E: P-site tRNA FMet |
|  | | |
| Orphan61 | NA | 3j7a_A: 18S ribosomal RNA |
|  | | |
| Orphan62 | NA | 3j7y_A: 16S rRNA |
|  | | |
| Orphan63 | NA | 3j7y_A: 16S rRNA |
|  | | |
| Orphan64 | NA | 3j7y_A: 16S rRNA |
|  | | |
| Orphan65 | NA | 3j7y_A: 16S rRNA |
|  |  | 3j7y_B: mt-tRNAVal |
|  | | |
| Orphan66 | NA | 3j7y_A: 16S rRNA |
|  | | |
| Orphan67 | NA | 3j7y_A: 16S rRNA |
|  | | |
| Orphan68 | NA | 3j7y_A: 16S rRNA |
|  |  | 3j7y_B: mt-tRNAVal |
|  | | |
| Orphan69 | NA | 3j7y_A: 16S rRNA |
|  | | |
| Orphan70 | NA | 3j7y_A: 16S rRNA |
|  | | |
| Orphan71 | NA | 3j7y_A: 16S rRNA |
|  | | |
| Orphan72 | NA | 3j7y_A: 16S rRNA |
|  |  | 3j7y_B: mt-tRNAVal |
|  | | |
| Orphan73 | NA | 3j7z_7: P-tRNA CCA-end |
|  |  | 3j7z_A: 23S rRNA |
|  | | |
| Orphan74 | NA | 3j81_1: Met-tRNAi |
|  |  | 3j81_2: 18S rRNA |
|  |  | 3j81_3: mRNA |
|  | | |
| Orphan75 | NA | 3j8g_B: 23S rRNA |
|  | | |
| Orphan76 | NA | 3j8g_B: 23S rRNA |
|  | | |
| Orphan77 | NA | 3j8g_B: 23S rRNA |
|  | | |
| Orphan78 | NA | 3kjo_B: DNA/RNA (5'-D(*T)-R(P*U)- D(P*AP*GP*GP*GP*TP*TP*AP*G)-3') |
|  | | |
| Orphan79 | NA | 3kyl_E: DNA/RNA (5'-R(*CP*UP*GP*AP*CP*CP*UP*GP*AP*C)- D(P*TP*TP*CP*GP*GP*TP*CP*AP*GP*GP*TP*CP*AP*G)-3') |
|  | | |
| Orphan80 | NA | 3lob_R: RNA (5'-R(*UP*UP*U*AP*UP*CP*UP*(P))-3') |
|  | | |
| Orphan81 | NA | 3o8c_C: RNA (5'-R(P*UP*(5BU)P*UP*UP*UP*U)-3') |
|  | | |
| Orphan82 | NA | 3qsy_D: tRNA |
|  | | |
| Orphan83 | NA | 3rc8_E: RNA fragment |
|  | | |
| Orphan84 | NA | 3s4g_C: RNA (5'-R(P*UP*UP*UP*U)-3') |
|  | | |
| Orphan85 | NA | 3tup_T: Thermus thermophilus tRNAPhe |
|  | | |
| Orphan86 | NA | 3uzs_C: C13.28 RNA Aptamer |
|  | | |
| Orphan87 | NA | 3zn8_G: 4.5 S RNA |
|  | | |
| Orphan88 | NA | 4a2i_A: 16S RIBOSOMAL RNA |
|  | | |
| Orphan89 | NA | 4adx_0: 23S RRNA |
|  |  | 4adx_8: 23S RRNA EXPANSION SEGMENTS |
|  | | |
| Orphan90 | NA | 4adx_0: 23S RRNA |
|  | | |
| Orphan91 | NA | 4adx_0: 23S RRNA |
|  | | |
| Orphan92 | NA | 4adx_0: 23S RRNA |
|  | | |
| Orphan93 | NA | 4csu_B: 23S RRNA |
|  | | |
| Orphan94 | NA | 4csu_B: 23S RRNA |
|  | | |
| Orphan95 | NA | 4csu_B: 23S RRNA |
|  | | |
| Orphan96 | NA | 4csu_B: 23S RRNA |
|  | | |
| Orphan97 | NA | 4csu_A: 5S RRNA |
|  |  | 4csu_B: 23S RRNA |
|  | | |
| Orphan98 | NA | 4d5n_X: CRICKET PARALYSIS VIRUS IRES RNA |
|  | | |
| Orphan99 | NA | 4f02_E: RNA (5'-R(*AP*AP*AP*AP*AP*AP*AP*AP*AP*AP*A)-3') |
|  | | |
| Orphan100 | NA | 4i67_B: 5'-R(P*GP*GP*GP*(RPC))-3' |
|  | | |
| Orphan101 | NA | 4ii9_C: RNA (5'-R(P*CP*CP*(A9Z))-3') |
|  | | |
| Orphan102 | NA | 4ioc_X: 23S ribosomal RNA |
|  |  | 4ioc_Y: 5S ribosomal RNA |
|  | | |
| Orphan103 | NA | 4oo1_S: POLY A RNA |
|  | | |
| Orphan104 | NA | 4ox9_A: 16S rRNA |
|  | | |
| Orphan105 | NA | 4p3e_A: Human 7S L gene, complete |
|  | | |
| Orphan106 | NA | 4rdx_C: tRNA(his) |
|  | | |
| Orphan107 | NA | 4uer_A: 18S RRNA |
|  | | |
| Orphan108 | NA | 4uer_A: 18S RRNA |
|  | | |
| Orphan109 | NA | 4uy8_A: RRNA-23S RIBOSOMAL RNA |
|  |  | 4uy8_V: RNA |
|  | | |
| Orphan110 | NA | 4wwt_1: 23S RRNA (2899-MER) |
|  |  | 4wwt_s: THERMUS THERMOPHILUS HB8 GENOMIC DNA, COMPLETE GENOME |
|  | | |
| Orphan111 | NA | 5aj3_A: MITORIBOSOMAL 12S RRNA |
|  | | |
| Orphan112 | NA | 5aj3_A: MITORIBOSOMAL 12S RRNA |
|  | | |
| Orphan113 | NA | 5aj3_A: MITORIBOSOMAL 12S RRNA |
|  | | |
| Orphan114 | NA | 5aj3_A: MITORIBOSOMAL 12S RRNA |
|  | | |
| Orphan115 | NA | 5aj3_A: MITORIBOSOMAL 12S RRNA |
|  | | |
| Orphan116 | NA | 5aj3_A: MITORIBOSOMAL 12S RRNA |
|  | | |
| Orphan117 | NA | 5aj3_A: MITORIBOSOMAL 12S RRNA |
|  | | |
| Orphan118 | NA | 5aj3_A: MITORIBOSOMAL 12S RRNA |
|  | | |
| Orphan119 | NA | 5aj3_A: MITORIBOSOMAL 12S RRNA |
|  | | |
| Orphan120 | NA | 5aj3_A: MITORIBOSOMAL 12S RRNA |
|  | | |
| Orphan121 | NA | 5aj3_A: MITORIBOSOMAL 12S RRNA |
|  | | |
| Orphan122 | NA | 5aj3_A: MITORIBOSOMAL 12S RRNA |
|  | | |
| Orphan123 | NA | 5aj3_A: MITORIBOSOMAL 12S RRNA |
|  | | |
| Orphan124 | NA | 5aj3_A: MITORIBOSOMAL 12S RRNA |
|  | | |
| Orphan125 | NA | 5aka_B: 23S RNA |
|  | | |
| Orphan126 | NA | 5aka_B: 23S RNA |
|  | | |
| Orphan127 | NA | 5aka_B: 23S RNA |
