# Supplementary material for: RStrucFam: a web server to associate structure and cognate RNA for RNA-binding proteins from sequence information
Source: BMC Bioinformatics. 2016 Oct 7;17:411. doi: 10.1186/s12859-016-1289-x (PMC5054549; doi:10.1186/s12859-016-1289-x)
Supplement: Additional file 2: — Details of proteins used as the negative test set. (DOC 150 kb) [file 12859_2016_1289_MOESM2_ESM.doc]

**Additional File 2:** Details of proteins used as the negative test set.

| **Entry** | **Protein names** | **Organism** | **Gene ontology (molecular function)** |
| --- | --- | --- | --- |
| A5D7B7 | Prolyl endopeptidase FAP (EC 3.4.21.26) (Dipeptidyl peptidase FAP) (EC 3.4.14.5) (Fibroblast activation protein alpha) (FAPalpha) (Gelatine degradation protease FAP) (EC 3.4.21.-) (Integral membrane serine protease) (Post-proline cleaving enzyme) (Serine integral membrane protease) (SIMP) (Surface-expressed protease) (Seprase) (Z-Pro-prolinal insensitive peptidase) (ZIP) [Cleaved into: Antiplasmin-cleaving enzyme FAP, soluble form (APCE) (EC 3.4.14.5) (EC 3.4.21.-) (EC 3.4.21.26)] | *Bos taurus* (Bovine) | dipeptidyl-peptidase activity [GO:0008239]; serine-type endopeptidase activity [GO:0004252] |
| A8DTP1 | Cytochrome c oxidase subunit 1 (EC 1.9.3.1) (Fragment) | *Aoraki denticulata* | cytochrome-c oxidase activity [GO:0004129]; heme binding [GO:0020037]; iron ion binding [GO:0005506] |
| H0USY6 | ThermoDBP-single stranded DNA binding protein | *Thermoproteus tenax* | NA |
| O25841 | Single-stranded DNA-binding protein (SSB) | *Helicobacter pylori* (strain ATCC 700392 / 26695) (*Campylobacter pylori*) | single-stranded DNA binding [GO:0003697] |
| O43692 | Peptidase inhibitor 15 (PI-15) (25 kDa trypsin inhibitor) (p25TI) (Cysteine-rich secretory protein 8) (CRISP-8) (SugarCrisp) | *Homo sapiens* (Human) | peptidase inhibitor activity [GO:0030414] |
| O75369 | Filamin-B (FLN-B) (ABP-278) (ABP-280 homolog) (Actin-binding-like protein) (Beta-filamin) (Filamin homolog 1) (Fh1) (Filamin-3) (Thyroid autoantigen) (Truncated actin-binding protein) (Truncated ABP) | *Homo sapiens* (Human) | actin binding [GO:0003779]; identical protein binding [GO:0042802]; poly(A) RNA binding [GO:0044822] |
| O95278 | Laforin (EC 3.1.3.-) (EC 3.1.3.16) (EC 3.1.3.48) (Glucan phosphatase) (Lafora PTPase) (LAFPTPase) | *Homo sapiens* (Human) | carbohydrate phosphatase activity [GO:0019203]; protein serine/threonine phosphatase activity [GO:0004722]; protein tyrosine/serine/threonine phosphatase activity [GO:0008138]; protein tyrosine phosphatase activity [GO:0004725]; starch binding [GO:2001070] |
| P00171 | Cytochrome b5 | *Bos taurus* (Bovine) | heme binding [GO:0020037]; metal ion binding [GO:0046872] |
| P00299 | Plastocyanin A, chloroplastic (PCa) | *Populus nigra* (Lombardy poplar) | copper ion binding [GO:0005507]; electron carrier activity [GO:0009055] |
| P01019 | Angiotensinogen (Serpin A8) [Cleaved into: Angiotensin-1 (Angiotensin 1-10) (Angiotensin I) (Ang I); Angiotensin-2 (Angiotensin 1-8) (Angiotensin II) (Ang II); Angiotensin-3 (Angiotensin 2-8) (Angiotensin III) (Ang III) (Des-Asp[1]-angiotensin II); Angiotensin-4 (Angiotensin 3-8) (Angiotensin IV) (Ang IV); Angiotensin 1-9; Angiotensin 1-7; Angiotensin 1-5; Angiotensin 1-4] | *Homo sapiens* (Human) | growth factor activity [GO:0008083]; hormone activity [GO:0005179]; serine-type endopeptidase inhibitor activity [GO:0004867]; sodium channel regulator activity [GO:0017080]; superoxide-generating NADPH oxidase activator activity [GO:0016176]; type 1 angiotensin receptor binding [GO:0031702]; type 2 angiotensin receptor binding [GO:0031703] |
| P01088 | Trypsin/factor XIIA inhibitor (CHFI) (Hageman factor inhibitor) | *Zea mays* (Maize) | serine-type endopeptidase inhibitor activity [GO:0004867] |
| P02753 | Retinol-binding protein 4 (Plasma retinol-binding protein) (PRBP) (RBP) [Cleaved into: Plasma retinol-binding protein(1-182); Plasma retinol-binding protein(1-181); Plasma retinol-binding protein(1-179); Plasma retinol-binding protein(1-176)] | *Homo sapiens* (Human) | retinal binding [GO:0016918]; retinol binding [GO:0019841]; retinol transporter activity [GO:0034632] |
| P02941 | Methyl-accepting chemotaxis protein II (MCP-II) (Aspartate chemoreceptor protein) | *Salmonella typhimurium* (strain LT2 / SGSC1412 / ATCC 700720) | transmembrane signaling receptor activity [GO:0004888] |
| P03036 | Regulatory protein cro (Antirepressor) | Enterobacteria phage 434 (Bacteriophage 434) | sequence-specific DNA binding [GO:0043565] |
| P03956 | Interstitial collagenase (EC 3.4.24.7) (Fibroblast collagenase) (Matrix metalloproteinase-1) (MMP-1) [Cleaved into: 22 kDa interstitial collagenase; 27 kDa interstitial collagenase] | *Homo sapiens* (Human) | calcium ion binding [GO:0005509]; endopeptidase activity [GO:0004175]; metalloendopeptidase activity [GO:0004222]; zinc ion binding [GO:0008270] |
| P04164 | Cytochrome c-552 (Cytochrome c552) (Fragment) | *Thermus thermophilus* | electron carrier activity [GO:0009055]; heme binding [GO:0020037]; metal ion binding [GO:0046872] |
| P04418 | Endonuclease V (EC 3.2.2.17) (DNA-(apurinic or apyrimidinic site) lyase) (AP lyase) (EC 4.2.99.18) (T4 pyrimidine dimer glycosylase) (T4-Pdg) | Enterobacteria phage T4 (Bacteriophage T4) | deoxyribodipyrimidine endonucleosidase activity [GO:0033959]; DNA-(apurinic or apyrimidinic site) lyase activity [GO:0003906] |
| P04637 | Cellular tumor antigen p53 (Antigen NY-CO-13) (Phosphoprotein p53) (Tumor suppressor p53) | *Homo sapiens* (Human) | ATP binding [GO:0005524]; chaperone binding [GO:0051087]; chromatin binding [GO:0003682]; copper ion binding [GO:0005507]; core promoter sequence-specific DNA binding [GO:0001046]; damaged DNA binding [GO:0003684]; DNA binding [GO:0003677]; double-stranded DNA binding [GO:0003690]; enzyme binding [GO:0019899]; histone acetyltransferase binding [GO:0035035]; identical protein binding [GO:0042802]; p53 binding [GO:0002039]; protease binding [GO:0002020]; protein heterodimerization activity [GO:0046982]; protein kinase binding [GO:0019901]; protein N-terminus binding [GO:0047485]; protein phosphatase 2A binding [GO:0051721]; protein phosphatase binding [GO:0019903]; protein self-association [GO:0043621]; receptor tyrosine kinase binding [GO:0030971]; RNA polymerase II transcription factor activity, sequence-specific DNA binding [GO:0000981]; RNA polymerase II transcription factor binding [GO:0001085]; sequence-specific DNA binding [GO:0043565]; transcriptional activator activity, RNA polymerase II transcription regulatory region sequence-specific binding [GO:0001228]; transcription factor activity, sequence-specific DNA binding [GO:0003700]; transcription factor binding [GO:0008134]; transcription regulatory region DNA binding [GO:0044212]; ubiquitin protein ligase binding [GO:0031625]; zinc ion binding [GO:0008270] |
| P05231 | Interleukin-6 (IL-6) (B-cell stimulatory factor 2) (BSF-2) (CTL differentiation factor) (CDF) (Hybridoma growth factor) (Interferon beta-2) (IFN-beta-2) | *Homo sapiens* (Human) | cytokine activity [GO:0005125]; growth factor activity [GO:0008083]; interleukin-6 receptor binding [GO:0005138] |
| P05546 | Heparin cofactor 2 (Heparin cofactor II) (HC-II) (Protease inhibitor leuserpin-2) (HLS2) (Serpin D1) | *Homo sapiens* (Human) | endopeptidase inhibitor activity [GO:0004866]; heparin binding [GO:0008201]; serine-type endopeptidase inhibitor activity [GO:0004867] |
| P06733 | Alpha-enolase (EC 4.2.1.11) (2-phospho-D-glycerate hydro-lyase) (C-myc promoter-binding protein) (Enolase 1) (MBP-1) (MPB-1) (Non-neural enolase) (NNE) (Phosphopyruvate hydratase) (Plasminogen-binding protein) | *Homo sapiens* (Human) | DNA binding [GO:0003677]; GTPase binding [GO:0051020]; magnesium ion binding [GO:0000287]; phosphopyruvate hydratase activity [GO:0004634]; poly(A) RNA binding [GO:0044822]; transcription corepressor activity [GO:0003714]; transcription factor activity, sequence-specific DNA binding [GO:0003700] |
| P08185 | Corticosteroid-binding globulin (CBG) (Serpin A6) (Transcortin) | *Homo sapiens* (Human) | serine-type endopeptidase inhibitor activity [GO:0004867]; steroid binding [GO:0005496] |
| P08870 | Orotate phosphoribosyltransferase (OPRT) (OPRTase) (EC 2.4.2.10) | *Salmonella typhimurium* (strain LT2 / SGSC1412 / ATCC 700720) | magnesium ion binding [GO:0000287]; orotate phosphoribosyltransferase activity [GO:0004588] |
| P0A8U6 | Met repressor (Met regulon regulatory protein MetJ) | *Escherichia coli* (strain K12) | DNA binding [GO:0003677]; transcription factor activity, sequence-specific DNA binding [GO:0003700] |
| P10275 | Androgen receptor (Dihydrotestosterone receptor) (Nuclear receptor subfamily 3 group C member 4) | *Homo sapiens* (Human) | androgen binding [GO:0005497]; androgen receptor activity [GO:0004882]; ATPase binding [GO:0051117]; beta-catenin binding [GO:0008013]; chromatin binding [GO:0003682]; DNA binding [GO:0003677]; enzyme binding [GO:0019899]; protein dimerization activity [GO:0046983]; receptor binding [GO:0005102]; RNA polymerase II core promoter proximal region sequence-specific DNA binding [GO:0000978]; RNA polymerase II transcription factor activity, ligand-activated sequence-specific DNA binding [GO:0004879]; RNA polymerase II transcription factor binding [GO:0001085]; transcriptional activator activity, RNA polymerase II core promoter proximal region sequence-specific binding [GO:0001077]; transcription factor activity, sequence-specific DNA binding [GO:0003700]; transcription factor binding [GO:0008134]; transcription regulatory region DNA binding [GO:0044212]; zinc ion binding [GO:0008270] |
| P10909 | Clusterin (Aging-associated gene 4 protein) (Apolipoprotein J) (Apo-J) (Complement cytolysis inhibitor) (CLI) (Complement-associated protein SP-40,40) (Ku70-binding protein 1) (NA1/NA2) (Testosterone-repressed prostate message 2) (TRPM-2) [Cleaved into: Clusterin beta chain (ApoJalpha) (Complement cytolysis inhibitor a chain); Clusterin alpha chain (ApoJbeta) (Complement cytolysis inhibitor b chain)] | *Homo sapiens* (Human) | chaperone binding [GO:0051087]; misfolded protein binding [GO:0051787]; ubiquitin protein ligase binding [GO:0031625] |
| P12883 | Myosin-7 (Myosin heavy chain 7) (Myosin heavy chain slow isoform) (MyHC-slow) (Myosin heavy chain, cardiac muscle beta isoform) (MyHC-beta) | *Homo sapiens* (Human) | actin-dependent ATPase activity [GO:0030898]; ATPase activity [GO:0016887]; ATP binding [GO:0005524]; microfilament motor activity [GO:0000146] |
| P13002 | Protein grainyhead (DNA-binding protein ELF-1) (Element I-binding activity) (Protein grainy-head) (Transcription factor NTF-1) | *Drosophila melanogaster* (Fruit fly) | DNA binding [GO:0003677]; protein homodimerization activity [GO:0042803]; sequence-specific DNA binding [GO:0043565]; transcription factor activity, sequence-specific DNA binding [GO:0003700] |
| P14164 | ARS-binding factor 1 (Bidirectionally acting factor 1) (DNA replication enhancer-binding protein OBF1) (SFB-B) | *Saccharomyces cerevisiae* (strain ATCC 204508 / S288c) (Baker's yeast) | DNA replication origin binding [GO:0003688]; RNA polymerase II core promoter proximal region sequence-specific DNA binding [GO:0000978]; sequence-specific DNA binding [GO:0043565]; sequence-specific DNA binding, bending [GO:0044374]; transcriptional activator activity, RNA polymerase II core promoter proximal region sequence-specific binding [GO:0001077] |
| P14756 | Elastase (EC 3.4.24.26) (Neutral metalloproteinase) (PAE) (Pseudolysin) [Cleaved into: Pro-elastase] | *Pseudomonas aeruginosa* (strain ATCC 15692 / PAO1 / 1C / PRS 101 / LMG 12228) | metal ion binding [GO:0046872]; metalloendopeptidase activity [GO:0004222] |
| P15381 | Voltage-dependent L-type calcium channel subunit alpha-1C (Calcium channel, L type, alpha-1 polypeptide, isoform 1, cardiac muscle) (Smooth muscle calcium channel blocker receptor) (CACB-receptor) (Voltage-gated calcium channel subunit alpha Cav1.2) | *Oryctolagus cuniculus* (Rabbit) | calmodulin binding [GO:0005516]; metal ion binding [GO:0046872]; voltage-gated calcium channel activity [GO:0005245] |
| P15976 | Erythroid transcription factor (Eryf1) (GATA-binding factor 1) (GATA-1) (GF-1) (NF-E1 DNA-binding protein) | *Homo sapiens* (Human) | C2H2 zinc finger domain binding [GO:0070742]; chromatin DNA binding [GO:0031490]; core promoter binding [GO:0001047]; DNA binding [GO:0003677]; DNA binding, bending [GO:0008301]; enhancer sequence-specific DNA binding [GO:0001158]; RNA polymerase II core promoter proximal region sequence-specific DNA binding [GO:0000978]; RNA polymerase II core promoter sequence-specific DNA binding [GO:0000979]; RNA polymerase II regulatory region sequence-specific DNA binding [GO:0000977]; RNA polymerase II transcription factor binding [GO:0001085]; sequence-specific DNA binding [GO:0043565]; transcriptional activator activity, RNA polymerase II core promoter proximal region sequence-specific binding [GO:0001077]; transcriptional activator activity, RNA polymerase II transcription regulatory region sequence-specific binding [GO:0001228]; transcriptional repressor activity, RNA polymerase II core promoter proximal region sequence-specific binding [GO:0001078]; transcription factor activity, sequence-specific DNA binding [GO:0003700]; transcription regulatory region sequence-specific DNA binding [GO:0000976]; zinc ion binding [GO:0008270] |
| P17679 | Erythroid transcription factor (Eryf1) (GATA-binding factor 1) (GATA-1) (GF-1) (NF-E1 DNA-binding protein) | *Mus musculus* (Mouse) | C2H2 zinc finger domain binding [GO:0070742]; chromatin binding [GO:0003682]; chromatin DNA binding [GO:0031490]; core promoter binding [GO:0001047]; DNA binding [GO:0003677]; DNA binding, bending [GO:0008301]; enhancer sequence-specific DNA binding [GO:0001158]; p53 binding [GO:0002039]; RNA polymerase II core promoter proximal region sequence-specific DNA binding [GO:0000978]; RNA polymerase II core promoter sequence-specific DNA binding [GO:0000979]; RNA polymerase II regulatory region sequence-specific DNA binding [GO:0000977]; RNA polymerase II transcription factor activity, sequence-specific DNA binding [GO:0000981]; RNA polymerase II transcription factor binding [GO:0001085]; sequence-specific DNA binding [GO:0043565]; transcriptional activator activity, RNA polymerase II core promoter proximal region sequence-specific binding [GO:0001077]; transcriptional repressor activity, RNA polymerase II core promoter proximal region sequence-specific binding [GO:0001078]; transcription factor activity, sequence-specific DNA binding [GO:0003700]; transcription regulatory region sequence-specific DNA binding [GO:0000976]; zinc ion binding [GO:0008270] |
| P17861 | X-box-binding protein 1 (XBP-1) (Tax-responsive element-binding protein 5) (TREB-5) [Cleaved into: X-box-binding protein 1, cytoplasmic form; X-box-binding protein 1, luminal form] | *Homo sapiens* (Human) | chromatin DNA binding [GO:0031490]; core promoter binding [GO:0001047]; DNA binding [GO:0003677]; enhancer sequence-specific DNA binding [GO:0001158]; estrogen receptor binding [GO:0030331]; identical protein binding [GO:0042802]; protease binding [GO:0002020]; protein heterodimerization activity [GO:0046982]; protein homodimerization activity [GO:0042803]; protein kinase binding [GO:0019901]; RNA polymerase II regulatory region sequence-specific DNA binding [GO:0000977]; RNA polymerase II transcription factor activity, sequence-specific DNA binding [GO:0000981]; transcription factor activity, sequence-specific DNA binding [GO:0003700]; transcription regulatory region DNA binding [GO:0044212]; ubiquitin protein ligase binding [GO:0031625] |
| P17931 | Galectin-3 (Gal-3) (35 kDa lectin) (Carbohydrate-binding protein 35) (CBP 35) (Galactose-specific lectin 3) (Galactoside-binding protein) (GALBP) (IgE-binding protein) (L-31) (Laminin-binding protein) (Lectin L-29) (Mac-2 antigen) | *Homo sapiens* (Human) | carbohydrate binding [GO:0030246]; chemoattractant activity [GO:0042056]; IgE binding [GO:0019863]; laminin binding [GO:0043236]; poly(A) RNA binding [GO:0044822] |
| P18848 | Cyclic AMP-dependent transcription factor ATF-4 (cAMP-dependent transcription factor ATF-4) (Activating transcription factor 4) (Cyclic AMP-responsive element-binding protein 2) (CREB-2) (cAMP-responsive element-binding protein 2) (DNA-binding protein TAXREB67) (Tax-responsive enhancer element-binding protein 67) (TaxREB67) | *Homo sapiens* (Human) | core promoter sequence-specific DNA binding [GO:0001046]; DNA binding [GO:0003677]; leucine zipper domain binding [GO:0043522]; protein heterodimerization activity [GO:0046982]; RNA polymerase II core promoter proximal region sequence-specific DNA binding [GO:0000978]; RNA polymerase II regulatory region sequence-specific DNA binding [GO:0000977]; RNA polymerase II transcription factor activity, sequence-specific DNA binding [GO:0000981]; RNA polymerase II transcription factor binding [GO:0001085]; sequence-specific DNA binding [GO:0043565]; transcriptional activator activity, RNA polymerase II core promoter proximal region sequence-specific binding [GO:0001077]; transcription factor activity, RNA polymerase II transcription factor binding [GO:0001076]; transcription factor activity, sequence-specific DNA binding [GO:0003700]; transcription regulatory region DNA binding [GO:0044212] |
| P19524 | Myosin-2 (Cell division control protein 66) (Class V unconventional myosin MYO2) (Type V myosin heavy chain MYO2) (Myosin V MYO2) | *Saccharomyces cerevisiae* (strain ATCC 204508 / S288c) (Baker's yeast) | actin filament binding [GO:0051015]; ATP binding [GO:0005524]; calmodulin binding [GO:0005516]; microfilament motor activity [GO:0000146] |
| P20263 | POU domain, class 5, transcription factor 1 (NF-A3) (Octamer-binding protein 3) (Oct-3) (Octamer-binding protein 4) (Oct-4) (Octamer-binding transcription factor 3) (OTF-3) | *Mus musculus* (Mouse) | chromatin binding [GO:0003682]; chromatin DNA binding [GO:0031490]; cytokine binding [GO:0019955]; DNA binding [GO:0003677]; enhancer sequence-specific DNA binding [GO:0001158]; miRNA binding [GO:0035198]; poly(A) RNA binding [GO:0044822]; protein heterodimerization activity [GO:0046982]; RNA polymerase II core promoter proximal region sequence-specific DNA binding [GO:0000978]; RNA polymerase II intronic transcription regulatory region sequence-specific DNA binding [GO:0001162]; RNA polymerase II transcription coactivator activity [GO:0001105]; RNA polymerase II transcription factor activity, sequence-specific DNA binding [GO:0000981]; sequence-specific DNA binding [GO:0043565]; transcriptional activator activity, RNA polymerase II core promoter proximal region sequence-specific binding [GO:0001077]; transcriptional repressor activity, RNA polymerase II transcription regulatory region sequence-specific binding [GO:0001227]; transcription corepressor activity [GO:0003714]; transcription factor activity, sequence-specific DNA binding [GO:0003700]; transcription factor binding [GO:0008134]; transcription regulatory region DNA binding [GO:0044212]; transcription regulatory region sequence-specific DNA binding [GO:0000976]; ubiquitin protein ligase binding [GO:0031625] |
| P21333 | Filamin-A (FLN-A) (Actin-binding protein 280) (ABP-280) (Alpha-filamin) (Endothelial actin-binding protein) (Filamin-1) (Non-muscle filamin) | *Homo sapiens* (Human) | actin filament binding [GO:0051015]; Fc-gamma receptor I complex binding [GO:0034988]; glycoprotein binding [GO:0001948]; G-protein coupled receptor binding [GO:0001664]; kinase binding [GO:0019900]; poly(A) RNA binding [GO:0044822]; protein homodimerization activity [GO:0042803]; Rac GTPase binding [GO:0048365]; Ral GTPase binding [GO:0017160]; Rho GTPase binding [GO:0017048]; signal transducer activity [GO:0004871]; small GTPase binding [GO:0031267]; transcription factor binding [GO:0008134] |
| P21580 | Tumor necrosis factor alpha-induced protein 3 (TNF alpha-induced protein 3) (EC 3.4.19.12) (EC 6.3.2.-) (OTU domain-containing protein 7C) (Putative DNA-binding protein A20) (Zinc finger protein A20) [Cleaved into: A20p50; A20p37] | *Homo sapiens* (Human) | DNA binding [GO:0003677]; identical protein binding [GO:0042802]; K63-linked polyubiquitin binding [GO:0070530]; ligase activity [GO:0016874]; protease binding [GO:0002020]; protein self-association [GO:0043621]; thiol-dependent ubiquitin-specific protease activity [GO:0004843]; ubiquitin binding [GO:0043130]; ubiquitin-protein transferase activity [GO:0004842]; zinc ion binding [GO:0008270] |
| P23687 | Prolyl endopeptidase (PE) (EC 3.4.21.26) (Post-proline cleaving enzyme) | *Sus scrofa* (Pig) | serine-type endopeptidase activity [GO:0004252]; serine-type exopeptidase activity [GO:0070008] |
| P24528 | Methylated-DNA--protein-cysteine methyltransferase (EC 2.1.1.63) (6-O-methylguanine-DNA methyltransferase) (MGMT) (O-6-methylguanine-DNA-alkyltransferase) | *Rattus norvegicus* (Rat) | calcium ion binding [GO:0005509]; DNA binding [GO:0003677]; methylated-DNA-[protein]-cysteine S-methyltransferase activity [GO:0003908] |
| P26358 | DNA (cytosine-5)-methyltransferase 1 (Dnmt1) (EC 2.1.1.37) (CXXC-type zinc finger protein 9) (DNA methyltransferase HsaI) (DNA MTase HsaI) (M.HsaI) (MCMT) | *Homo sapiens* (Human) | DNA (cytosine-5-)-methyltransferase activity [GO:0003886]; DNA (cytosine-5-)-methyltransferase activity, acting on CpG substrates [GO:0051718]; DNA binding [GO:0003677]; DNA-methyltransferase activity [GO:0009008]; double-stranded DNA binding [GO:0003690]; methyl-CpG binding [GO:0008327]; promoter-specific chromatin binding [GO:1990841]; RNA binding [GO:0003723]; unmethylated CpG binding [GO:0045322]; zinc ion binding [GO:0008270] |
| P26509 | Endo-polygalacturonase (EC 3.2.1.15) | *Pectobacterium* sp. (strain SCC3193) | polygalacturonase activity [GO:0004650] |
| P27695 | DNA-(apurinic or apyrimidinic site) lyase (EC 3.1.-.-) (EC 4.2.99.18) (APEX nuclease) (APEN) (Apurinic-apyrimidinic endonuclease 1) (AP endonuclease 1) (APE-1) (REF-1) (Redox factor-1) [Cleaved into: DNA-(apurinic or apyrimidinic site) lyase, mitochondrial] | *Homo sapiens* (Human) | 3'-5' exonuclease activity [GO:0008408]; chromatin DNA binding [GO:0031490]; damaged DNA binding [GO:0003684]; DNA-(apurinic or apyrimidinic site) lyase activity [GO:0003906]; DNA binding [GO:0003677]; double-stranded DNA 3'-5' exodeoxyribonuclease activity [GO:0008311]; double-stranded DNA exodeoxyribonuclease activity [GO:0008309]; double-stranded telomeric DNA binding [GO:0003691]; endodeoxyribonuclease activity [GO:0004520]; endonuclease activity [GO:0004519]; metal ion binding [GO:0046872]; oxidoreductase activity [GO:0016491]; phosphodiesterase I activity [GO:0004528]; phosphoric diester hydrolase activity [GO:0008081]; poly(A) RNA binding [GO:0044822]; RNA-DNA hybrid ribonuclease activity [GO:0004523]; site-specific endodeoxyribonuclease activity, specific for altered base [GO:0016890]; transcription coactivator activity [GO:0003713]; transcription corepressor activity [GO:0003714]; uracil DNA N-glycosylase activity [GO:0004844] |
| P28033 | CCAAT/enhancer-binding protein beta (C/EBP beta) (AGP/EBP) (Interleukin-6-dependent-binding protein) (IL-6DBP) (Liver-enriched transcriptional activator) (LAP) | *Mus musculus* (Mouse) | chromatin binding [GO:0003682]; DNA binding [GO:0003677]; histone acetyltransferase binding [GO:0035035]; histone deacetylase binding [GO:0042826]; kinase binding [GO:0019900]; protein heterodimerization activity [GO:0046982]; protein homodimerization activity [GO:0042803]; RNA polymerase II core promoter proximal region sequence-specific DNA binding [GO:0000978]; RNA polymerase II core promoter sequence-specific DNA binding [GO:0000979]; RNA polymerase II regulatory region sequence-specific DNA binding [GO:0000977]; RNA polymerase II transcription factor activity, sequence-specific DNA binding [GO:0000981]; sequence-specific DNA binding [GO:0043565]; transcriptional activator activity, RNA polymerase II core promoter proximal region sequence-specific binding [GO:0001077]; transcription factor activity, RNA polymerase II distal enhancer sequence-specific binding [GO:0003705]; transcription regulatory region DNA binding [GO:0044212]; ubiquitin-like protein ligase binding [GO:0044389] |
| P29508 | Serpin B3 (Protein T4-A) (Squamous cell carcinoma antigen 1) (SCCA-1) | *Homo sapiens* (Human) | cysteine-type endopeptidase inhibitor activity [GO:0004869]; protease binding [GO:0002020]; serine-type endopeptidase inhibitor activity [GO:0004867]; virus receptor activity [GO:0001618] |
| P29590 | Protein PML (Promyelocytic leukemia protein) (RING finger protein 71) (Tripartite motif-containing protein 19) | *Homo sapiens* (Human) | cobalt ion binding [GO:0050897]; DNA binding [GO:0003677]; protein heterodimerization activity [GO:0046982]; protein homodimerization activity [GO:0042803]; SUMO binding [GO:0032183]; transcription coactivator activity [GO:0003713]; ubiquitin protein ligase binding [GO:0031625]; zinc ion binding [GO:0008270] |
| P32325 | DDK kinase regulatory subunit DBF4 (Dumbbell forming protein 4) | *Saccharomyces cerevisiae* (strain ATCC 204508 / S288c) (Baker's yeast) | DNA replication origin binding [GO:0003688]; protein serine/threonine kinase activator activity [GO:0043539]; zinc ion binding [GO:0008270] |
| P32354 | Minichromosome maintenance protein 10 (Protein DNA43) | *Saccharomyces cerevisiae* (strain ATCC 204508 / S288c) (Baker's yeast) | DNA replication origin binding [GO:0003688]; double-stranded DNA binding [GO:0003690]; metal ion binding [GO:0046872]; single-stranded DNA binding [GO:0003697] |
| P35575 | Glucose-6-phosphatase (G-6-Pase) (G6Pase) (EC 3.1.3.9) (Glucose-6-phosphatase alpha) (G6Pase-alpha) | *Homo sapiens* (Human) | glucose-6-phosphatase activity [GO:0004346]; phosphate ion binding [GO:0042301]; phosphotransferase activity, alcohol group as acceptor [GO:0016773] |
| P35638 | DNA damage-inducible transcript 3 protein (DDIT-3) (C/EBP zeta) (C/EBP-homologous protein) (CHOP) (C/EBP-homologous protein 10) (CHOP-10) (CCAAT/enhancer-binding protein homologous protein) (Growth arrest and DNA damage-inducible protein GADD153) | *Homo sapiens* (Human) | cAMP response element binding protein binding [GO:0008140]; DNA binding [GO:0003677]; leucine zipper domain binding [GO:0043522]; protein heterodimerization activity [GO:0046982]; RNA polymerase II core promoter proximal region sequence-specific DNA binding [GO:0000978]; transcriptional activator activity, RNA polymerase II core promoter proximal region sequence-specific binding [GO:0001077]; transcription corepressor activity [GO:0003714]; transcription factor activity, sequence-specific DNA binding [GO:0003700]; transcription factor binding [GO:0008134]; transcription regulatory region DNA binding [GO:0044212]; transcription regulatory region sequence-specific DNA binding [GO:0000976] |
| P41594 | Metabotropic glutamate receptor 5 (mGluR5) | *Homo sapiens* (Human) | glutamate receptor activity [GO:0008066]; G-protein coupled receptor activity [GO:0004930] |
| P42224 | Signal transducer and activator of transcription 1-alpha/beta (Transcription factor ISGF-3 components p91/p84) | *Homo sapiens* (Human) | double-stranded DNA binding [GO:0003690]; enzyme binding [GO:0019899]; identical protein binding [GO:0042802]; protein homodimerization activity [GO:0042803]; RNA polymerase II core promoter proximal region sequence-specific DNA binding [GO:0000978]; RNA polymerase II core promoter sequence-specific DNA binding [GO:0000979]; signal transducer activity [GO:0004871]; transcription factor activity, RNA polymerase II core promoter sequence-specific [GO:0000983]; transcription factor activity, sequence-specific DNA binding [GO:0003700]; tumor necrosis factor receptor binding [GO:0005164] |
| P53397 | N-glycosylase/DNA lyase [Includes: 8-oxoguanine DNA glycosylase (EC 3.2.2.-); DNA-(apurinic or apyrimidinic site) lyase (AP lyase) (EC 4.2.99.18)] | *Saccharomyces cerevisiae* (strain ATCC 204508 / S288c) (Baker's yeast) | 8-oxo-7,8-dihydroguanine DNA N-glycosylase activity [GO:0034039]; damaged DNA binding [GO:0003684]; oxidized purine nucleobase lesion DNA N-glycosylase activity [GO:0008534] |
| P54274 | Telomeric repeat-binding factor 1 (NIMA-interacting protein 2) (TTAGGG repeat-binding factor 1) (Telomeric protein Pin2/TRF1) | *Homo sapiens* (Human) | DNA binding [GO:0003677]; DNA binding, bending [GO:0008301]; double-stranded telomeric DNA binding [GO:0003691]; G-rich strand telomeric DNA binding [GO:0098505]; microtubule binding [GO:0008017]; protein heterodimerization activity [GO:0046982]; protein homodimerization activity [GO:0042803]; telomeric DNA binding [GO:0042162]; ubiquitin binding [GO:0043130] |
| P59632 | Protein 3a (Accessory protein 3a) (Protein U274) (Protein X1) | Human SARS coronavirus (SARS-CoV) (Severe acute respiratory syndrome coronavirus) | ion channel activity [GO:0005216] |
| P60484 | Phosphatidylinositol 3,4,5-trisphosphate 3-phosphatase and dual-specificity protein phosphatase PTEN (EC 3.1.3.16) (EC 3.1.3.48) (EC 3.1.3.67) (Mutated in multiple advanced cancers 1) (Phosphatase and tensin homolog) | *Homo sapiens* (Human) | anaphase-promoting complex binding [GO:0010997]; enzyme binding [GO:0019899]; identical protein binding [GO:0042802]; inositol-1,3,4,5-tetrakisphosphate 3-phosphatase activity [GO:0051717]; lipid binding [GO:0008289]; magnesium ion binding [GO:0000287]; PDZ domain binding [GO:0030165]; phosphatidylinositol-3,4,5-trisphosphate 3-phosphatase activity [GO:0016314]; phosphatidylinositol-3,4-bisphosphate 3-phosphatase activity [GO:0051800]; phosphatidylinositol-3-phosphatase activity [GO:0004438]; phosphoprotein phosphatase activity [GO:0004721]; protein serine/threonine phosphatase activity [GO:0004722]; protein tyrosine/serine/threonine phosphatase activity [GO:0008138]; protein tyrosine phosphatase activity [GO:0004725] |
| P70662 | LIM domain-binding protein 1 (LDB-1) (Carboxyl-terminal LIM domain-binding protein 2) (CLIM-2) (LIM domain-binding factor CLIM2) (mLdb1) (Nuclear LIM interactor) | *Mus musculus* (Mouse) | chromatin binding [GO:0003682]; enhancer sequence-specific DNA binding [GO:0001158]; enzyme binding [GO:0019899]; LIM domain binding [GO:0030274]; protein homodimerization activity [GO:0042803]; protein self-association [GO:0043621]; RNA polymerase II activating transcription factor binding [GO:0001102]; transcription factor activity, transcription factor binding [GO:0000989] |
| Q01826 | DNA-binding protein SATB1 (Special AT-rich sequence-binding protein 1) | *Homo sapiens* (Human) | chromatin binding [GO:0003682]; double-stranded DNA binding [GO:0003690]; RNA polymerase II regulatory region sequence-specific DNA binding [GO:0000977]; transcriptional repressor activity, RNA polymerase II transcription regulatory region sequence-specific binding [GO:0001227] |
| Q01842 | Ets DNA-binding protein pokkuri (Protein anterior open) (Protein yan) | *Drosophila melanogaster* (Fruit fly) | protein domain specific binding [GO:0019904]; sequence-specific DNA binding [GO:0043565]; transcription factor activity, RNA polymerase II distal enhancer sequence-specific binding [GO:0003705]; transcription factor activity, sequence-specific DNA binding [GO:0003700] |
| Q03164 | Histone-lysine N-methyltransferase 2A (Lysine N-methyltransferase 2A) (EC 2.1.1.43) (ALL-1) (CXXC-type zinc finger protein 7) (Myeloid/lymphoid or mixed-lineage leukemia) (Myeloid/lymphoid or mixed-lineage leukemia protein 1) (Trithorax-like protein) (Zinc finger protein HRX) [Cleaved into: MLL cleavage product N320 (N-terminal cleavage product of 320 kDa) (p320); MLL cleavage product C180 (C-terminal cleavage product of 180 kDa) (p180)] | *Homo sapiens* (Human) | AT DNA binding [GO:0003680]; chromatin binding [GO:0003682]; core promoter sequence-specific DNA binding [GO:0001046]; histone methyltransferase activity (H3-K4 specific) [GO:0042800]; identical protein binding [GO:0042802]; lysine-acetylated histone binding [GO:0070577]; protein homodimerization activity [GO:0042803]; transcription factor activity, sequence-specific DNA binding [GO:0003700]; transcription regulatory region DNA binding [GO:0044212]; unmethylated CpG binding [GO:0045322]; zinc ion binding [GO:0008270] |
| Q03468 | DNA excision repair protein ERCC-6 (EC 3.6.4.-) (ATP-dependent helicase ERCC6) (Cockayne syndrome protein CSB) | *Homo sapiens* (Human) | ATP binding [GO:0005524]; chromatin binding [GO:0003682]; DNA binding [GO:0003677]; DNA-dependent ATPase activity [GO:0008094]; helicase activity [GO:0004386]; protein complex binding [GO:0032403]; protein C-terminus binding [GO:0008022]; protein N-terminus binding [GO:0047485]; protein tyrosine kinase activator activity [GO:0030296] |
| Q03720 | Calcium-activated potassium channel slowpoke (dSlo) (BK channel) (Maxi K channel) (MaxiK) | *Drosophila melanogaster* (Fruit fly) | calcium-activated potassium channel activity [GO:0015269]; large conductance calcium-activated potassium channel activity [GO:0060072]; voltage-gated potassium channel activity [GO:0005249] |
| Q06JG6 | CLAVATA3/ESR (CLE)-related protein 16D10 | *Meloidogyne javanica* (Root-knot nematode worm) | NA |
| Q08884 | Narbonin | *Vicia narbonensis* (Narbonne vetch) | hydrolase activity, hydrolyzing O-glycosyl compounds [GO:0004553] |
| Q0IIB6 | DNA damage-inducible transcript 3 protein (DDIT-3) (C/EBP zeta) (C/EBP-homologous protein) (CHOP) (C/EBP-homologous protein 10) (CHOP-10) (CCAAT/enhancer-binding protein homologous protein) (Growth arrest and DNA-damage-inducible protein GADD153) | *Bos taurus* (Bovine) | DNA binding [GO:0003677]; RNA polymerase II core promoter proximal region sequence-specific DNA binding [GO:0000978]; transcriptional activator activity, RNA polymerase II core promoter proximal region sequence-specific binding [GO:0001077]; transcription corepressor activity [GO:0003714] |
| Q12809 | Potassium voltage-gated channel subfamily H member 2 (Eag homolog) (Ether-a-go-go-related gene potassium channel 1) (ERG-1) (Eag-related protein 1) (Ether-a-go-go-related protein 1) (H-ERG) (hERG-1) (hERG1) (Voltage-gated potassium channel subunit Kv11.1) | *Homo sapiens* (Human) | C3HC4-type RING finger domain binding [GO:0055131]; delayed rectifier potassium channel activity [GO:0005251]; identical protein binding [GO:0042802]; inward rectifier potassium channel activity [GO:0005242]; phosphorelay sensor kinase activity [GO:0000155]; protein homodimerization activity [GO:0042803]; scaffold protein binding [GO:0097110]; ubiquitin protein ligase binding [GO:0031625]; voltage-gated potassium channel activity [GO:0005249]; voltage-gated potassium channel activity involved in cardiac muscle cell action potential repolarization [GO:0086008]; voltage-gated potassium channel activity involved in ventricular cardiac muscle cell action potential repolarization [GO:1902282] |
| Q12884 | Prolyl endopeptidase FAP (EC 3.4.21.26) (170 kDa melanoma membrane-bound gelatinase) (Dipeptidyl peptidase FAP) (EC 3.4.14.5) (Fibroblast activation protein alpha) (FAPalpha) (Gelatine degradation protease FAP) (EC 3.4.21.-) (Integral membrane serine protease) (Post-proline cleaving enzyme) (Serine integral membrane protease) (SIMP) (Surface-expressed protease) (Seprase) [Cleaved into: Antiplasmin-cleaving enzyme FAP, soluble form (APCE) (EC 3.4.14.5) (EC 3.4.21.-) (EC 3.4.21.26)] | *Homo sapiens* (Human) | dipeptidyl-peptidase activity [GO:0008239]; endopeptidase activity [GO:0004175]; integrin binding [GO:0005178]; metalloendopeptidase activity [GO:0004222]; peptidase activity [GO:0008233]; protease binding [GO:0002020]; protein dimerization activity [GO:0046983]; protein homodimerization activity [GO:0042803]; serine-type endopeptidase activity [GO:0004252]; serine-type peptidase activity [GO:0008236] |
| Q13469 | Nuclear factor of activated T-cells, cytoplasmic 2 (NF-ATc2) (NFATc2) (NFAT pre-existing subunit) (NF-ATp) (T-cell transcription factor NFAT1) | *Homo sapiens* (Human) | chromatin binding [GO:0003682]; DNA binding [GO:0003677]; RNA polymerase II core promoter proximal region sequence-specific DNA binding [GO:0000978]; transcriptional activator activity, RNA polymerase II core promoter proximal region sequence-specific binding [GO:0001077]; transcriptional repressor activity, RNA polymerase II core promoter proximal region sequence-specific binding [GO:0001078]; transcription factor activity, sequence-specific DNA binding [GO:0003700] |
| Q13501 | Sequestosome-1 (EBI3-associated protein of 60 kDa) (EBIAP) (p60) (Phosphotyrosine-independent ligand for the Lck SH2 domain of 62 kDa) (Ubiquitin-binding protein p62) | *Homo sapiens* (Human) | identical protein binding [GO:0042802]; protein kinase binding [GO:0019901]; protein kinase C binding [GO:0005080]; protein serine/threonine kinase activity [GO:0004674]; receptor tyrosine kinase binding [GO:0030971]; SH2 domain binding [GO:0042169]; ubiquitin binding [GO:0043130]; ubiquitin protein ligase binding [GO:0031625]; zinc ion binding [GO:0008270] |
| Q15848 | Adiponectin (30 kDa adipocyte complement-related protein) (Adipocyte complement-related 30 kDa protein) (ACRP30) (Adipocyte, C1q and collagen domain-containing protein) (Adipose most abundant gene transcript 1 protein) (apM-1) (Gelatin-binding protein) | *Homo sapiens* (Human) | cytokine activity [GO:0005125]; hormone activity [GO:0005179]; identical protein binding [GO:0042802]; protein homodimerization activity [GO:0042803]; receptor binding [GO:0005102]; sialic acid binding [GO:0033691] |
| Q1XA76 | Acid-sensing ion channel 1 (ASIC1) (Amiloride-sensitive cation channel 2, neuronal) | *Gallus gallus* (Chicken) | acid-sensing ion channel activity [GO:0044736]; ion gated channel activity [GO:0022839] |
| Q42328 | Defensin-like protein 195 (ATTp) (Trypsin inhibitor ATTI-1) (diDi 4T-1) | *Arabidopsis thaliana* (Mouse-ear cress) | ion channel inhibitor activity [GO:0008200]; serine-type endopeptidase inhibitor activity [GO:0004867] |
| Q62226 | Sonic hedgehog protein (SHH) (HHG-1) [Cleaved into: Sonic hedgehog protein N-product (Sonic hedgehog protein 19 kDa product); Sonic hedgehog protein C-product (Sonic hedgehog protein 27 kDa product)] | *Mus musculus* (Mouse) | calcium ion binding [GO:0005509]; glycoprotein binding [GO:0001948]; glycosaminoglycan binding [GO:0005539]; laminin-1 binding [GO:0043237]; patched binding [GO:0005113]; zinc ion binding [GO:0008270] |
| Q7KMQ6 | IP16020p (Phosphatase PTEN) (Phosphatase and tensin homolog, isoform B) (Phosphatase and tensin homolog, isoform D) (EC 3.1.3.-) (EC 3.1.3.16) (EC 3.1.3.48) (EC 3.1.3.64) (EC 3.1.3.67) (Phosphatase and tensin homolog, isoform G) (Phosphatase and tensin homolog, isoform H) | *Drosophila melanogaster* (Fruit fly) | actin binding [GO:0003779]; non-membrane spanning protein tyrosine phosphatase activity [GO:0004726]; phosphatidylinositol-3,4,5-trisphosphate 3-phosphatase activity [GO:0016314]; phosphatidylinositol-3-phosphatase activity [GO:0004438]; phosphoprotein phosphatase activity [GO:0004721]; protein serine/threonine phosphatase activity [GO:0004722]; protein tyrosine/serine/threonine phosphatase activity [GO:0008138]; protein tyrosine phosphatase activity [GO:0004725] |
| Q7Y265 | Gp32 (Gp32 single-stranded DNA binding protein) | Enterobacteria phage RB69 (Bacteriophage RB69) | metal ion binding [GO:0046872]; single-stranded DNA binding [GO:0003697] |
| Q86AS5 | Prolyl endopeptidase (PE) (EC 3.4.21.26) (POase) (Post-proline cleaving enzyme) | *Dictyostelium discoideum* (Slime mold) | oligopeptidase activity [GO:0070012]; serine-type endopeptidase activity [GO:0004252]; serine-type exopeptidase activity [GO:0070008] |
| Q8IZP0 | Abl interactor 1 (Abelson interactor 1) (Abi-1) (Abl-binding protein 4) (AblBP4) (Eps8 SH3 domain-binding protein) (Eps8-binding protein) (Nap1-binding protein) (Nap1BP) (Spectrin SH3 domain-binding protein 1) (e3B1) | *Homo sapiens* (Human) | cytoskeletal protein binding [GO:0008092]; protein complex binding [GO:0032403]; protein tyrosine kinase activator activity [GO:0030296] |
| Q92673 | Sortilin-related receptor (Low-density lipoprotein receptor relative with 11 ligand-binding repeats) (LDLR relative with 11 ligand-binding repeats) (LR11) (SorLA-1) (Sorting protein-related receptor containing LDLR class A repeats) (SorLA) | *Homo sapiens* (Human) | ADP-ribosylation factor binding [GO:0030306]; beta-amyloid binding [GO:0001540]; low-density lipoprotein particle binding [GO:0030169]; transmembrane signaling receptor activity [GO:0004888] |
| Q92878 | DNA repair protein RAD50 (hRAD50) (EC 3.6.-.-) | *Homo sapiens* (Human) | ATPase activity [GO:0016887]; ATP binding [GO:0005524]; DNA binding [GO:0003677]; metal ion binding [GO:0046872]; protein binding, bridging [GO:0030674] |
| Q94KI8 | Two pore calcium channel protein 1 (Calcium channel protein 1) (AtCCH1) (Fatty acid oxygenation up-regulated protein 2) (Voltage-dependent calcium channel protein TPC1) (AtTPC1) | *Arabidopsis thaliana* (Mouse-ear cress) | calcium ion binding [GO:0005509]; voltage-gated calcium channel activity [GO:0005245] |
| Q95V25 | Calcium-activated potassium channel slo-1 (BK channel) (Maxi K channel) (MaxiK) (Slo homolog) (Slowpoke protein 1) | *Caenorhabditis elegans* | calcium-activated potassium channel activity [GO:0015269]; large conductance calcium-activated potassium channel activity [GO:0060072]; voltage-gated potassium channel activity [GO:0005249] |
| Q96JB5 | CDK5 regulatory subunit-associated protein 3 (CDK5 activator-binding protein C53) (LXXLL/leucine-zipper-containing ARF-binding protein) (Protein HSF-27) | *Homo sapiens* (Human) | cyclin binding [GO:0030332]; MDM2/MDM4 family protein binding [GO:0097371]; mitogen-activated protein kinase binding [GO:0051019]; NF-kappaB binding [GO:0051059]; protein kinase binding [GO:0019901]; ubiquitin-like protein ligase binding [GO:0044389] |
| Q99081 | Transcription factor 12 (TCF-12) (Class B basic helix-loop-helix protein 20) (bHLHb20) (DNA-binding protein HTF4) (E-box-binding protein) (Transcription factor HTF-4) | *Homo sapiens* (Human) | bHLH transcription factor binding [GO:0043425]; cAMP response element binding [GO:0035497]; E-box binding [GO:0070888]; enhancer binding [GO:0035326]; protein heterodimerization activity [GO:0046982]; protein homodimerization activity [GO:0042803]; RNA polymerase II core promoter proximal region sequence-specific DNA binding [GO:0000978]; SMAD binding [GO:0046332]; transcriptional activator activity, RNA polymerase II core promoter proximal region sequence-specific binding [GO:0001077]; transcription factor activity, sequence-specific DNA binding [GO:0003700]; transcription factor binding [GO:0008134] |
| Q99323 | Myosin heavy chain, non-muscle (Myosin II) (Non-muscle MHC) (Zipper protein) | *Drosophila melanogaster* (Fruit fly) | ATPase activity, coupled [GO:0042623]; ATP binding [GO:0005524]; motor activity [GO:0003774]; myosin light chain binding [GO:0032027] |
| Q99417 | C-Myc-binding protein (Associate of Myc 1) (AMY-1) | *Homo sapiens* (Human) | transcription coactivator activity [GO:0003713] |
| Q99497 | Protein deglycase DJ-1 (DJ-1) (EC 3.1.2.-) (EC 3.5.1.-) (Oncogene DJ1) (Parkinson disease protein 7) | *Homo sapiens* (Human) | androgen receptor binding [GO:0050681]; copper ion binding [GO:0005507]; cupric ion binding [GO:1903135]; cuprous ion binding [GO:1903136]; cytokine binding [GO:0019955]; enzyme binding [GO:0019899]; glyoxalase (glycolic acid-forming) activity [GO:1990422]; glyoxalase III activity [GO:0019172]; identical protein binding [GO:0042802]; kinase binding [GO:0019900]; L-dopa decarboxylase activator activity [GO:0036478]; mercury ion binding [GO:0045340]; mRNA binding [GO:0003729]; oxidoreductase activity, acting on peroxide as acceptor [GO:0016684]; peptidase activity [GO:0008233]; peroxiredoxin activity [GO:0051920]; protein homodimerization activity [GO:0042803]; receptor binding [GO:0005102]; repressing transcription factor binding [GO:0070491]; scaffold protein binding [GO:0097110]; small protein activating enzyme binding [GO:0044388]; superoxide dismutase copper chaperone activity [GO:0016532]; transcription coactivator activity [GO:0003713]; transcription factor binding [GO:0008134]; tyrosine 3-monooxygenase activator activity [GO:0036470]; ubiquitin-like protein conjugating enzyme binding [GO:0044390]; ubiquitin-specific protease binding [GO:1990381] |
| Q99828 | Calcium and integrin-binding protein 1 (CIB) (Calcium- and integrin-binding protein) (CIBP) (Calmyrin) (DNA-PKcs-interacting protein) (Kinase-interacting protein) (KIP) (SNK-interacting protein 2-28) (SIP2-28) | *Homo sapiens* (Human) | calcium-dependent protein kinase inhibitor activity [GO:0008427]; calcium ion binding [GO:0005509]; protein anchor [GO:0043495]; protein serine/threonine kinase inhibitor activity [GO:0030291]; Ras GTPase binding [GO:0017016] |
| Q9LD83 | Guard cell S-type anion channel SLAC1 (Protein CARBON DIOXIDE INSENSITIVE 3) (Protein OZONE-SENSITIVE 1) (Protein RADICAL-INDUCED CELL DEATH 3) (Protein SLOW ANION CHANNEL-ASSOCIATED 1) | *Arabidopsis thaliana* (Mouse-ear cress) | anion transmembrane transporter activity [GO:0008509]; protein kinase binding [GO:0019901]; protein phosphatase binding [GO:0019903]; voltage-gated anion channel activity [GO:0008308] |
| Q9PW88 | G-protein coupled receptor family C group 6 member A (Odorant receptor 5.24) | *Carassius auratus* (Goldfish) | G-protein coupled receptor activity [GO:0004930] |
| Q9UGP5 | DNA polymerase lambda (Pol Lambda) (EC 2.7.7.7) (EC 4.2.99.-) (DNA polymerase beta-2) (Pol beta2) (DNA polymerase kappa) | *Homo sapiens* (Human) | DNA binding [GO:0003677]; DNA-directed DNA polymerase activity [GO:0003887]; lyase activity [GO:0016829]; metal ion binding [GO:0046872] |
| Q9V3N1 | LD24467p (MIP27581p) (Serpin 27A) (Serpin 27A, isoform A) (Serpin 27A, isoform B) | *Drosophila melanogaster* (Fruit fly) | enzyme inhibitor activity [GO:0004857]; serine-type endopeptidase inhibitor activity [GO:0004867] |
| Q9VHQ7 | Odorant receptor 85b | *Drosophila melanogaster* (Fruit fly) | odorant binding [GO:0005549]; olfactory receptor activity [GO:0004984] |
| Q9VNB5 | Odorant receptor coreceptor (Odorant receptor 83b) | *Drosophila melanogaster* (Fruit fly) | calcium-release channel activity [GO:0015278]; calmodulin binding [GO:0005516]; odorant binding [GO:0005549]; olfactory receptor activity [GO:0004984]; protein homodimerization activity [GO:0042803] |
| Q9W252 | DNA repair protein RAD50 (EC 3.6.-.-) | *Drosophila melanogaster* (Fruit fly) | ATPase activity [GO:0016887]; ATP binding [GO:0005524]; double-stranded DNA binding [GO:0003690]; metal ion binding [GO:0046872] |
| Q9WIJ4 | Cell cycle link protein (Clink) | Faba bean necrotic yellows virus (isolate Egyptian EV1-93) (FBNYV) | NA |
| Q9WTN3 | Sterol regulatory element-binding protein 1 (SREBP-1) (Sterol regulatory element-binding transcription factor 1) [Cleaved into: Processed sterol regulatory element-binding protein 1] | *Mus musculus* (Mouse) | chromatin binding [GO:0003682]; DNA binding [GO:0003677]; protein kinase binding [GO:0019901]; RNA polymerase II core promoter proximal region sequence-specific DNA binding [GO:0000978]; sequence-specific DNA binding [GO:0043565]; sterol response element binding [GO:0032810]; transcriptional activator activity, RNA polymerase II core promoter proximal region sequence-specific binding [GO:0001077]; transcription factor activity, RNA polymerase II core promoter proximal region sequence-specific binding [GO:0000982]; transcription factor activity, sequence-specific DNA binding [GO:0003700]; transcription regulatory region DNA binding [GO:0044212] |
| Q9Y230 | RuvB-like 2 (EC 3.6.4.12) (48 kDa TATA box-binding protein-interacting protein) (48 kDa TBP-interacting protein) (51 kDa erythrocyte cytosolic protein) (ECP-51) (INO80 complex subunit J) (Repressing pontin 52) (Reptin 52) (TIP49b) (TIP60-associated protein 54-beta) (TAP54-beta) | *Homo sapiens* (Human) | ADP binding [GO:0043531]; ATP binding [GO:0005524]; ATP-dependent 5'-3' DNA helicase activity [GO:0043141]; ATP-dependent DNA helicase activity [GO:0004003]; chromatin DNA binding [GO:0031490]; damaged DNA binding [GO:0003684]; DNA helicase activity [GO:0003678]; identical protein binding [GO:0042802]; RNA polymerase II core promoter sequence-specific DNA binding [GO:0000979]; RNA polymerase II distal enhancer sequence-specific DNA binding [GO:0000980]; unfolded protein binding [GO:0051082] |
| Q9Y2R2 | Tyrosine-protein phosphatase non-receptor type 22 (EC 3.1.3.48) (Hematopoietic cell protein-tyrosine phosphatase 70Z-PEP) (Lymphoid phosphatase) (LyP) (PEST-domain phosphatase) (PEP) | *Homo sapiens* (Human) | kinase binding [GO:0019900]; phosphatase activity [GO:0016791]; protein tyrosine phosphatase activity [GO:0004725]; SH3 domain binding [GO:0017124]; ubiquitin protein ligase binding [GO:0031625] |
