# Supplementary material for: RStrucFam: a web server to associate structure and cognate RNA for RNA-binding proteins from sequence information
Source: BMC Bioinformatics. 2016 Oct 7;17:411. doi: 10.1186/s12859-016-1289-x (PMC5054549; doi:10.1186/s12859-016-1289-x)
Supplement: Additional file 3: — List of proteins used in the resubstitution test. (DOC 102 kb) [file 12859_2016_1289_MOESM3_ESM.doc]

**Additional File 3: List of proteins used in the resubstitution test.**

| **PDB chain ID** | **Parent family** | **Family identified** | **Rank of parent family** | **Verified** |
| --- | --- | --- | --- | --- |
| 3ccl_M | 54193 | 54193 | 1 | Yes |
| 1e8o_C | 54763 | 54763 | 1 | Yes |
| 1av6_A | 88785 | 88785 | 1 | Yes |
| 3dh3_C | Cluster14 | 55178 | - | No |
| 2go5_1 | 90019 | 90019 | 1 | Yes |
| 2iy5_B | 55682 | 55682 | 1 | Yes |
| 2bte_D | 191377 | 191377 | 1 | Yes |
| 2km8_B | 191529 | 191529 | 1 | Yes |
| 2ob7_C | 74983 | 74983 | 1 | Yes |
| 4bpb_A | 254188 | 254188 | 1 | Yes |
| 3j7y_8 | Cluster3 | Cluster3 | 1 | Yes |
| 3iev_A | 52592 | 54815 | 2 | Yes |
| 3j46_y | Orphan59 | Orphan59 | 1 | Yes |
| 4uer_9 | Cluster17 | Cluster17 | 1 | Yes |
| 4ox9_Y | Orphan104 | Orphan104 | 1 | Yes |
| 4oog_B | Cluster135 | Cluster135 | 1 | Yes |
| 4pjo_s | 50183 | 50183 | 1 | Yes |
| 3rtj_B | 227190 | 227190 | 1 | Yes |
| 1hnw_D | 55178 | 55178 | 1 | Yes |
| 4uer_S | 54571 | 54571 | 1 | Yes |
| 2ozb_D | 55316 | 55316 | 1 | Yes |
| 2err_A | 54929 | 54929 | 1 | Yes |
| 4d61_i | 52592 | 52592 | 1 | Yes |
| 4m7a_M | 50183 | 50183 | 1 | Yes |
| 3a6p_A | Orphan38 | Orphan38 | 1 | Yes |
| 5aj3_a | Orphan111 | Orphan111 | 1 | Yes |
| 5aj3_U | Orphan124 | Orphan124 | 1 | Yes |
| 2li8_A | Orphan18 | Orphan18 | 1 | Yes |
| 1w2b_X | 58124 | 52043 | - | No |
| 2y9h_O | 231597 | 231597 | 1 | Yes |
| 3ciy_B | 52068 | Orphan39 | 2 | Yes |
| 1n77_A | 52375 | 52375 | 1 | Yes |
| 4uer_a | Orphan107 | Orphan107 | 1 | Yes |
| 4ifd_E | 227218 | 227218 | 1 | Yes |
| 3j3w_L | Cluster126 | Cluster126 | 1 | Yes |
| 4wrt_C | Cluster101 | Cluster101 | 1 | Yes |
| 3j3w_D | 50461 | 50461 | 1 | Yes |
| 1o0b_A | 52375 | 52375 | 1 | Yes |
| 1ffk_T | 55130 | 55130 | 1 | Yes |
| 4db2_D | Cluster43 | Cluster43 | 1 | Yes |
| 3nmu_F | 53342 | 53342 | 1 | Yes |
| 1i95_Q | 50282 | Orphan 33 | 2 | Yes |
| 2gic_C | 140810 | 140810 | 1 | Yes |
| 2jlw_B | 52724 | 52724 | 1 | Yes |
| 4uer_6* | Cluster5 | Cluster5 | 1 | Yes |
| 3cma_R | 54844 | 54844 | 1 | Yes |
| 2noq_F | 47974 | 47974 | 1 | Yes |
| 2x1f_A | 191529 | 191529 | 1 | Yes |
| 4ce4_h | 54769 | 54769 | 1 | Yes |
| 2uwm_A | 74683 | 74683 | 1 | Yes |
| 2vpl_C | 56809 | 56809 | 1 | Yes |
| 2vop_A | 54929 | 54929 | 1 | Yes |
| 2w2h_D | Cluster83 | Cluster83 | 1 | Yes |
| 4oav_D | Cluster1 | Cluster1 | 1 | Yes |
| 1nyb_A | 58340 | 58340 | 1 | Yes |
| 1ysh_E | Cluster11 | No hit | - | No |
| 3q0o_B | 63611 | 63611 | 1 | Yes |
| 5aj3_m | Orphan118 | Orphan118 | 1 | Yes |
| 3j80_G | Cluster3 | Cluster3 | 1 | Yes |
| 3uzt_A | Cluster31 | Cluster31 | 1 | Yes |
| 3j3v_O | Orphan50 | Orphan50 | 1 | Yes |
| 4g0a_D | Cluster75 | Cluster75 | 1 | Yes |
| 2ez6_A | 54769 | 54769 | 1 | Yes |
| 3j3v_Y | 55130 | 55130 | 1 | Yes |
| 4xjn_I | Cluster7 | 191323 | - | No |
| 1ry1_C | 103693 | 54763 | 2 | Yes |
| 3j5l_D | 58124 | 50461 | 2 | Yes |
| 2xb2_Z | 54929 | 54929 | 1 | Yes |
| 3ftf_A | 191451 | 191451 | 1 | Yes |
| 1xbp_4 | 57841 | 57841 | 1 | Yes |
| 3cw1_A | 50183 | 50183 | 1 | Yes |
| 2fmt_A | 53329 | 53329 | 1 | Yes |
| 2ykr_U | Orphan36 | Cluster117 | 2 | Yes |
| 2wwa_O | Cluster120 | Cluster120 | 1 | Yes |
| 4kzz_A | 52314 | 52314 | 1 | Yes |
| 2go5_4 | 54190 | 54190 | 1 | Yes |
| 3ktw_A | 69696 | 69696 | 1 | Yes |
| 1f7y_A | 47064 | 47064 | 1 | Yes |
| 4uer_K | 53138 | 53138 | 1 | Yes |
| 4n0t_A | 191529 | 191529 | 1 | Yes |
| 1ysh_D | 57830 | 57830 | 1 | Yes |
| 2gje_A | 143043 | 143043 | 1 | Yes |
| 3j81_T | 46853 | 46853 | 1 | Yes |
| 2zko_A | 47061 | 47061 | 1 | Yes |
| 3j9b_A | Cluster65 | Cluster65 | 1 | Yes |
| 3iyq_B | 74983 | 74983 | 1 | Yes |
| 1lng_A | 69696 | 69696 | 1 | Yes |
| 4u7u_J | Cluster26 | Cluster26 | 1 | Yes |
| 4bhh_Z | Cluster2 | Cluster2 | 1 | Yes |
| 3j7a_T | Cluster66 | Cluster66 | 1 | Yes |
| 3zd6_A | 254188 | 254188 | 1 | Yes |
| 3zc0_J | Cluster124 | Cluster124 | 1 | Yes |
| 1efw_A | 55682 | 55682 | 1 | Yes |
| 1zh5_B | 54929 | 54929 | 1 | Yes |
| 1nkw_J | 58124 | Cluster126 | - | No |
| 3j3w_S | 58124 | 54844 | - | No |
| 3j6v_O | 58132 | 47064 | 2 | Yes |
| 1mji_B | 55283 | 55283 | 1 | Yes |
| 2du3_B | Cluster21 | Cluster108 | - | No |
| 4ce4_c | 55682 | 55682 | 1 | Yes |

* This protein identified its parent family at an E-value of 0.1.
